# Taxonomic review of the late Cenozoic megapodes (Galliformes: Megapodiidae) of Australia

**DOI:** 10.1098/rsos.170233

**Published:** 2017-06-14

**Authors:** Elen Shute, Gavin J. Prideaux, Trevor H. Worthy

**Affiliations:** School of Biological Sciences, Flinders University, GPO Box 2100, Adelaide, South Australia 5001, Australia

**Keywords:** megapodes, taxonomy, nomenclature, fossil birds, Pleistocene extinctions

## Abstract

Megapodes are unusual galliform birds that use passive heat sources to incubate their eggs. Evolutionary relationships of extant megapode taxa have become clearer with the advent of molecular analyses, but the systematics of large, extinct forms (*Progura gallinacea*, *Progura naracoortensis*) from the late Cenozoic of Australia has been a source of confusion. It was recently suggested that the two species of *Progura* were synonymous, and that this taxon dwarfed into the extant malleefowl *Leipoa ocellata* in the Late Pleistocene. Here, we review previously described fossils along with newly discovered material from several localities, and present a substantial taxonomic revision. We show that *P. gallinacea* and *P. naracoortensis* are generically distinct, describe two new species of megapode from the Thylacoleo Caves of south-central Australia, and a new genus from Curramulka Quarry in southern Australia. We also show that *L. ocellata* was contemporaneous with larger species. Our phylogenetic analysis places four extinct taxa in a derived clade with the extant Australo-Papuan brush-turkeys *Talegalla fuscirostris*, *L. ocellata*, *Alectura lathami* and *Aepypodius bruijnii*. Therefore, diversity of brush-turkeys halved during the Quaternary, matching extinction rates of scrubfowl in the Pacific. Unlike extant brush-turkeys, all the extinct taxa appear to have been burrow-nesters.

## 1. Introduction

Megapodes (Megapodiidae) are a family of galliform birds endemic to Oceania. Uniquely among birds, they do not use body-heat to incubate their eggs [1]. Rather, most species bury their eggs in large nest mounds of soil and leaf litter, which they rake together using their very large feet: heat generated by decomposition of the vegetation incubates the eggs [2]. A few species do not build mounds, and simply bury their eggs in warm sand, using either geothermal heat from volcanoes or the heat of the sun for incubation [3], while some species in the genus *Megapodius* are flexible in their nesting strategies depending on environmental circumstances [4].

There are seven extant genera of megapodes, containing 22 species: *Megapodius* (13 species), *Talegalla* (three species), *Aepypodius* (two species) and one species each in *Alectura*, *Leipoa*, *Macrocephalon* and *Eulipoa* [5]. However, it has been estimated that at least half of megapode species, mostly those on Pacific islands, have gone extinct since human colonization during the Holocene [6].

**Figure 1.**
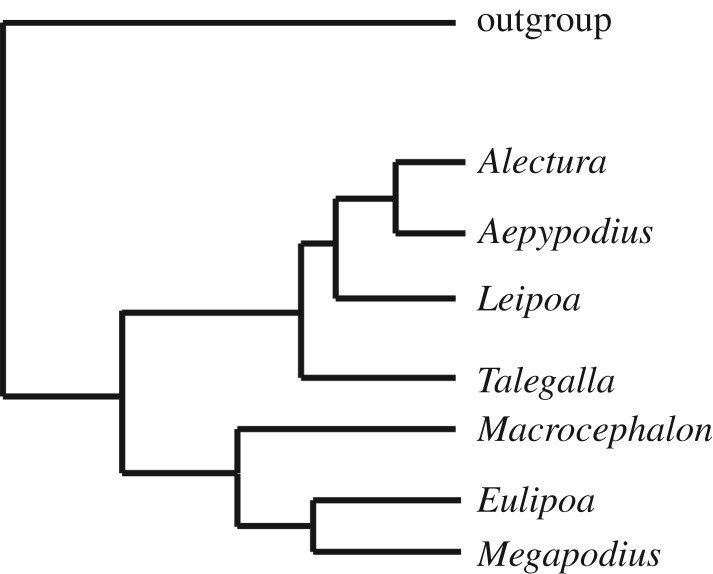
Molecular phylogeny of extant megapode genera (after Harris *et al.* [4]); *Macrocephalon* is included here as a basal member of the ‘scrubfowl’ clade.

A recent molecular phylogenetic analysis of extant megapodes identified two well-supported clades: a ‘brush-turkey’ clade containing the genera *Talegalla*, *Leipoa*, *Alectura* and *Aepypodius*; and a ‘scrubfowl’ clade of *Macrocephalon*, *Eulipoa* and *Megapodius* [4] (figure 1). In that study, which was based on 14 nuclear and two mitochondrial loci, only the position of *Macrocephalon* differed from an earlier phylogeny that used fewer loci [7], so it seems a consensus on relationships has been reached. In the more recent study, biogeographic modelling determined that the brush-turkey clade probably had its origins in Australia–New Guinea, while the scrubfowl clade probably evolved in Wallacea and thence dispersed widely through Oceania.

The scrubfowl and brush-turkey clades were estimated in that study to have diverged around 18 Ma during the early Miocene [4]. However, a recent large-scale molecular phylogeny of birds, which estimated divergence dates of modern avian groups using clock-like genes, estimated that the branches leading to extant *Leipoa* and *Megapodius* (and by inference, the brush-turkey and scrubfowl clades) diverged at greater than 25 Ma during the Oligocene [8], some 7 Myr earlier than the prior estimate [4]. The oldest known fossil megapode, the tiny *Ngawupodius minya* from Lake Pinpa in central Australia, is of Late Oligocene age (approx. 26–24 Ma) [9], and could therefore potentially be a crown group megapode belonging either to the brush-turkey or scrubfowl clade if an Oligocene divergence date is correct. However, remains of this taxon are sparse, and its phylogenetic affinities are so far unknown.

There is currently no pre-Late Oligocene fossil record for the Megapodiidae, despite recent estimates for the family's divergence from other galliforms ranging from approximately 45 Ma in the Eocene [10] to as much as 70–75 Ma in the Late Cretaceous [4]. The more recent fossil record of megapodes is also sparse, with a 20 million-year gap in the record between *N. minya* and the next appearance of megapodes in the Pliocene. A few bones representing one or more species have been described from the Pliocene deposits of Bluff Downs [11] and the Chinchilla Sand [12] in Queensland. These are discussed below (see Systematic palaeontology).

The richest megapode fossil record is of Quaternary age. A few fragmentary bones of a species of *Aepypodius*, possibly *Aepypodius arfakianus*, are known from a site of possible Late Pleistocene age on Irian Jaya [13]. Four extinct Holocene taxa have been described from Islands in the Pacific: *Megapodius molistructor* is known from New Caledonia and Tonga [14,15]; *Megapodius alimentum* from Tonga and Fiji [15,16]; *Megapodius amissus* from Fiji [16] and *Mwalau walterlinii* from Vanuatu [17]. A large, undescribed species of *Megapodius* is also known from New Ireland off eastern New Guinea [6,18].

Two further extinct Holocene taxa from the Pacific that were previously thought to have been megapodes, *Sylviornis neocaledoniae* from New Caledonia [19] and *Megavitiornis altirostris* from Fiji [16], have since been referred to a separate family, the Sylviornithidae, which is now regarded as the sister group to all extant galliforms [20,21]. This study considers the increasingly rich fossil megapode record from the Plio-Pleistocene of Australia. ‘Giant’ extinct taxa in the genus *Progura* have been described from southeast Queensland, eastern New South Wales and southeastern South Australia [22–24], but as we outline in greater detail below, the number of genera and species represented among these remains is controversial and requires clarification.

### 1.1. Australia's megapodes and their late Cenozoic fossil record

Australia has three extant species of megapode: the endemic *Leipoa ocellata* and *Alectura lathami*, and *Megapodius reinwardt*, which is shared with Indonesia and New Guinea [5]. The malleefowl *L. ocellata*, the only extant megapode adapted to arid environments, is found only in southern Australia [3]. The Australian brush-turkey *Al. lathami* has two subspecies: *Al. lathami lathami* in high-rainfall eastern Australia in tropical, subtropical and temperate zones; and *A. l. purpureicollis* on Cape York Peninsula in far north Queensland [5]. Although *M. reinwardt* is not endemic to Australia, three of its five recognized subspecies are. *Megapodius reinwardt tumulus* is found in northwestern and central northern Australia, *M. r. yorki* on the Cape York Peninsula and *M. r. castanonotus* in coastal northeastern Queensland [5]. A fossil record for all these taxa is essentially lacking. Megapodes, either extant or fossil, are not known from Tasmania.

**Figure 2.**
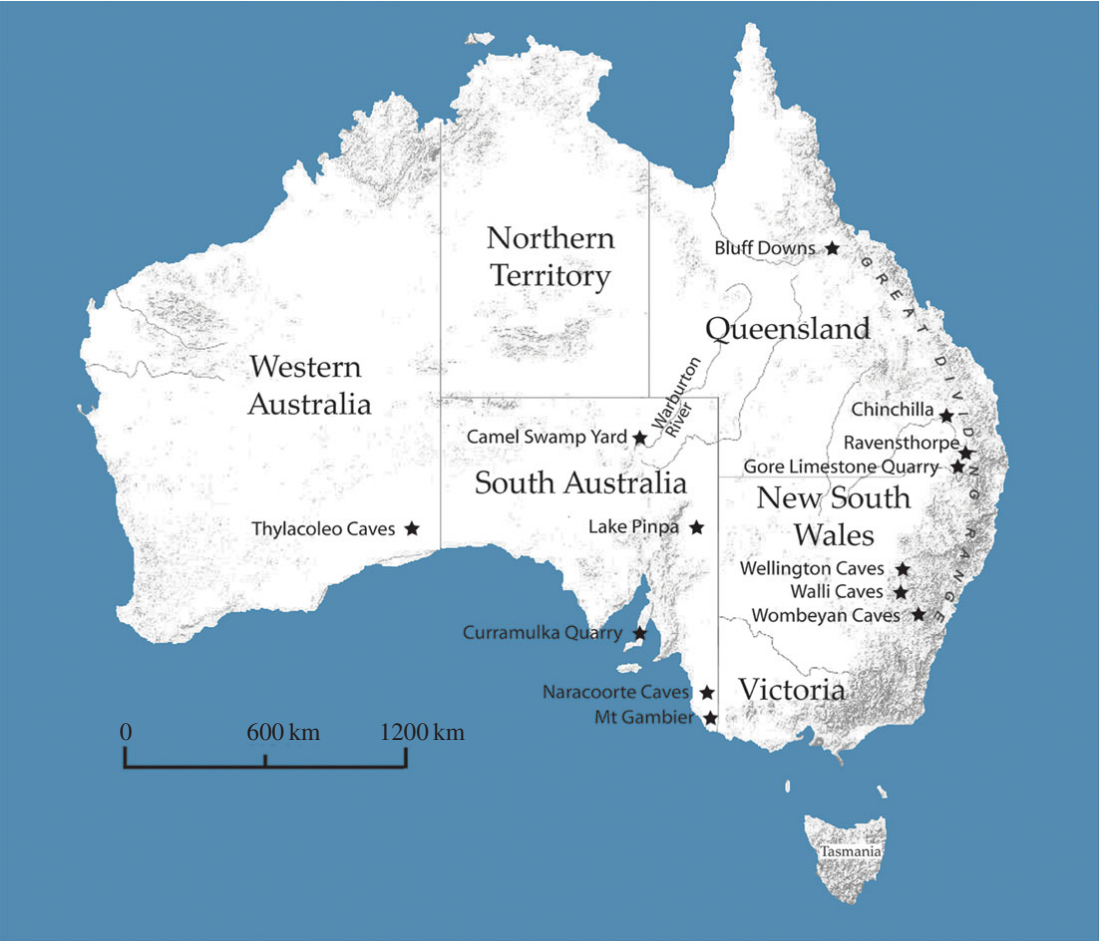
Key Australian fossil megapode localities.

The earliest-described extinct megapode, the very large species *Progura gallinacea*, was described from deposits near the Condamine River in southeastern Queensland [22] (figure 2). The genus name was derived from De Vis' mistaken belief that the tarsometatarsi in the type series could be referred to a crowned pigeon ancestral to the *Goura* pigeons of New Guinea. *Progura gallinacea*, including several additional fossils from Queensland that De Vis erroneously referred to other avian families, was later placed in the Megapodiidae [23], along with more recently discovered Pleistocene remains from Walli Caves, Wellington Caves and Wombeyan Quarry in eastern New South Wales (figure 2). In the same paper, van Tets described a second, somewhat smaller species, *P. naracoortensis*, from Pleistocene cave deposits in the Naracoorte region of southeastern South Australia, and from Gore Limestone Quarry in southeastern Queensland (figure 2). However, he later informally revised his taxonomic decision, suggesting that the larger individuals were males and the smaller ones females of a single, sexually dimorphic species, *P. gallinacea* [25]. Conflicting with this, Olson [26] commented that a cursory examination of fossils of *P. gallinacea* and *P. naracoortensis* led him to believe that they belonged in separate genera, but did not elaborate on why.

The identity of the two species of *Progura* was formally investigated by Boles [24], who agreed with van Tets that fossils attributed to *P. gallinacea* and *P. naracoortensis* belonged to one species. He concluded that apparent proportional differences within the skeleton between the two nominal species—namely the ratio of tarsometatarsus length to coracoid length given by van Tets [23]—were not valid due to the lack of associated skeletons. Boles [24] argued that the coefficients of variation for lengths of tarsometatarsi and coracoids from modern specimens of *Al. lathami* and *L. ocellata* were ‘not dissimilar to those of the *Progura* specimens collectively’, and this was the primary reason given for synonymizing *P. naracoortensis* with *P. gallinacea.* However, Boles' calculations of the mean tarsometatarsus length excluded the type material of *P. gallinacea* from the Darling Downs, and were based only on tarsometatarsi of *P. naracoortensis* from Naracoorte. The study also lacked a morphological comparison of the type tarsometatarsi of the two nominal species. In our opinion, differences in the morphology and size of specimens referred to *P. gallinacea* and *P. naracoortensis sensu* van Tets [23] have not been satisfactorily addressed to date, and we aim to rectify this in our study.

A further matter to be resolved is the relationship between the large extinct megapodes and extant *L. ocellata*. Boles [24] proposed that *P. gallinacea* was a giant chronospecies of *L. ocellata*, the small size of the latter being the result of ‘Late Pleistocene dwarfing’, as purportedly observed in some other taxa [27]. Noting no substantial differences in hind limb morphology between *Progura* and *Leipoa* besides size, Boles suggested that the large Pleistocene species should be referred to *Leipoa gallinacea.* The nomen *L. gallinacea* has subsequently been adopted in the literature for large Pleistocene megapode fossils [12,28,29]. However, the dwarfing theory requires re-examination, in part because of the problematic synonymy of *P. gallinacea* and *P. naracoortensis*, but also because small Pleistocene fossils referred to *L. ocellata*, and large fossils referred to *P. naracoortensis*, have both been collected from the Main Fossil Chamber of Victoria Fossil Cave, Naracoorte [30], casting doubt on the chronospecies hypothesis.

The recent discovery of both large and small megapode fossils from Pleistocene deposits of the Thylacoleo Caves of the Nullarbor Plain, Western Australia, has prompted our re-examination of the Late Cenozoic megapode fossils from Queensland, New South Wales and southeastern South Australia, and the first examination of previously unstudied megapode fossils from Curramulka and the Warburton River, South Australia (figure 2). We address the generic and species diversity of Australia's Plio-Pleistocene megapodes, as well as the evolutionary relationships between the extinct taxa and extant megapodes.

## 2. Material and methods

### 2.1. Abbreviations and definitions

**Institutions:** AM, Australian Museum, Sydney; AMNH, American Museum of Natural History, New York; ANWC, Australian National Wildlife Collection, Canberra; FU, Flinders University, Adelaide; KU, University of Kansas Natural History Museum, Lawrence; USNM, National Museum of Natural History, Washington, DC; NHMUK, Natural History Museum, London; NMNZ, Museum of New Zealand Te Papa Tongarewa, Wellington; NMV, Museum Victoria, Melbourne; QM, Queensland Museum, Brisbane; SAM, South Australian Museum, Adelaide; WAM, Western Australian Museum, Perth.

**Geological timescale:** Pliocene = 5.3–2.58 Ma; late Pliocene (Piacenzian) = 3.6–2.58 Ma; Early Pleistocene = 2.58–0.78 Ma; Middle Pleistocene** **= 780–126 thousand years ago (ka); Late Pleistocene** **= 126–11.7 ka; Quaternary = 2.58–0 Ma; Holocene = 11.7–0 ka; Naracoortean = biocorrelated Australian land mammal age of Megirian *et al.* [31], spanning max. 3.03–2.58 Ma to present; Tirarian = biocorrelated Australian land mammal age of Megirian *et al.* [31], spanning 4.46–3.6 Ma.

**Other terminology:** CV, coefficient of variation; dL, distal left; dR, distal right; L, left; Ma, million years ago; m., musculo (i.e. muscle, Latin); mm, millimetres; OSL, optically stimulated luminescence; pL, proximal left; pR, proximal right; R, right; s.d., standard deviation; troch., trochlea/e; year BP, years before present.

### 2.2. Comparative material

Modern skeletons of megapode taxa were examined as follows. Malleefowl *L. ocellata*: SAM B.414, SAM B.1094, SAM B.5039, SAM B.11482, SAM B.55458, SAM B.11480, SAM B.11481, SAM B.47825, SAM B.48526, SAM B.48765, SAM B.49461, SAM B.51215, SAM B.55528, SAM B.58520, SAM B.58560; Australian Brush-turkey *Al. lathami*: SAM B.46568, QM O.27218, QM O.27843, QM O.27844, QM O.27852, NMV B.2209, NMV B.4288, NMV B.11471, NMV B.19290, NMV B.23648, NMV B.23649, NMV B.23650; Moluccan Megapode *Eulipoa wallacei*, USNM 558275; Orange-footed Scrubfowl *M. reinwardt*: ANWC O.22869; Melanesian Megapode *Megapodius eremita*: NMV B.20648, NMV B.20641, NMV B.20642, NMV B.20647, NMV B.24000, NMV B.24947, NMV B.24948, NMV B.24949, NMV B.24950, NMV B.24951, NMV B.24952, NMV B.25389; Black-billed Brush-turkey *Talegalla fuscirostris*: ANWC 03669, KU 97007; Collared Brush-turkey *Talegalla jobiensis*, ANWC 07567, USNM 146744; Wattled Brush-turkey *Ae. arfakianus*: ANWC O.26042; Waigeo Brush-turkey *Aepypodius bruijnii*, USNM 146767; Maleo *Macrocephalon maleo*, AMNH 12013 (by photographs taken 2000 by J. Palmer), NHMUK 1891.7.20.97, 1871.7.21.1, USNM 225130.

### 2.3. Key locations

Key fossil-bearing localities mentioned in the Systematic palaeontology section are shown in figure 2. Brief accounts of the localities are as follows.

#### 2.3.1. Darling Downs, southeastern Queensland

Megapode fossils have been recovered from three locations on the Darling Downs: Chinchilla, Ravensthorpe and Gore Limestone Quarry [23]. The Chinchilla Sand has not been directly dated, but its mammal fauna is dated to be of Pliocene age by biocorrelation with the Kanunka and Toolapinna Local Faunas of the Tirari Formation, Lake Eyre Basin, to be approximately 3.6 million years old [12], and thus fossils from the locality fall within the Tirarian land mammal age [31]. Ravensthorpe, near Pilton, in the Clifton region east of King's Creek on the Eastern Darling Downs [32] is within a river catchment where various fossil-bearing sites have been dated to the Late Pleistocene [33]. Fossils from Ravensthorpe are presumed to also be of Pleistocene age, and therefore within the Naracoortean land mammal age [31]. Fossils from Gore Limestone Quarry are mainly from fissure fills, and are considered to be mainly Pleistocene in age [34].

#### 2.3.2. Warburton River, northeastern South Australia

A single megapode fossil has been collected from CAM 4 Quarry, Camel Swamp Yard (27°44.021′ S, 137°45.196′ E) [35], Warburton River, South Australia (figure 2). This site contains a late Pliocene fossil fauna belonging to the Toolapinna Local Fauna within the Tirari Formation [35,36].

#### 2.3.3. Thylacoleo Caves, Nullarbor Plain, Western Australia

A species of megapode is recorded in the faunal list for the Thylacoleo Caves [28], Nullarbor Plain, Western Australia (figure 2). The three caves comprising this locality (Leaena's Breath Cave, Last Tree Cave and Flightstar Cave) are formed within the early Miocene-aged Nullarbor Limestone and preserve a vertebrate fossil fauna of Early and Middle Pleistocene age [28], falling within the Naracoortean land mammal age [31]. Precise locations of the caves are registered with the Department of Earth and Planetary Sciences, Western Australian Museum, Perth. The fossil fauna is presumed to have accumulated via pitfall trapping through the solution pipe entrances to the caves, during intervals in the Pleistocene when they were open to the surface.

#### 2.3.4. Naracoorte, southeastern South Australia

The majority of megapode fossils recorded to date have been collected from various caves in the Naracoorte region (figure 2). Some of the caves comprise the Naracoorte Caves World Heritage Area [30], while others are in the surrounding area. Caves in the region preserve Middle Pleistocene, Late Pleistocene and Holocene fossils [29,30]. The majority of fossil megapode material comes from Henschke's Fossil Cave. This cave, which is formed within the Miocene-aged Gambier Limestone, was discovered within a working quarry and was eventually destroyed in 1981 following extensive excavation during the previous decade [37]. It preserved a Pleistocene fauna, probably Middle or Late Pleistocene in age [37], falling within the Naracoortean land mammal age [31].

#### 2.3.5. Curramulka Quarry, Yorke Peninsula, South Australia

Curramulka Quarry (site RF 95) is a limestone quarry near the township of Curramulka (34°42′11.8^″^ S, 137°42′14.3^″^ E), on the Yorke Peninsula, South Australia. The RF95 fissure-fill is considered to have accumulated vertebrate remains during the Pleistocene based on the presence of macropodid species that occur in deposits of this age elsewhere [38–40].

### 2.4. Measurements

Measurements were taken with digital callipers and rounded to the nearest 0.1 mm. Long-bone circumferences used for body mass calculations were obtained by wrapping a thin strip of paper wrapped around the shaft, marking with a pen where the ends overlapped, and then straightening out the paper and measuring the distance between the marks with digital callipers. Measurements were made only on skeletally mature bones, identified by their smooth, non-porous surface and well-defined epiphyses.

### 2.5. Nomenclature

We follow the osteological terminology of Baumel *et al.* [41] unless otherwise specified, and the taxonomic nomenclature of Dickinson & Remsen [5] for extant taxa. Nomenclature of extinct taxa is addressed in the Systematic palaeontology section.

### 2.6. Body mass estimates

Where possible, body mass of extinct megapodes was estimated using regression equations based on minimum shaft circumferences of the femur and tibiotarsus [42]. We selected equations from the functional category of ‘heavy-bodied birds’, based on measurements of birds from 11 families, including some galliforms [42]. Where fossil femora or tibiotarsi were missing or too damaged to measure, we substituted an equation using the minimum width of the tarsometatarsus [43]. We elected not to use Field *et al*.'s [43] preferred equation, based on the maximum length of the humeral facet on the coracoid, because this facet has indistinct boundaries in megapodes, which would introduce measurement error.

### 2.7. Simpson log-ratio diagrams

We compared the body proportions of megapode species using the log-ratio method first described by Simpson [44], and now used widely. In this study, we used the domestic chicken *Gallus gallus* as the arbitrary comparator. Relative size of different species is shown by their height on the *y*-axis.

### 2.8. Phylogenetic analysis

To test the validity of the extinct genera and species we identified via our morphological examinations, and to examine their relationships to extant megapode taxa, we scored them into a 285-character matrix for galloanseres, updated from Worthy *et al.* [21]. This matrix has 283 osteological characters, one behavioural character and one non-osteological character of the foot, with palaeognaths and three species of Neoaves used as the outgroup. We undertook parsimony analyses in PAUP* 4.0b10 using standard settings [45], heuristic searches, tree bisection-reconnection branch swapping and 1000 random addition replicates per search. Following Worthy *et al*. [21], relationships between extant taxa were constrained using a backbone based on recent molecular data, but those of megapodes were altered to reflect the most recent phylogeny of the Megapodiidae [4] (figure 1). Support for the consensus tree was assessed in PAUP* via bootstrapping, using heuristic searches and the same options, and 1000 replications. Several non-Australian fossil taxa that were included in the consensus tree were excluded from the final bootstrap analysis because they were scored from incomplete fossil remains, and the resulting uncertainty led to reduced tree resolution and support. Trees were manipulated in FigTree v. 1.4.2 and labelled in Adobe Illustrator.

## 3. Results

### 3.1. Systematic palaeontology

#### **Galliformes** Temminck, 1820

##### **Megapodiidae** Lesson, 1831

The fossil specimens described below are referred to Galliformes and therein to Megapodiidae based mainly on features noted by Mourer-Chauviré [46], Worthy *et al*. [17,21] and Mayr & Weidig [47].

Humerus: the crista bicipitalis is more elongate and projects less ventrally than in Phasianidae; the dorsal fossa pneumotricipitalis is shallow, unlike in the stem-galliform *Gallinuloides* [47]; the impression for the insertion of m. coracobrachialis caudalis is dorsad of the incisura capitis, indents the crista incisura capitis distalis and abuts the midpoint of the caput humeri; the impression for the insertion of m. coracobrachialis caudalis is bound dorsally by a small tuberculum intermedium, rather than by a strongly marked tuberculum; the caudal surface of the shaft is compressed into a distinct ridge (capital shaft ridge) level with the distal side of the crista bicipitalis; and the attachment of the m. latissimus dorsi is located dorsad of the margo caudalis (ventrally in all other galliforms except for the Sylviornithidae [21]).

Carpometacarpus: the facies articularis scapularis is flat to slightly convex, lacking the cup-like, concave facet seen in some stem-galliforms from the Northern Hemisphere [47].

Tarsometatarsus: the eminentia intercotylaris is low and rounded, and barely projects further proximally than the area intercotylaris; the medial margin adjacent to the sulcus extensorius forms a sharp crest; the distal half of the facies dorsalis is convex, rather than flat or concave; the fossa metatarsi I is large and deep, with the rim of its articular facet projecting medially of the shaft margin. The hypotarsus has a single enclosed canal for m. flexor digitorum longus [48].

##### ***Progura*** De Vis, 1888

***Progura*** De Vis, 1888—type species *P. gallinacea* De Vis, 1888 by monotypy.

***Chosornis*** De Vis, 1889: *Proceedings of the Royal Society of Queensland* 6: 55, Pl. IV—type *Chosornis praeteritus* De Vis, 1889 by monotypy, see van Tets (1974), *Transactions of the Royal Society of South Australia*, 98, p. 214.
***Palaeopelargus*** De Vis, 1892: *Proceedings of the Linnean Society of New South Wales* (Ser. 2) 6: 441, Pl. XXIV—type *Palaeopelargus nobilis* De Vis, 1892 by monotypy, see van Tets [23], *Transactions of the Royal Society of South Australia*, 98, p. 214.

**Included taxa:** *Progura gallinacea* De Vis, 1888; *Progura campestris* sp. nov. (see below)

**Revised diagnosis:** *Progura* is distinguished from all other megapode genera by the following unique combination of features of the tarsometatarsus.

(i) The shaft is elongate, the proximal and distal ends are proportionally narrow relative to length (PW = approx. 19–22.8% length; DW = approx. 20–22.6% length). (ii) The shaft does not flare proximomedially into a convex profile in dorsal view as it widens to meet the cotyla medialis. (iii) Dorsally, the lateral and medial foramina vascularia proximalia are of similar size and are about equidistant from the proximal end of the bone. (iv) The sulcus infracotylaris dorsalis is a shallow depression bound by slightly raised areas of bone laterally and medially but not proximally. (v) The tuberositas m. tibialis cranialis comprises two short, broad tuberosities of about equal size, which are positioned symmetrically with respect to the midline of the shaft, are equidistant from the proximal end of the bone, diverge proximally, are separated from the foramina vascularia proximalia by a distinct gap and are recessed in the sulcus extensorius, thus do not protrude above the dorsal facies in lateral or medial aspect. (vi) The impressiones retinaculi extensorii are low crests, unlike all other megapode genera, and both retinaculi, but especially the medial one, are located proximal of the level of the foramina vascularia proximalia. (vii) The hypotarsus is dorsoplantarly shallow (proximal part of the medial hypotarsal ridge is approx. 40% of the depth of the medial cotyla). (viii) The hypotarsus is slightly recurved distally into a hook in lateral/medial aspects, and the junction between the distal part of the medial hypotarsal crest and the plantar facies is gradual*.* (ix) Trochleae metatarsi II and IV are weakly grooved dorsally.

**Differential diagnosis:** The tarsometatarsi of extant genera of megapode differ from *Progura* as follows. (i) In *Leipoa,* the shaft is proportionally shorter and stouter, the distal end is proportionally wider (*Leipoa* DW = 23–24% length. (ii) In *Leipoa* and *Megapodius*, the proximal end flares strongly medially from the shaft as it widens to meet the cotyla medialis, thus housing a larger fossa parahypotarsalis medialis, whereas the proximal end is more symmetrical, and the fossa smaller, in *Progura*. (iii) In *Leipoa*, *Alectura*, *Megapodius* and *Eulipoa*, the foramen vascularis proximalis medialis is larger and placed a little more distally than its lateral counterpart (relative size and position of the foramina could not be accurately determined in *Aepypodius* due to immaturity of the available specimen; character state is variable in *Talegalla*). (iv) In *Leipoa*, *Alectura* and especially so in *Megapodius*, the sulcus infracotylaris is deeper, and is bounded by raised areas of bone laterally, medially and proximally (could not be accurately determined in *Aepypodius*; variable among species of *Talegalla*). (v) *Leipoa* is differentiated by having a single fused tuberositas m. tibialis cranialis rather than the tuberositas having two distinct parts. In *Talegalla*, this character is variable among species. All other genera have an elongated tuberosity visibly divided into two parallel ridges (could not be determined in *Aepypodius*), and further differ from *Progura* by their placement and relative size: in *Megapodius*, *Eulipoa* and *Alectura*, the medial part of the tuberosity is broader and more elevated from the shaft than the lateral part; in *T. jobiensis*, the tuberosities are particularly elongate and are laterally offset from the midline of the bone shaft. In *Megapodius*, *Eulipoa* and *Leipoa*, the tuberositas is dorsally prominent and is visibly elevated above the shaft surface in lateral and/or medial aspect (character is variable among species of *Talegalla*). (vi) In all compared genera, the impressiones retinaculi extensori are more prominent than in *Progura*, and are especially prominent in *Macrocephalon*. The placement of the retinaculi differs from *Progura* in other genera as follows: in *Megapodius*, the proximodistal placement of both retinaculi is roughly level with the foramen vascularis proximalis lateralis, with the lateral retinaculum abutting the sulcus infracotylaris; in *Alectura*, both retinaculi are about level with one another and are placed slightly proximal of the level of the foramina; in *Leipoa*, the medial retinaculum is placed further proximally than the lateral, with the lateral retinaculum placed immediately proximomedial to the foramen proximalis medialis, abutting the sulcus infracotylaris dorsalis; proximodistal placement of the retinaculi is variable in *Talegalla*, these being roughly level with the foramina in *T. fuscirostris* and proximal of the level of the foramina in *T. jobiensis.* (vii) The hypotarsus is proportionally deeper dorsoventrally (approx. 50% of the depth of the cotyla medialis) in *Leipoa* and *Megapodius*. (viii) In *Megapodius* and *Eulipoa*, the hypotarsus is strongly recurved distally, and has a deeply hooked appearance in lateral/medial aspect. The hypotarsus in *Talegalla* and *Alectura* is also hooked, although less strongly so. In *Megapodius* and *Aepypodius*, the junction between the distal end of the medial hypotarsal crest and the plantar facies is abrupt rather than smoothly curved, meeting at approximately 90° in medial aspect. (ix) Trochlea metatarsi II lacks a median groove distodorsally in *Leipoa*, *Macrocephalon*, *Megapodius*, *Eulipoa*, *Aepypodius*, *Alectura* and *Talegalla*. Trochlea IV is strongly grooved distodorsally in *Leipoa*, *Macrocephalon*, *Megapodius*, *Alectura* and weakly so in *Aepypodius* and *Talegalla*.

**Remarks:** Our morphological observations indicate substantial differences between *Progura* and *Leipoa*. As per our generic diagnosis, compared with *Leipoa*, *Progura* has: a more elongate tarsometatarsus, lacking a marked medial flaring of the shaft proximally; similar-sized vascularia proximalia; a relatively shallow sulcus infracotylaris dorsalis; the tuberositas m. tibialis is paired and unfused rather than comprising a single fused tuberosity; weakly marked and more proximally located impressiones retinaculi extensorii; less plantar extension of the hypotarsus; a trochlea metatarsi II that lacks a median groove; and a trochlea metatarsi IV that is more weakly grooved. Therefore, we reject the statement that ‘[o]ther than size, no differences could be found between these two genera that could not be attributed to individual variation within and among the samples’ [24]. These, and multiple other differences in other parts of the skeleton (see below), lead to our rejection of the synonymy of *Progura* with *Leipoa* (cf. [24]). This conclusion receives further support in our phylogenetic analysis (see below).

**Geological range:** Late Pliocene [12]; Pleistocene ([41]; data herein).

**Geographical range:** The known geographical range of *Progura* includes southeastern Queensland, northeastern South Australia and the Nullarbor Plain, Western Australia (see species accounts below).

##### ***Progura gallinacea*** De Vis, 1888

##### (figures 3–5*a*,*f*,*k*,*p*,*u*)

***Progura gallinacea* De Vis, 1888**: *Proceedings of the Royal Society of Queensland* 5: 131, Pl. VI—Ravensthorpe, near Pilton, Clifton region east of King's Creek, Eastern Darling Downs, southeastern Queensland, Australia [37]; **Lectotype:** QM F1143, a pL tarsometatarsus [24]; **Paralectotypes:** QM F1134, a pL tarsometatarsus; QM F5556, a dR tarsometatarsus; QM F5557, a dR tarsometatarsus [24].

***Chosornis praeteritus*** De Vis, 1889: *Proceedings of the Royal Society of Queensland*, 6: 55, Pl. IV—Chinchilla, Darling Downs, Queensland, Australia; late Pliocene or Pleistocene; see van Tets [23], p. 214.
***Palaeopelargus nobilis*** De Vis, 1892: *Proceedings of the Linnean Society of New South Wales* (Ser. 2) 6: 441, Pl. XXIV—Chinchilla, Darling Downs, Queensland, Australia; late Pliocene or Pleistocene; see van Tets [23], pp. 214, 224.
***L.*** [***eipoa***] ***(Progura) gallinacea*** (De Vis, 1888): Boles (2008), *Oryctos* 7: 204, in part.
***Leipoa gallinacea*** (De Vis, 1888): Louys & Price (2015), *Acta Palaeontologica Polonica* 60 [3]: 557.
***Leipoa gallinacean*** (De Vis, 1888): Louys & Price (2015), *Acta Palaeontologica Polonica* 60 [3]: 559, figure 5; unjustified emendation.

**Figure 3.**
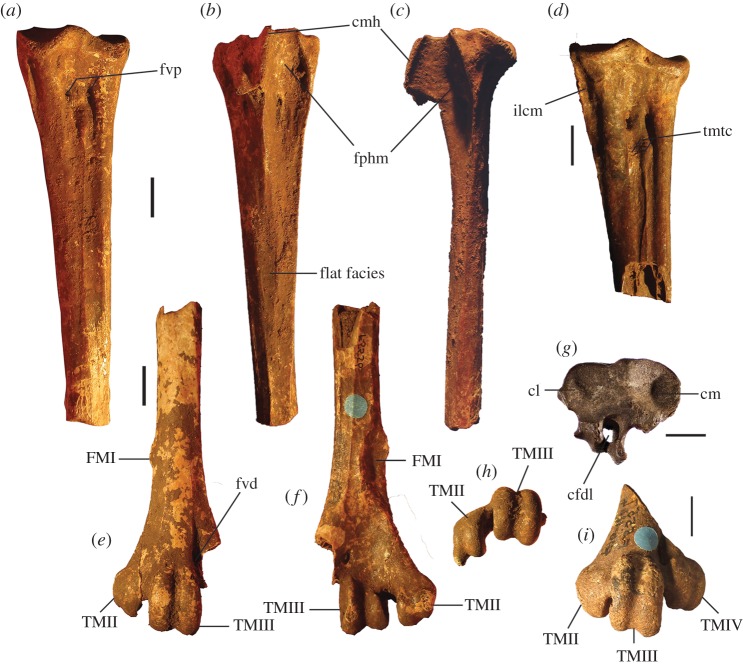
Tarsometatarsi of *P. gallinacea* De Vis, 1888. Lectotype (QM F1143, left) in dorsal (*a*), plantar (*b*) and medial (*c*) aspects; paralectotype (QM F1134, left) in dorsal (*d*) and proximal (*g*) aspects; paralectotype (QM F5556, right, images reversed) in dorsal (*e*), plantar (*f*) and distal (*h*) aspects; paralectotype (QM F5557, right, image reversed) in dorsal aspect (*i*). Scale bars, 10 mm. cfdl, canal for m. flexor digitorum longus; cl, cotyla lateralis; cm, cotyla medialis; cmh, crista medialis hypotarsi; FMI, fossa metatarsi I; fphm, fossa parahypotarsalis medialis; fvd, foramen vasculare distale; fvp, foramina vascularia proximalia; ilcm, impressio lig. collateralis medialis; TMII, trochlea metatarsi II; TMIII, trochlea metatarsi III; TMIV, trochlea metatarsi IV; tmtc, tuberositas m. tibialis cranialis.

**Figure 4.**
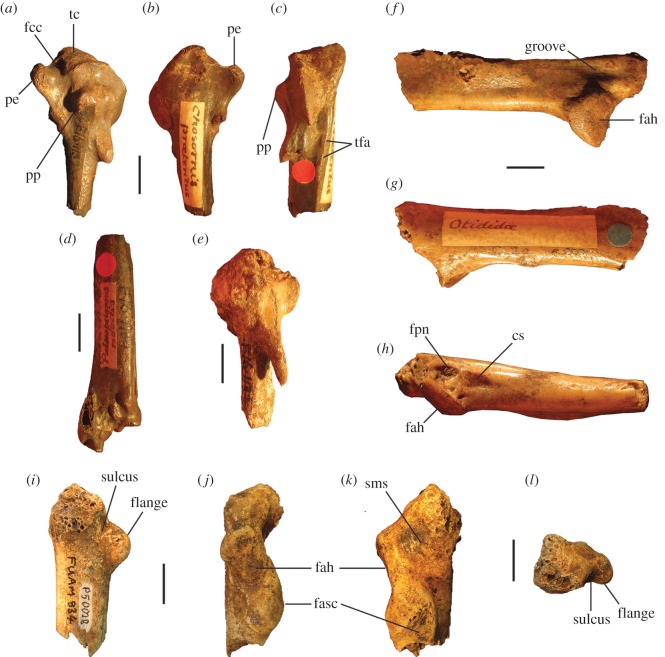
Pectoral elements of *P. gallinacea* De Vis, 1888. Carpometacarpus, QM F1132, holotype of *C. praeteritus* De Vis, 1889 (joins to QM F1139) in ventral (*a*), dorsal (*b*) and caudal (*c*) aspects; carpometacarpus, QM F1139, holotype of *Pa. nobilis* De Vis, 1891 (joins to QM F1132) in caudal (*d*) aspect; carpometacarpus, QM F7005, in ventral (*e*) aspect; scapula, QM F5558, in lateral (*f*), medial (*g*) and ventral (*h*) aspects; coracoid, SAM P50028, in ventral (*i*), lateral (*j*), dorsal (*k*) and omal (*l*) aspects. cs, collum scapulae; fah, facies articularis humeralis; fasc, facies articularis scapularis; fcc, fovea carpalis cranialis; fpn, foramen pneumaticum; pe, processus extensorius; pp, processus pisiformis; sms, sulcus m. supracoracoidei; tc, trochlea carpalis; tfa, tuberosity for flexor attachment. Scale bars, 10 mm.

**Figure 5.**
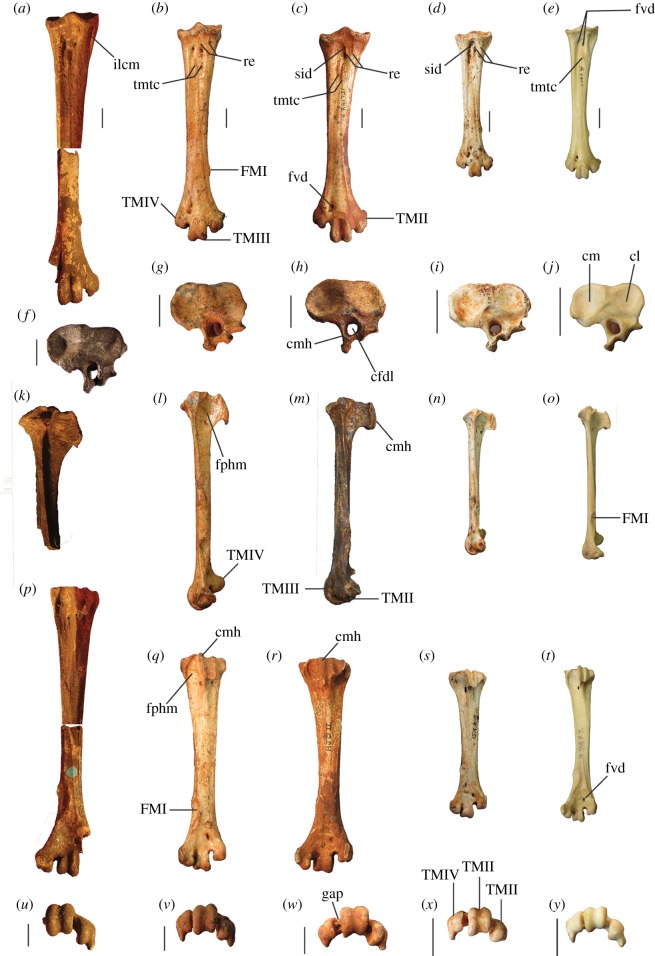
Tarsometatarsi of extinct Australian megapodes compared with extant malleefowl *L. ocellata*. *Progura gallinacea* (*a*,*f*,*k*,*p*,*u*); *P. campestris* sp. nov. WAM 15.9.5, holotype (*b*,*g*,*l*,*q*,*v*); *La. naracoortensis* (*c*,*r*,*w* = SAM P41731; *h*,*m* = SAM P51233, left, image reversed); *La. olsoni* sp. nov. WAM 15.9.6, holotype (*d*,*i*,*n*,*s*,*x*); *L. ocellata* SAM B.11482 (*e*,*j*,*o*,*t*,*y*). Bones in dorsal (top row), proximal (second row), medial (third row), plantar (fourth row) and distal (bottom row) aspects. (*a*) and (*p*) are reconstructed from photographs of two incomplete bones (proximal end = QM F1143, left, image reversed; distal end = QM F5556). *f*,*k* = QM F1134, left, image reversed. *u* = QM F5556, right. cfdl, canal for m. flexor digitorum longus; cl, cotyla lateralis; cm, cotyla medialis; cmh, crista medialis hypotarsi; FMI, fossa metatarsi I; fphm, fossa parahypotarsalis medialis; fvd, foramen vasculare distale; fvp, foramina vascularia proximalia; ilcm, impressio lig. collateralis medialis; re, impressiones retinaculi extensorii; sid, sulcus infracotylaris dorsalis; tmtc, tuberositas m. tibialis cranialis; TMII, trochlea metatarsi II; TMIII, trochlea metatarsi III; TMIV, trochlea metatarsi IV. Scale bars, 10 mm.

**Referred material: QM F1132**, pR carpometacarpus, Holotype *C. praeteritus* De Vis, 1889, joins to QM F1139 (Chinchilla, Darling Downs, Queensland); **QM F1139**, dR carpometacarpus, Holotype *Pa. nobilis* De Vis, 1892: joins to QM F1132, (Chinchilla, Darling Downs, Queensland); **QM F5553**, dR ulna (unknown locality, Darling Downs, Queensland); **QM F5558**, pR scapula (Chinchilla, Darling Downs, Queensland); **QM F7005**, pR carpometacarpus (unknown locality, Darling Downs, Queensland); **SAM P50028**, L coracoid, omal fragment (Warburton River, northeastern South Australia).

**Type locality:** The lectotype and paralectotypes are from Ravensthorpe, Eastern Darling Downs, Queensland (see Key locations; figure 2).

**Stratigraphy, age and fauna:** Stratigraphic information for the lectotype and paralectotypes was not recorded when the material was collected from Ravensthorpe in the late nineteenth century, but the specimens share similar preservation, having been stained a uniform dark brown consistent with their being from one location, and are considered Pleistocene in age (see Key locations). Referred specimens from Chinchilla, Darling Downs, are of late Pliocene age if they arise from the Chinchilla Sand (see Key locations). The partial coracoid was collected from CAM 4 Quarry, Camel Swamp Yard, Warburton River, South Australia, and is of late Pliocene age [40].

**Revised diagnosis:** A species of *Progura* with diagnostic features of the tarsometatarsus as for the genus, and distinguished from all other species of megapode, including the extinct ‘*Progura*’ *naracoortensis,* which is transferred to a new genus below, by its very large size and the following unique combination of morphological features.

(i) The shaft tapers evenly down its entire length, and is narrowest immediately proximal to fossa metatarsi I. (ii) The medial edge of the shaft is dorsoplantarly compressed into a thin crest that is offset plantarly from the dorsal facies, and separated from the sulcus extensorius and foramina vascularia by a shallow sulcus on the dorsal facies, thus giving the shaft a somewhat twisted appearance in dorsal aspect. (iii) The fossa parahypotarsalis medialis extends to about half the shaft length. (iv) The impressio lig. collateralis medialis forms a very deep depression on the proximomedial surface just distal of the medial cotyla. (v) The plantar surface of the midshaft is flattened. (vi) The facet for metatarsal I is proportionally wide, extending to the midline of the shaft. (vii) And in dorsal aspect, the rims of trochlea metatarsi III are parallel.

**Description and comparisons:** Only a few elements of this species are known (figures 3 and 4), and their morphology has not previously been described in detail. All are larger than in any other megapodid species. Some specimens previously referred to *P. gallinacea* [23,24] are either not megapodes, or are megapodes that do not belong to this taxon. These are noted below following the descriptions.

**Ulna:** A portion of distal ulna approximately 50 mm long (QM F5553) from an unknown locality on the Darling Downs, southeast Queensland, was originally referred to *Pa. nobilis* by De Vis, and was referred to *P. gallinacea* by van Tets [23]. It is significantly eroded and is not sufficiently well preserved to make a detailed morphological description. We concur that it belongs to a very large megapode, but refer this bone to *P. gallinacea* only tentatively, based on its very large size and the fact that it was collected by De Vis from the Darling Downs in the 1880s or 1890s, as with other material of this species.

**Carpometacarpus:** Two carpometacarpi have previously been referred to *P. gallinacea*, under three catalogue numbers (see Referred material). A proximal specimen (QM F7005) is badly degraded (figure 4*e*) and we refer this only tentatively. The other two bone fragments, QM F1132, a proximal right carpometacarpus (figure 4*a–c*), which is the holotype for *C. praeteritus* De Vis, 1889, and QM F1139, a distal right carpometacarpus (figure 4*d*), which is the holotype of *Pa. nobilis* De Vis, 1892, join as a single bone, and so both nomina are synonyms of *P. gallinacea* [23]. The os metacarpale minus of this specimen is not preserved, but the two halves are in otherwise good condition and show that the carpometacarpus of this species is differentiated from those of all extant megapodes by its very much larger size. Total combined length of QM F1132 and QM F1139 is approximately 103 mm, longer than the carpometacarpi of all other extinct species of megapode (see species accounts below). Proximal and distal widths (table 1) also exceed the size range of the other extinct species. The carpometacarpus has features as follows. The fovea carpalis cranialis is deep, as in *Al. lathami* and *L. ocellata*. The processus extensorius is cranially orientated. The ventral rim of the trochlea carpalis does not project much proximally past the tip of the processus extensorius, and is smoothly curved caudally. In dorsal aspect, the dorsal rim of the trochlea projects strongly proximally, and its caudal and cranial margins meet proximally at an approximately 90° angle. In caudal aspect, the ventral rim of the trochlea carpalis is orientated obliquely relative to the long axis of the bone, and thus converges distally with the dorsal rim of the trochlea at a point dorsal to the os metacarpale minus, as in *Al. lathami* and *L. ocellata*. In proximal aspect, the processus extensorius is dorsoventrally thick (more than half the thickness of the adjacent carpal trochlea, whereas in *Al. lathami* and *L. ocellata,* the processus is less than half the width of the trochlea). The tuberosity for the flexor attachment forms two distinct scars on the proximocaudal surface, one lying proximal of the spatium intermetacarpale, and the more distal one slightly overlapping the proximal synostosis of the metacarpals (i.e. mostly within the spatium intermetacarpale), whereas in *Al. lathami* and *L. ocellata*, there is a single, long tuberosity with its distal end lying within the spatium intermetacarpale, and overlapping the synostosis proximally. At the distal end, there is a short but very deep sulcus tendineus running longitudinally on the caudal surface of the os metacarpale majus. The facies articularis digitalis minor projects much further distally than facies articularis digitalis major, as in *Al. lathami*, and differing from *L. ocellata* and *T. fuscirostris*, in which there is little distal projection of the facies articularis dig. minor.

**Table 1.** Long-bone measurements (mm) of *P. gallinacea*; TL, total length; PW, proximal width; SW, midshaft width; DW, distal width.

element/side	catalogue no.	TL	PW	SW	DW
lectotype					
tarsometatarsus (pL)	QM F1143	est. 147.5^a^	28.4	12.7	—
paralectotypes					
tarsometatarsus (pL)	QM F1134	—	28.9	—	—
tarsometatarsus (dR)	QM F5556	—	—	11.7	—
tarsometatarsus (dR)	QM F5557	—	—	—	29.5
referred material					
carpometacarpus (pR)	QM F1132	—	26.9	—	—
carpometacarpus (dR)	QM F1139	—	—	—	19.0
ulna (dR)	QM F5553	—	—	—	20.0

^a^Estimated length of lectotype QM F1143 is based on measurement from proximal end to the proximal edge of fossa metatarsi I (100 mm), added to the distal length of slightly narrower syntype QM F5556 measured from the proximal edge of fossa metatarsi I to the distal end (47.5 mm), so TL of QM F1143 is estimated to be a minimum of 147.5 mm.

**Coracoid:** The coracoid of this species has not previously been described. We refer a very large Pliocene specimen (SAM P50028; figure 4*i*–*l*) to this species, previously noted in the literature under specimen number FU2655 [35]. We refer SAM P50028 to *P. gallinacea* rather than to any other large extinct taxon because of its very large size and the following morphological similarities with the coracoid of the smaller species of *Progura* described below: the ventrolateral margin of the facies articularis humeralis projects strongly laterally as a rounded flange in ventral aspect; there is a deep sulcus on the ventral surface, between the ventrolateral margin of the facies articularis humeralis and the processus acrocoracoideus, thus the depth of the bone here between the ventral and dorsal surfaces is shallow in omal aspect; and the facies articularis humeralis is deeply concave. It also has the following features: the dorsal part of the facies articularis clavicularis projects strongly cranially as in *L. ocellata* (less projection in *Al. lathami*); and the ventral part of the facies articularis clavicularis does not project strongly over the ventral facies of the shaft. The very large size of the coracoid, and its wide shaft and large surface area for articulation with the scapula and humerus, is consistent with *P. gallinacea*, the largest known megapode species, having had a strong pectoral girdle, with no signs of reduction that would indicate flightlessness.

**Scapula:** A very large and robust specimen (QM F5558; figure 3*f*–*h*) from the late Pliocene Chinchilla Sand deposit, missing the acromion and most of the corpus scapulae, was originally referred to the Otididae by De Vis, and subsequently to *P. gallinacea* by van Tets [23]. As in all megapodes [21], there is a pneumatic fossa immediately latero-ventral of the facies articularis humeralis. There is a broad, longitudinal groove latero-dorsal to the facies articularis humeralis. Insofar as the bone can be measured, its dimensions are outside the size range of other large extinct megapodes described below. Measurements (mm): width of the facies articularis humeralis, 15.1; width of collum scapulae immediately distal of the facies articularis humeralis, 13.7; depth of collum scapulae immediately distal of facies articularis humeralis, 10.5.

**Tarsometatarsus:** In addition to the diagnostic features noted above, tarsometatarsi of this species (figure 3*a*–*i*) have the following characteristics. They are far larger than those of any known megapode species, with the estimated length of a complete bone being approximately 147.5 mm, based on the length of the proximal fragment that is the lectotype (QM F1143) and the distal fragment with the longest preserved shaft (QM F5556, paralectotype) laid side by side with the shafts appropriately overlapped. This is of comparable length to tarsometatarsi of the giant flightless stem-galliform *S. neocaledoniae*, but proximal width (28.4–28.9 mm) is some 10 mm narrower than in *S. neocaledoniae*, and distal width (29.5 mm; QM F5557) is around 12 mm narrower [21]. This is consistent with *P. gallinacea* having been a much lighter bird than the 27–34 kg *Sylviornis* [21] (see Body mass estimates). The trochleae are wide and deep, and are presumed to have articulated with very large phalanges, although none have been recovered. Measurements (mm): for TL, PW, SW and DW (table 1); depth troch. metatarsi II, 11.6 (QM F5557), 11.9 (QM F5556); depth troch. metatarsi III, 14.4 (QM F5557), 14.2 (QM F5556); depth troch. metatarsi IV, 11.2 (QM F5557).

### 3.2. Remarks on specimens previously referred to *Progura gallinacea*

All specimens from caves in the Naracoorte region (southeastern South Australia), and a proximal tarsometatarsus (QM F2769) from Gore Limestone Quarry (Darling Downs, southeast Queensland), that were originally referred to *Progura naracoortensis* by van Tets [23], but were included within the synonymy of *P. gallinacea* by Boles [24], are transferred herein to a new genus (see below). A further six bones from Wombeyan Caves, eastern New South Wales, which were also originally referred to *P. gallinacea* [23], and retained in that species by Boles [24] are also transferred to this new genus.

A distal right tarsometatarsus (QM F7033, Ravensthorpe, Darling Downs, Queensland) was previously referred to *P. gallinacea* [23] (where it was incorrectly reported as AM F7033). It is eroded and incomplete, but sufficiently well preserved to show that the trochleae do not match the morphology of the type specimens of *P. gallinacea*. Trochlea III is much more elongate than in Megapodiidae; the incisura between trochleae III and IV is much wider; and trochlea IV is narrower and dorsoventrally flatter but more laterally flared. It is provisionally identified as a phoenicopteriform.

A distal right ulna (AM F54723, Walli Caves, New South Wales) previously referred to *P. gallinacea* [23] does not belong to a megapode. Provisional examination suggests that it may belong to an undescribed eagle larger than extant wedge-tailed Eagle *Aquila audax*.

A partial left coracoid missing both ends (BMNH A3244, from an unknown cave (likely either Cathedral or Mitchell [=Breccia] Cave)) in Wellington Valley, near Wellington, New South Wales, was originally referred by Lydekker [49], as his catalogue number 43879, to a species of *Alectura* (as *Talegalla*) larger than extant *Al. lathami*. The specimen was later referred [23] to *P. gallinacea* because of its large size, but there is no other evidence that *P. gallinacea* inhabited the Wellington region (see below). The fossil may belong to the same genus and species as other large megapode fossils from Wellington, but a referral cannot be made without examining the specimen.

#### ***Progura campestris*** Shute Prideaux & Worthy, sp. nov.

##### (figures 5*b,g*,*l*,*q*,*v*, 6, 7*d*–*f* and 8–11)

**Zoobank ID:** urn:lsid:zoobank.org:act:02B438F7-C0F6-4B60-BA8D-C0F491D2CEB0

***Leipoa gallinacea*** (De Vis, 1888): Prideaux *et al*. (2007), *Nature*, 445: 423, table 1. Not *P. gallinacea* De Vis, 1888.
***Leipoa gallinaceae*** (De Vis, 1888): Wroe *et al.* (2013), *Proceedings of the National Academy of Sciences of the United States of America*, 110(22): 8779, figure 3; electronic supplementary material, table S1. Not *P. gallinacea* De Vis, 1888.

**Figure 6.**
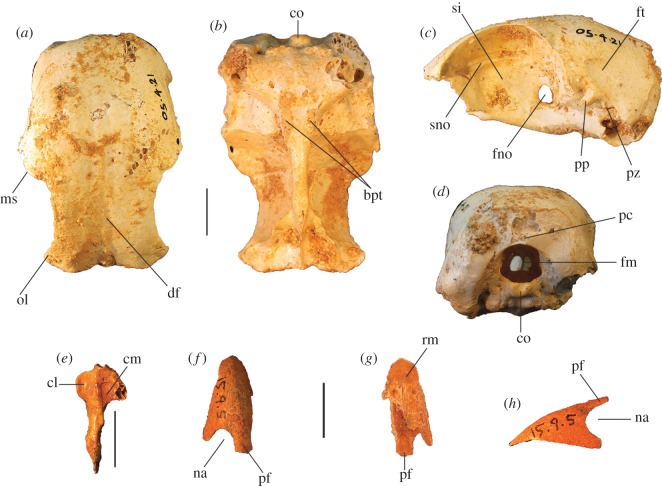
Cranial remains of *P. campestris* sp. nov. Cranium, WAM 05.4.21, in dorsal (*a*) ventral (*b*), lateral (*c*) and caudal (*d*) aspects; mandible, WAM 15.9.5, holotype, right articular fragment, in dorsal aspect (*e*); premaxilla, WAM 15.9.5, holotype, in dorsal (*f*), ventral (*g*) and lateral (*h*) aspects. bpt, processus basipterygoidei; cl, condylus lateralis; cm, cotyla medialis; co, condylus occipitalis; df, depressio frontalis; fm, foramen magnum; fno, foramen nervi optici; ft, fossa temporalis; ms, margo supraorbitalis; na, naris; ol, os lacrimale; pc, processus costalis; pf, processus frontalis; pp, processus postorbitalis; pz, processus zygomaticus; rm, rostrum maxillare; si, septum interorbitale; sno, sulcus nervi olfactorii. Scale bars, 10 mm.

**Figure 7.**
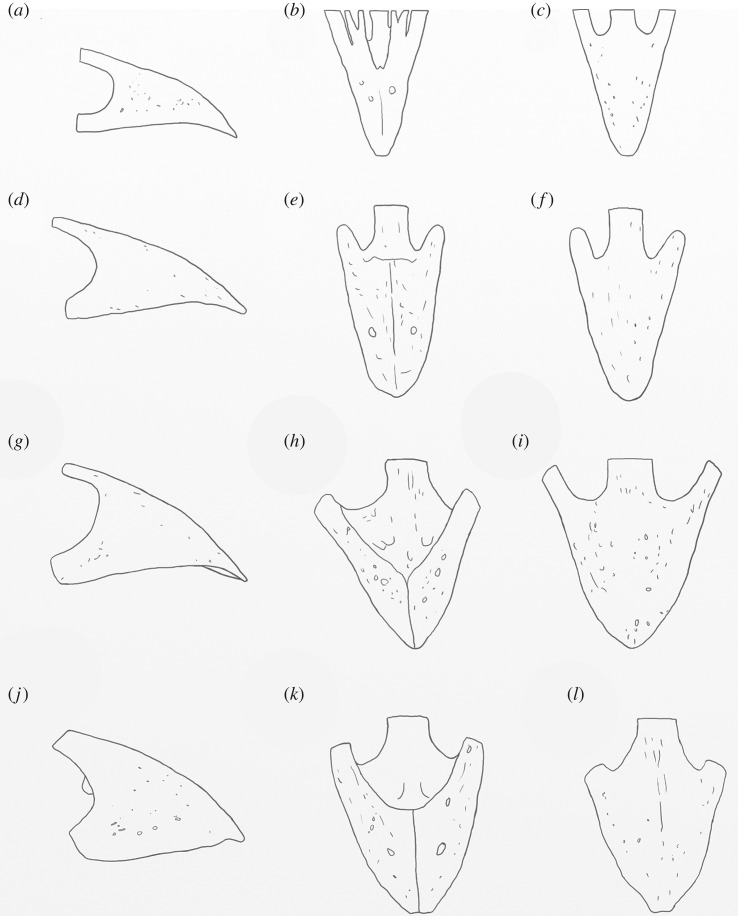
Shape comparison of megapode premaxillae: *L. ocellata* (*a*–*c*); *P. campestris* sp. nov. (*d*–*f*); *G. mcnamarai* sp. nov. (*g*–*i*); *La. naracoortensis* (*j*–*l*). (*a*,*d*,*g*,*j*) lateral view; (*b*,*e*,*h*,*k*) ventral view; (*c*,*f*,*i*,*l*) dorsal view. Outlines traced from photographs of specimens, with damaged portions mirrored from contralateral side where possible.

**Figure 8.**
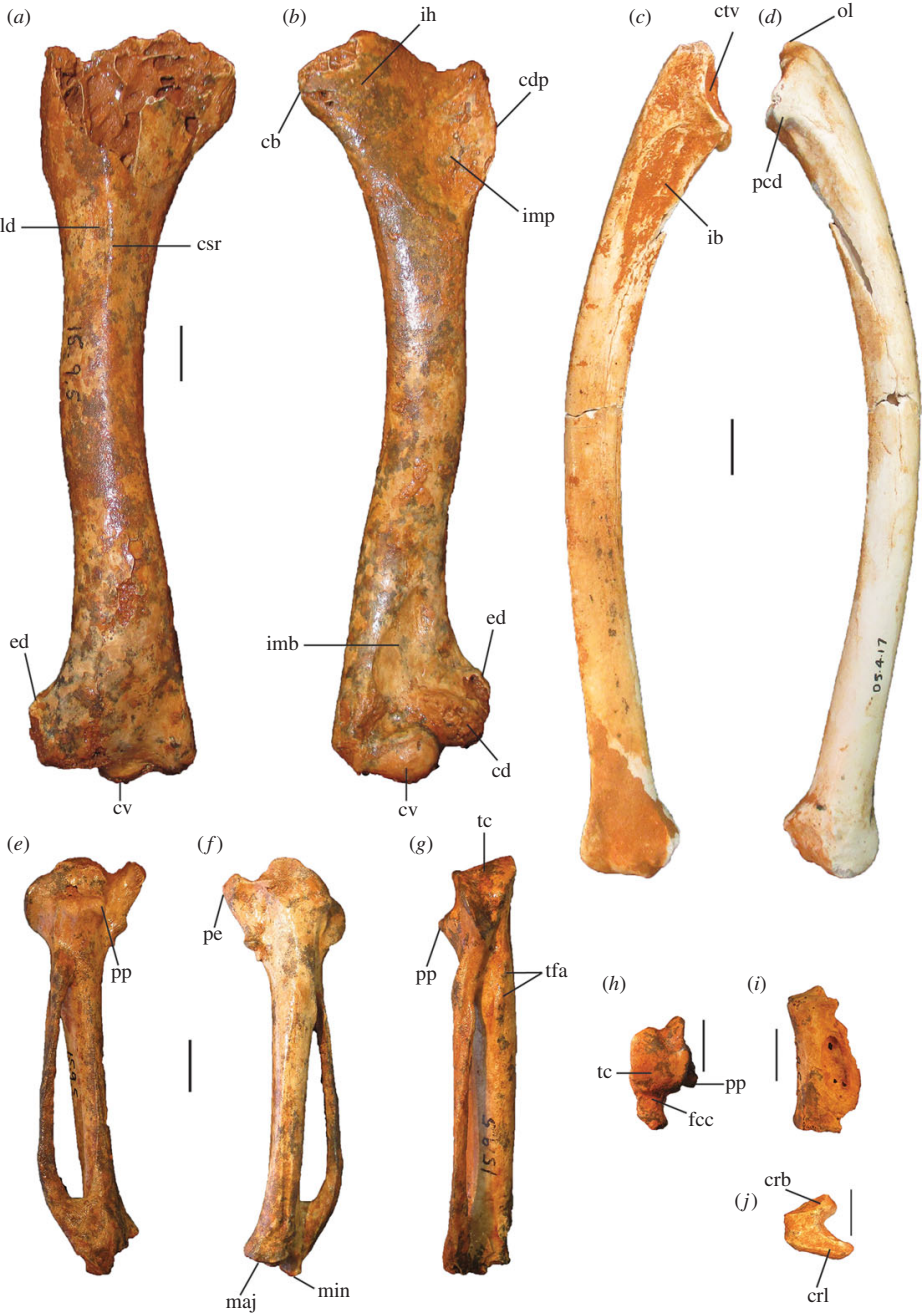
Wing elements of *P. campestris* sp. nov. Humerus, WAM 15.9.5, holotype, in caudal (*a*) and cranial (*b*) aspects; ulna, WAM 05.4.17, paratype, in ventral (*c*) and dorsal (*d*) aspects; carpometacarpus, WAM 15.9.5, holotype, left, in ventral (*e*) and dorsal (*f*) aspects, and WAM 15.9.5, holotype, right, in caudal (*g*) and proximal (*h*) aspects; phalanx dig. major, WAM 15.9.5, holotype (*i*); os carpi ulnare, WAM 15.9.5, holotype (*j*). cb, crista bicipitalis; cd, condylus dorsalis; cdp, crista deltopectoralis; crb, crus breve of os carpi ulnare; crl, crus longum of os carpi ulnare; csr, capital shaft ridge; ctv, cotyla ventralis; cv, condylus ventralis; ed, epicondylus dorsalis; fcc, fovea carpalis cranialis; ib, impressio brachialis ulnaris; ih, intumescentia humeri; imb, impressio m. brachialis; imb, impressio musculo brachialis; imp, impressio m. pectoralis; ld, attachment for m. latissimus dorsalis; maj, facies articularis digitalis major; min, facies articularis digitalis minor; ol, olecranon; pcd, processus cotylaris dorsalis; pe, processus extensorius; pp, processus pisiformis; tc, trochlea carpalis; tfa, tuberosity for flexor attachment. Scale bars, 10 mm.

**Figure 9.**
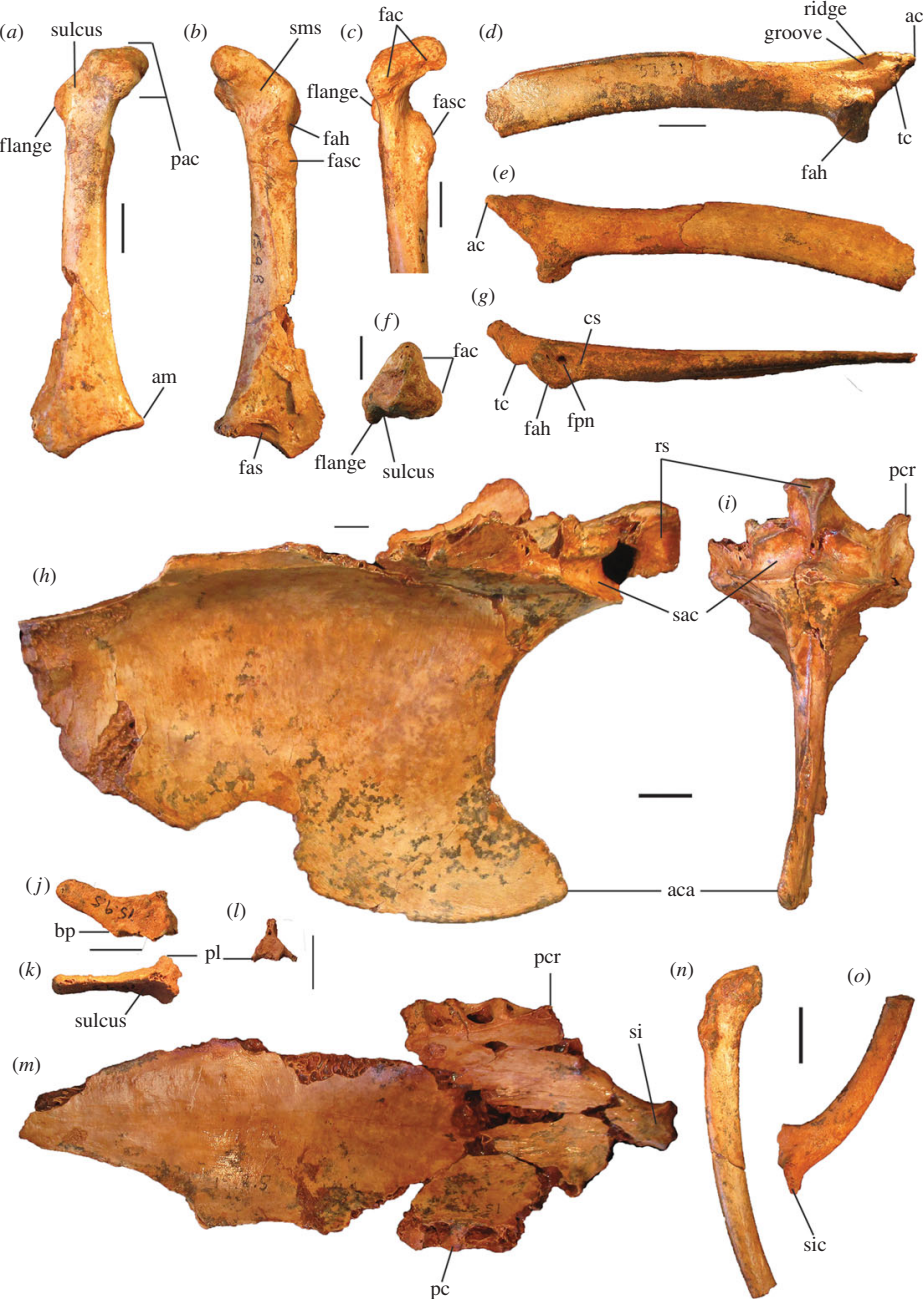
Pectoral and axial skeletal elements of *P. campestris* sp. nov. Coracoid, WAM 15.9.8, paratype, in ventral (*a*), dorsal (*b*), medial (*c*) and omal (*f*) aspects; scapula, 15.9.5, holotype, in lateral (*d*), medial (*e*) and ventral (*g*) aspects; sternum, WAM 15.9.5, holotype, in right lateral (*h*) cranial (*i*) and dorsal (*m*) aspects; pygostyle, 15.9.5, holotype, in lateral (*j*), dorsal (*k*) and cranial (*l*) aspects; clavicula, WAM 15.9.5, holotype, omal (*n*) and sternal (*o*) portions. ac, acromion; aca, apex carinae; am, angulus medialis; bp, basis pygostyli; cs, collum scapulae; fac, facies articularis clavicularis; fah, facies articularis humeralis; fas, facies articularis sternalis; fasc, facies articularis scapularis; fpn, foramen pneumaticum; pac, processus acrocoracoideus; pc, processus costalis; pcr, processus craniolateralis; pl, processus lateralis; rs, rostrum sterni; sac, sulcus articularis coracoideus; si, spina interna; sic, synostosis interclavicularis; sms, sulcus m. supracoracoidei; tc, tuberculum coracoideum. Scale bars, 10 mm.

**Figure 10.**
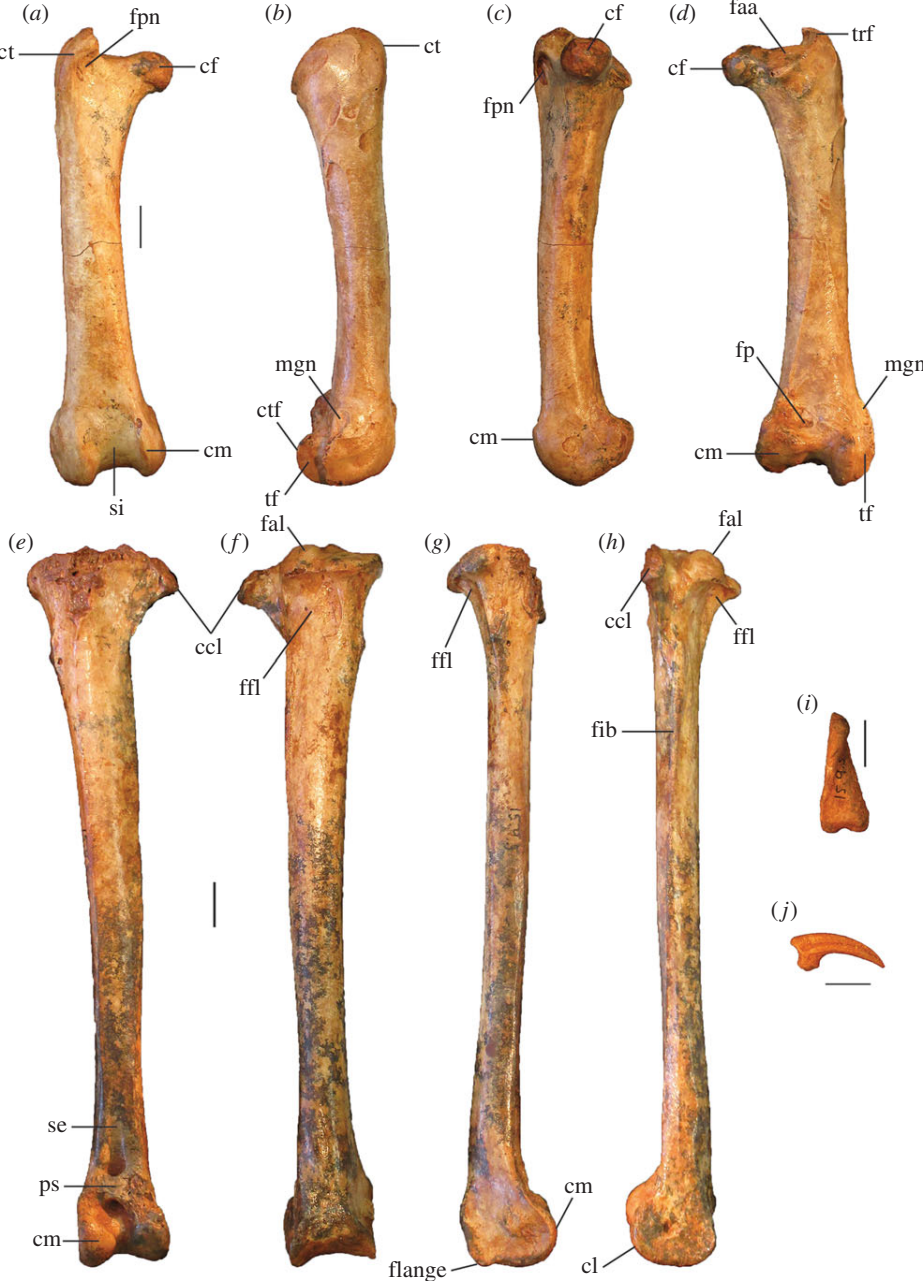
Leg and foot elements of the holotype of *P. campestris* sp. nov. WAM 15.9.5. Femur in cranial (*a*), lateral (*b*), medial (*c*) and caudal (*d*) aspects; tibiotarsus in cranial (*e*), caudal (*f*), medial (*g*) and lateral (*h*) aspects; os metatarsale I, left (*i*); ungual, dig. I, left (*j*). ccl, crista cnemialis lateralis; cf, caput femoris; cl, condylus lateralis; cm, condylus medialis; ct, crista trochanteris; ctf, crista tibiofibularis; faa, facies articularis antitrochanterica; fal, facies articularis lateralis; ffl, fossa flexoria; fib, crista fibularis; fp, fossa poplitea; fpn, foramen pneumaticum; mgn, impression for m. gastrocnemialis lateralis; ps, pons supratendineus; se, sulcus extensorius; si, sulcus intercondylaris; tf, trochlea fibularis; trf, trochanter femoris. Scale bars, 10 mm.

**Figure 11.**
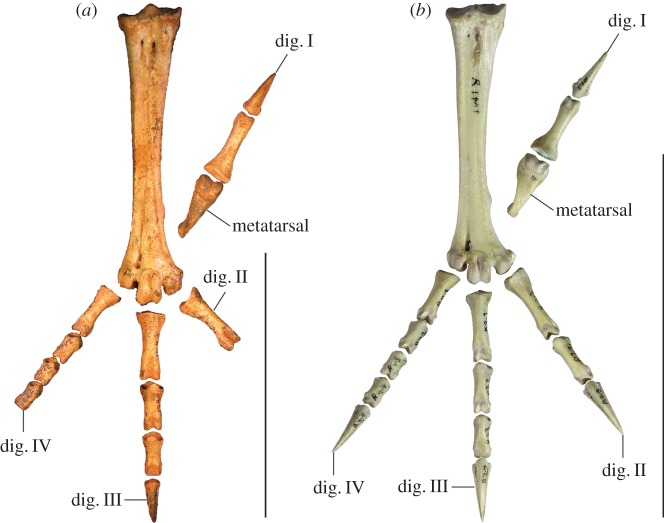
Reconstructed foot of extinct *P. campestris* sp. nov. (*a*) compared with foot of extant malleefowl *L. ocellata* (*b*), and scaled to the same size (scale bars, 10 cm). All bones of the extinct species are from the holotype skeleton WAM 15.9.5, with some images reversed to make a right pes. Note that the extinct species has a proportionally longer tarsometatarsus, but shorter digits, including shorter unguals on digits I and III. (Image of *L. ocellata* foot from figure 11 in Worthy *et al.* [21].)

**Holotype** (figures 5*b*,*g*,*l*,*q*,*v*; 6*e*–*h*; 7*d*–*f*; 8, 9*d*–*o*, 10 and 11*a*): WAM 15.9.5, associated remains of one adult individual, comprising the following elements: premaxilla, approximately 15 mm of the tip; right articular of mandible lacking tips of processus medialis and processus retroarticularis; vertebrae (cervicals 3 and 4, parts 5 others, three vertebral fragments, anterior fragment of synsacrum, pygostyle); sternum, preserving most of its length, the full depth of the keel and most of the rostrum sterni; two fragments of clavicula; R coracoid, sternal and omal fragments; R scapula, complete apart from missing distal tip; L humerus, missing caput humeri; dR ulna, approximately 3 cm fragment; L radius; L, R carpometacarpus; R os carpi ulnare; R phalanx I digiti majoris; R phalanx II digiti majoris; R femur; L, R tibiotarsus, L missing the crista cnemialis cranialis, R missing its proximal half; L, R tarsometatarsus, the L missing the hypotarsus and half of cotyla lateralis; 14 pedal phalanges (L: I.1, I.2, I ungual, II.1, II.2, III.1, IV.1, IV.2, IV.3, IV.4; R: III1, III.2, III.3, III ungual). Number of individual bones = 51.

**Type locality:** Leaena's Breath Cave, Thylacoleo Caves, Nullarbor Plain, Western Australia (figure 2; see Key locations).

**Paratypes**: ***Leaena's Breath Cave, Nullarbor Plain, Western Australia***—WAM 15.9.16, 15.9.17, 15.9.18, 15.9.27, 15.9.28, 15.9.29, 15.9.30, 7 cervical vertebrae; WAM 15.9.13, dL humerus; WAM 15.9.14, pL humerus; WAM 15.9.15, pR humerus; WAM 15.9.32, L os carpi ulnare; WAM 15.9.9, R carpometacarpus; WAM 15.9.8, R coracoid; WAM 15.9.11, L tarsometatarsus; WAM 15.9.12, pR tarsometatarsus; WAM 15.9.1, dL tarsometatarsus; WAM 15.9.7, pL tarsometatarsus; WAM 15.9.31, phalanx I.1, L and R.

**Referred material: *Last Tree Cave, Nullarbor Plain, Western Australia***—WAM 05.4.21, cranial vault (figure 6*a*–*d*); WAM 05.4.17, L ulna (figure 8*c*,*d*); WAM 04.6.1, dR tarsometatarsus.

**Stratigraphy and age:** The disarticulated but associated holotype material was excavated by G. J. Prideaux on 10–11 August 2011, from a depth of 110–115 cm below the current sediment floor, in stratigraphic Unit 3, Quadrat 3, Pit B, Leaena's Breath Cave. Reversed magnetic polarity and the composition of the vertebrate assemblage indicates that the Unit 3 sediments were deposited during the Matuyama Chron (2.58–0.78 Ma; Early Pleistocene) [28]. The paratypes were collected from the sediment floor of Leaena's Breath Cave by J. A. Long in July 2002, and are of unknown Pleistocene age. The referred specimens, including the referred cranium (WAM 05.4.21), were collected from the surface of The Ossuary deposit in Last Tree Cave by J. A. Long in July 2002, and are undated but of probable Pleistocene age, given their co-deposition with extinct species of marsupial that occur only in Pleistocene deposits elsewhere (e.g. *Procoptodon goliah*).

**Diagnosis:** A species of megapode larger than any extant member of the Megapodiidae, with a tarsometatarsus conforming with *Progura*, but approximately one-third shorter than that of the type species, and characterized by the following features. (i) The fossa parahypotarsalis medialis is shallow and restricted to the first 25% of length. (ii) The medial margin of the shaft is thick, and lacks a shallow sulcus between it and the foramina vascularia–sulcus extensorius, lending the dorsal facies of the shaft a flat appearance in medial aspect. (iii) The midshaft region is plantarly convex. (iv) The shaft is robust (minimum width 10.1% of total length). (v) It lacks a deep depression for the impressio lig. collateralis medialis. (vi) The facet for metatarsal I measures around one-third of the width of the shaft (half the shaft width in *P. gallinacea*). (vii) In dorsal aspect, the rims of trochlea metatarsi III converge proximally.

**Differential diagnosis:** Apart from much larger size, the tarsometatarsus of the type species *P. gallinacea* differs by having: (i) a deeper fossa parahypotarsalis medials extending to half the shaft length; (ii) a thinner medial margin, and a twisted dorsal facies; (iii) a flat plantar midshaft; (iv) a more gracile, distally tapered shaft (minimum width 8.6% of total length); (v) a deep depression for the impressio lig. collateralis medialis; (vi) a wider facet for metatarsal I (half the shaft width); (vii) parallel rims of trochlea metatarsi III in dorsal aspect.

**Etymology:** *campestris* = ‘from the plain’ (campos = ‘plain’ or ‘field’, Latin), referring to the habitat of this species on the flat limestone plateau of the Nullarbor Plain.

**Description and comparisons:** The holotype skeleton preserves most major elements in excellent condition, with the paratypes and referred material preserving additional elements and anatomical detail. Features of the skeleton are described below and are compared with extinct and extant taxa. Long-bone measurements are given in table 2, and measurements of phalanges in table 3. Additional measurements are given in text where applicable. Photographs of the tarsometatarsus are shown in figures 5*b*,*g*,*l*,*q*,*v* and 11, and other skeletal material in figures 6–10.

**Table 2.** Long-bone measurements (mm) of *P. campestris* sp. nov., holotype and referred material; TL, total length; PW, proximal width; SW, midshaft width; DW, distal width.

element/side	catalogue no.	TL	PW	SW	DW
holotype					
coracoid, R	WAM 15.9.5	80.3	17.6^a^	9.8	>24^a^
scapula, R	WAM 15.9.5	—	—	12.3	—
humerus, L	WAM 15.9.5	—	34.6	14.6	29.0
ulna, dR	WAM 15.9.5	—	—	—	17.8
radius, L	WAM 15.9.5	140.7	9.5	6.3	13.1
carpometacarpus, L	WAM 15.9.5	77.4	22.8	16.1^b^	14.4
femur, R	WAM 15.9.5	106.6	27.5	14.0	26.2
tibiotarsus, L	WAM 15.9.5	158.7	22.4^c^	11.7	20.4
tarsometatarsus, R	WAM 15.9.5	105.6	23.0	10.6	23.9
referred material					
coracoid, R	WAM 15.9.8	79.0	20.0^a^	9.8	>23.5^a^
ulna, L	WAM 05.4.17	148.2	16.8	10.2	18.0
carpometacarpus, R	WAM 15.9.9	75.1	21.0	—	—
tarsometatarsus, dR	WAM 04.6.1	—	—	—	22.5
tarsometatarsus, pL	WAM 15.9.7	—	23.3	—	—
tarsometatarsus, L	WAM 15.9.11	93.4	21.3	10.0	—

^a^For coracoid, PW, omal width; DW, sternal width.

^b^For the carpometacarpus, SW, maximum width measured cranio-caudally at the widest arc of the os carpus minus.

^c^For tibiotarsus, PW is measured across the articular surface, and excludes the crista cnemialis lateralis.

**Table 3.** Measurements (mm) of pedal phalanges of *P. campestris* holotype; PW, proximal width; PD, proximal depth; SW, shaft width; DW, distal width; DD, distal depth; TL, total length.

phalanx	PW	PD	SW	DW	DD	TL
L I.1	3.8	5.3	6.8	10.2	6.5	26.3
L I.2	11.2	7.2	5.5	7.0	5.9	27.4
L I ungual	7.2	8.3	4.3	—	—	21.8
L II.1	10.2	8.6	5.5	6.9	6.9	26.7
L II.2	7.3	7.2	6.1	5.8	5.3	13.1
R III.1	10.9	10.5	5.8	7.9	6.7	26.2
R III.2	9.0	8.4	6.0	6.9	6.1	18.8
R III.3	8.0	8.1	5.7	6.7	5.2	16.6
R III ungual	6.1	7.4	3.8	—	—	18.6
L IV.1	9.2	7.8	5.2	7.0	6.5	20.6
L IV.2	7.1	7.3	6.0	6.0	5.4	12.9
L IV.3	7.1	6.6	6.1	6.1	4.7	11.2
L IV.4	6.4	6.4	5.2	6.2	4.7	12.0

**Cranial bones:** The articular mandible fragment of the holotype (figure 6*e*) is relatively small and indicates that the bill of this species was gracile rather than robust (width of ramus immediately anterior of the cotyla lateralis, 2.3 mm; cf. 1.7 mm in *L. ocellata*). The holotype also preserves the anterior portion of the rostrum maxillare (premaxilla) (figure 6*f*–*h*), with approximately 0.5 cm long portion of the processus frontalis and a similar length of the left processus maxillaris. The shape of the anterior edge of the left naris is preserved, showing a wide opening. In size and shape, the premaxilla is almost identical to that of *Al. lathami*, and is considerably larger than in *L. ocellata* or *M. reinwardt*. The tip is relatively elongate. A reconstruction of the shape of the anterior rostrum is shown in figure 7.

The referred cranium (WAM 05.4.21; figure 6*a*–*d*) is similar in length and depth to that of *Al. lathami*, but is broader. In lateral aspect, the shape of the braincase is dorsoventrally flattened and elongate as in *Al. lathami*, rather than having the shorter, domed profile of *L. ocellata* and species of *Megapodius*, or having the bulging casque of *Ma. maleo*. Unlike in *L. ocellata*, *Al. lathami*, *M. reinwardt* or *Ma. maleo*, the lacrimals flare widely laterally anterior to the orbits and are wholly fused to the nasals, and the margo supraorbitalis flares laterally in the posterior section of the orbits, indicating bony protection of the eyes (figure 6*a*). The occipital region is morphologically quite similar to that of *Al. lathami*, although the condylus occipitalis is larger, and the foramen magnum is deeper than wide (wider than deep in *Al. lathami* and *L. ocellata*). The processus basipterygoidei are about the same size as in *Alectura* and are also elongate (short in *L. ocellata*) but are placed slightly more posteriorly than in *Alectura*. Preserved on the left side, the processus postorbitalis and the processus zygomaticus are narrowly separated, allowing only a tiny fossa muscularis temporalis, but whether or not they were fused distally, or how large the aponeurosis zygomatica was, cannot be determined as the tips of both are broken. As in other species of megapode, the septum interorbitale is perforated by a large foramen nervi optici. A marked sulcus nervi olfactorii traverses the dorsal side of the septum interorbitalis (figure 6*c*), which exits the braincase from a very small foramen nervi olfactorii, unlike the large and obvious foramen seen in *L. ocellata*, *Al. lathami*, *M. reinwardt* and *Ma. maleo*. Measurements (mm): premaxilla, maximum depth measured at the level of the anterior edge of the naris, 7.2; preserved length of the cranium, 47.6; maximum depth, 24.8; anterior width across fused lacrimals, 24.8; minimum interorbital width of osa frontales, 18.7; maximum diameter of the orbital fossa, 22.8; width of the foramen magnum, 6.5; depth of the foramen magnum, 6.9.

**Vertebrae:** The holotype and paratypic cervical vertebrae are morphologically similar to those of other megapodes. Cervical #4 is more elongate than in *L. ocellata* and anterior and caudal widths are the same (*L. ocellata* is wider caudally). The pygostyle is about the same length as in *Al. lathami*, but it is deeper and broader with more distinct, laterally projecting processus laterales anteriorly (smaller and more ventrally directed in *Alectura*; lacking entirely in *Leipoa*), and has a blunt rather than pointed caudal tip. The sides have large shallow sulci (absent entirely in *L. ocellata*). The distal end is directed dorsally at about 45° to the anterior section (figure 9*j*), whereas in *Al. lathami*, the dorsal profile of the pygostyle is straight in lateral aspect, and in *L. ocellata*, the pygostyle is curved slightly downwards. No thoracic vertebrae or notaria were recovered from the excavations.

**Humerus:** Most of the left humerus of the holotype is preserved, but the proximal end, including the caput humeri, is missing (figure 8*a*,*b*). Two paratypic proximal fragments (WAM 15.9.14 and 15.9.15) preserve the caput, tuberculum ventrale and crista bicipitalis. Megapodes have relatively homogeneous humeral morphology, and the humerus of *P. campestris* shares features typical of the Megapodiidae (see family diagnosis). It has distinguishing features as follows: the crista bicipitalis is relatively short proximodistally, as seen in WAM 15.9.14 and WAM 15.9.15; the tuberculum ventrale is not very prominent caudally and does not project further than the caudal facies of the caput humeri in proximal aspect; the shaft is relatively narrow in caudal aspect as it approaches the proximal end, but the margo caudalis is elevated as it approaches the proximal end because of a strong capital ridge, making the shaft proportionally thick here; the crista deltopectoralis has a thick base but forms a sharp crest in its distal section and the adjacent impression for m. pectoralis on the cranial surface is deeply concave; the distal end is relatively narrow; the condylus ventralis is prominent distally; and the tuberculum supracondylare ventrale is relatively small. Measurements: for PW, SW and DW, see table 2; if complete, total length of the holotype humerus would be approximately 144 mm.

**Ulna:** The holotype lacks a complete specimen, but the paratype ulna (WAM 05.4.17) is well preserved (figure 8*c*,*d*). The shaft is curved in dorsal aspect and is dorsoventrally compressed. The impression for the m. brachialis is deep, and the olecranon is orientated somewhat ventrally in cranial aspect. The condylus ventralis ulnaris does not protrude dorsally, as in most megapodes. Measurements: for TL, PW, SW and DW, see table 2.

**Radius:** The holotype preserves a complete L radius. Measurements: for TL, PW, SW and DW, see table 2.

**Carpometacarpus:** The carpometacarpi (figure 8*e*–*h*) are considerably smaller than those of *P. gallinacea* (approx. 25% shorter), but are morphologically quite similar, including having two distinct scars/tuberosities for the flexor attachment, the distal one being entirely within the spatium intermetacarpale, unlike the single tuberosity seen here in *Al. lathami* and *L. ocellata.* Carpometacarpi of *P. campestris* differ from *P. gallinacea* by having: a relatively longer, more proximally orientated processus extensorius that projects further proximally than the ventral rim of the trochlea carpalis (does not do so in *P. gallinacea*); more caudal projection of the ventral rim of the trochlea carpalis; a more prominent processus pisiformis. In caudal aspect, the proximal end of the os metacarpale minus is directed ventrally, and the ventral rim of the trochlea carpalis cranialis diverges markedly from the long axis of the os metacarpus majus. The spatium intermetacarpale is relatively narrow. They differ from all species examined, including *P. gallinacea*, by having a shallow fovea carpalis cranialis. Measurements: for TL, PW, DW and maximum width (measured cranio-caudally at the widest arc of the os carpus minus), see table 2.

**Coracoid:** The holotype includes omal and sternal coracoid fragments. Among the paratypes is a slightly more slender, coracoid (WAM 15.9.8), found in association with the holotype skeleton (Quadrat 3, Pit B, Unit 3, 110–115 cm, Leaena's Breath Cave) but belonging to another individual (figure 9*a*–*c*,*f*). It is complete apart from minor damage to the shaft and to the sternal end. Coracoids of this species are considerably smaller than that of *P. gallinacea*, and are small compared with other elements of the skeleton (see Simpson log-ratio diagram, figure 22). They are further distinguished by the following features of the omal end (a sternal end is lacking for *P. gallinacea*): the dorsal part of the facies articularis clavicularis does not project as far cranially; and the ventral part of the facies articularis clavicularis projects more strongly above the ventral facies of the shaft. Other features of the coracoid of *P. campestris* include: a relatively slender shaft; a short processus acrocoracoideus (as in *L. ocellata*, and differing from *Alectura*, in which it is elongate); a facies articularis clavicularis with little sternal projection (as in *L. ocellata*, differing from *Al. lathami*, in which the facies projects); a ventromedial part of the facies articularis clavicularis that is prominent and overhangs the medial margin of the sulcus m. supracoracoidei; a dorsal portion of the facies articularis clavicularis that projects much further medially than the facies articularis scapularis in dorsal aspect; an angulus medialis that is smoothly contiguous with the medial shaft, rather than medially projecting; and a facies articularis sternalis with a relatively small surface area and a straight distal margin in dorsal aspect. Measurements: for TL, SW, omal width and sternal width, see table 2.

**Scapula:** The holotype skeleton preserves one nearly complete scapula, missing only the distal tip (figure 9*d*,*e*,*g*). It is much smaller than the scapula of its congener *P. gallinacea*. It has features as follows: the pneumatic fossa on the ventral surface immediately distal of the facies articularis humeralis is reduced; the acromion is quite short and is directed cranially; the process for attachment of the ligamentum acrocoraco-procoracoideum medially on the acromion is reduced; and there is a longitudinal groove latero-dorsal to the facies articularis humeralis, with the dorsal margin above it forming a narrow crest. Measurements (mm): for SW, see table 2; max. width of the facies articularis humeralis, 11.9; width distal of the facies articularis humeralis, 10.9; depth distal of the facies articularis humeralis, 8.1; length from distal rim of facies articularis humeralis to tip of acromion, 23.6 mm.

**Sternum:** The holotype includes a partial sternum (figure 9*h*,*i*,*m*), which preserves the rostrum sterni, the carina largely complete except for perhaps 25 mm of length caudally, most of the dorsal surface of the pars cardiaca, the sulci articularis coracoideii, three processus costali on each side (although the presence of a fourth cannot be established due to breakage), but the specimen lacks the trabecula lateralis and left trabecula intermedia. The robust, and in dorsal view, triangular, spina interna is joined to the spina externa by a thin vertical blade of bone cranially as in other megapodes, enclosing a round foramen approximately 6 mm in diameter. The carina is deep and cranially recurved at its tip (figure 9*h*), as in extant megapodes. Caudally, on the right side, the preserved trabecula intermedia encloses the original margin of the incisura medialis, with minimally approximately 29 mm of that margin preserved caudal to it. Measurements (mm): maximum depth, measured from the top of the processus craniolateralis to the base of the carina, approximately 83; preserved length, 120; estimated total length, 145; length from spina interna to the anterior margin of incisura medialis, 94; width at first processus costalis, 45.

**Femur:** The holotype preserves a complete right femur in excellent condition (figure 10*a*–*d*). It is comparatively short and stout, with its length about equal to that of the associated tarsometatarsi. Proximally, the cranial surface adjacent to the crista trochanteris and level with the caput has a deep, pneumatized fossa (figure 10*a*,*c*), as in most compared extant taxa, except for *T. fuscirostris* and *Ma. maleo*. The caudal surface lacks the large pneumatic foramen adjacent to the facies articularis that is present in *T. fuscirostris*, but which is absent in all other extant taxa examined. The crista trochanteris is slightly damaged at its proximal end, but it appears to have been relatively short. At its proximal end, the crista is medially directed enclosing a fossa trochanteris, while its cranial margin is elevated from the shaft and drops steeply to the facies articularis antitrochanterica. In proximal aspect, the cranial edge of the crista trochanteris, the facies artic. antitrochanterica and the caput femoris form a smooth concave curve as in *Al. lathami*, whereas in all other taxa examined the cranial edge of the facies artic. antitrochanterica forms a rather straight line between the crista and the caput. In proximal aspect, the caudo-medial edge of the facies artic. antitrochanterica projects strongly caudally adjacent to the caput femoris, forming an angle of approximately 120° with the caput as in *M. reinwardt* and *Al. lathami* (approx. 150° angle in *T. fuscirostris*, *L. ocellata*). The collum femoris is short and slightly constricted. At the distal end, the sulcus patellaris is proportionally broader and shallower than in all other taxa examined, and it has a more u-shaped profile in distal aspect. As in other megapodes, the impression of the m. gastrocnemialis lateralis is a large, deep pit on the caudo-lateral surface just proximal of the trochlea fibularis. The fossa poplitea is very shallow and bound medially by a short acute crista supracondylaris medialis. Measurements (mm): for TL, PW, SW and DW, see table 2; proximal depth, 24.5; min. shaft circumference, 43.9.

**Tibiotarsus:** The most complete specimen (L tibiotarsus of the holotype) is well preserved, missing only the crista cnemialis cranialis (figure 10*e*–*h*). The crista fibularis is very weakly expressed. Proximally, the crista cnemialis lateralis is proportionally wide as in *M. reinwardt* and *L. ocellata* (smaller in *T. fuscirostris*, *Al. lathami* and *Ma. maleo*). In proximal aspect, the incisura tibialis is narrow, meaning that the facies articularis lateralis and the crista cnemialis lateralis are closely spaced. At the distal end, the pons supratendineus is proximodistally long as in *L. ocellata* and *Al. lathami* (medial side of the pons is markedly more constricted in all other taxa). The epicondylus medialis is not highly protuberant, and is completely obscured by the condylus medialis in cranial aspect as in *T. fuscirostris*, *Ae. arfakianus* and *Ma. maleo* (highly protuberant and visible beyond the medial rim of the condylus medialis in all other species examined). The retinaculi m. fibularis are less marked than in all taxa examined apart from *T. fuscirostris*. In medial aspect, the disto-caudal rim of the trochlea cartilaginis tibialis terminates in a slight flange where it merges with the condylus medialis (figure 10*g*), which is present but less exaggerated in other species. Just proximal of this flange, the caudal rim of the trochlea cartilaginis is slightly indented, whereas in all other species examined, the rim is rounded here. Measurements (mm): for TL, PW, SW and DW, see table 2; width of the proximal end without the crista cnemialis lateralis, i.e. the articular surfaces only, 20.8; depth of the condylus lateralis, 19.3; depth of the condylus medialis, 21.6; min. shaft circumference, 32.5.

**Tarsometatarsus:** In addition to the features noted above in the diagnosis, tarsometatarsi of *P. campestris* (figures 5 and 11*a*) have the following features. They are within the length range of *Al. lathami* (86.8–95.4 mm) but are longer than in all other extant megapodes and both absolutely and proportionally much larger in other dimensions than in all other extant species (see proportional comparison in appendix A). Measurements (mm): for TL, PW, SW and DW, see table 2; width troch. metatarsi II, (WAM 15.9.5) 9.2, (WAM 15.9.1) 10.9; width troch. metatarsi III, (WAM 15.9.5) 12.8, (WAM 04.6.1) 11.1, (WAM 15.9.1) 11.7; width troch. metatarsi IV, (WAM 15.9.5) 9.0.

**Phalanges:** Most of the foot is represented, missing only one phalanx and ungual of digit II, and the ungual of digit IV. The pes in figure 11*a* is reconstructed from left and right bones from the holotype. The toes are robust compared with all extant megapodes, but overall the reconstructed foot is of similar size to that of *Al. lathami* despite *P. campestris* being a much larger bird, thus the foot is proportionally small compared with overall body size. The ungual of digit I is slightly longer than in *Al. lathami*, but it is much stouter (deeper than wide) and has a more curved rather than a straight, elongate profile in lateral aspect. The ungual of digit III is slightly shorter than that of *Al. lathami*, but is also deeply curved rather than elongate. Compared with the pes of *L. ocellata* (figure 11), the tarsometatarsus is proportionally long and the digits proportionally short as a proportion of overall length of the pes. The preserved unguals (digits I and III) are proportionally shorter and stouter than in *L. ocellata*. Overall, these observations indicate that this species was less well adapted for mound-building and may have been a burrow-nester. Measurements: see table 3.

**Remarks:** So far this is the only very large extinct species of megapode known from the western two-thirds of Australia. It differs in size and morphology from its larger congener *P. gallinacea* as noted in the diagnosis and description, and these appear to have been allopatric species. Thus, there is no evidence that these represent members of a single sexually dimorphic species. Temporal overlap is more difficult to establish due to poor age constraints on Pleistocene material for *P. gallinacea* from the Darling Downs, thus an ancestral relationship between the two species cannot be ruled out (but see Phylogenetic analysis).

#### ***Latagallina*** Shute, Prideaux & Worthy, gen. nov.

**Zoobank ID:** urn:lsid:zoobank.org:act:8D617BBB-E082-442B-81A1-70AE6420B149

**Type species:** *Progura naracoortensis* van Tets, 1974

**Included taxa:** *Latagallina naracoortensis* (van Tets, 1974); *Latagallina olsoni* sp. nov. (see below)

**Diagnosis:** A genus of megapode distinguished from all other genera by the following unique combination of features of the tarsometatarsus and femur. **Tarsometatarsus:** (i) The shaft is stout and does not taper markedly towards the distal end, and the proximal and distal ends are proportionally wider than in other genera (proximal width = 23.6–26.0% of total length; distal width = 25.5–26.2% of total length). (ii) The shaft flares widely proximomedially where it meets the cotyla medialis. (iii) Dorsally, the foramen vascularis proximalis medialis is larger than its lateral counterpart, and is located slightly more distally. (iv) The sulcus infracotylaris dorsalis is deep, and is confined by thick, raised areas of bone laterally, medially and proximally. (v) The tuberositas m. tibialis cranialis comprises two elongate, parallel ridges, the medial ridge being broader and more elevated from the shaft than the lateral. Both are offset laterally from the midline of the bone shaft, and are situated distal of the foramen vascularis proximalis medialis. (vi) The lateral impressio retinaculum extensorii is placed slightly further distally than its medial counterpart, and is immediately proximomedial to the foramen proximalis medialis, abutting the sulcus infracotylaris dorsalis. (vii) The hypotarsus is plantarly extended (proximal part of medial hypotarsal ridge is about 50% of the depth of the cotyla medialis in medial aspect), and the junction between the medial hypotarsal crest and the plantar facies is abrupt, meeting the shaft more or less at 90° in medial aspect. (viii) The fossa parahypotarsalis medialis is very wide and deep and extends just past midlength of the bone. **Femur:** (i) Femora of species of *Latagallina* are long relative to tarsometatarsus length (ratio of 1.2). (ii) There is a large, round pneumatic foramen on the proximocaudal facies, level with the caput femoris and immediately distal of the facies articularis antitrochanterica. This is considered an autapomorphy of the genus, convergent in *Talegalla*.

**Differential diagnosis:** The tarsometatarsi of extant and extinct genera of megapodes differ from *Latagallina* as follows. (i) The shaft is more elongate/gracile in *Progura*, *Macrocephalon*, *Megapodius*, *Talegalla*, *Aepypodius* and *Alectura.* (ii) The shaft does not flare strongly proximomedially where it meets the cotyla medialis in *Progura*, *Macrocephalon*, *Aepypodius* or *Alectura*. (iii) On the dorsal surface, the foramina vascularia proximalia are of equal size and are equidistant from the proximal end in *Progura*, *Macrocephalon* and *Talegalla*. (iv) The sulcus infracotylaris dorsalis is shallow in *Progura* and *Macrocephalon* (variable among species of *Talegalla*), and in *Leipoa*, the sulcus is bounded by thick ridges of bone laterally and medially, but not proximally. (v) In *Leipoa*, the tuberositas m. tibialis cranialis comprises only a single short protuberance. In all other examined genera, the tuberositas comprises two parallel ridges. In *Progura*, *Macrocephalon* and *Alectura*, these are short, of about equal size, and are placed symmetrically with respect to the midline of the shaft. In *Megapodius*, they are long and the medial is broader as in *Latagallina*, but both are placed centrally on the shaft rather than being offset laterally. In *Progura* and *Macrocephalon*, the tuberosities are separated from the foramina vascularia proximalia by a long gap. (vi) In *Progura*, the impressiones retinaculi extensori are less prominent and both are placed well proximal of the level of the foramina vascularia proximalia. In *Macrocephalon*, they are more prominent, the medial one slightly more proximal than the lateral, and both are placed proximal of the level of the foramina vascularia proximalia. In *Alectura*, they are prominent, about level with one another, and slightly proximal of the level of the foramina vascularia proximalia. Position is variable between species of *Talegalla.* (vii) The proximal part of the hypotarsus is dorsoplantarly shallower in all taxa apart from *Leipoa* and *Megapodius. Megapodius* is distinguished by having a hypotarsus that is strongly recurved distally, forming a deep hook in lateral/medial aspects. In *Leipoa*, the hypotarsus has a similar profile to *Latagallina*, but differs by having a medial hypotarsal crest that tapers distally where it meets the plantar shaft facies, rather than forming an abrupt 90° angle in medial aspect. (viii) The fossa parahypotarsalis medialis is narrower, shorter and shallower in *Progura*, *Macrocephalon*, *Talegalla*, *Aepypodius* and *Alectura.* Femora of extant and extinct genera of megapode differ from *Latagallina* as follows. (i) The femur is shorter relative to the tarsometatarsus in *Alectura*, *Megapodius*, *Progura* and *Talegalla* (ratio of 1.0)*.* (ii) All extant megapode genera apart from *Talegalla*, and extinct *Progura*, lack a large pneumatic foramen on the proximocaudal facies of the femur. In *Talegalla*, the foramen is irregularly shaped and criss-crossed by trabeculae (a foramen is also seen here in members of some other galliform families on other continents, including the Miocene-aged *Ameripodius* in the Quercymegapodiidae and in extant *Argusianus* in the Phasianidae [50]).

**Etymology:** *Latagallina *= ‘broad hen’ (lata = ‘broad’, adjective, Latin; gallina = ‘hen’, noun, Latin). The name *Latagallina* refers to the stout, short-legged build of members of this genus. Gender is feminine.

**Remarks:** It was previously suggested [24] that the type species for this genus was not morphologically separable from *P. gallinacea*, and it was once interpreted as the smaller form (probably the female) of a single sexually dimorphic species [25]. In this paper, we note substantial differences between *Progura* and *Latagallina* throughout the skeleton, further reflected in the results of our phylogenetic analysis (see below). Our observations support Olson's remark that *P. gallinacea* De Vis, 1888 and ‘*Progura*’ *naracoortensis* van Tets, 1974 belong in separate genera [26], and we have thus erected *Latagallina* as a new genus*.* Species of *Latagallina* are also readily distinguished from *L. ocellata*, and we reject the proposed synonymy of *Progura/Latagallina* with *Leipoa*, which was based mainly on skeletal material of *La. naracoortensis* (cf. [24]).

**Geological range:** Early to Late Pleistocene (data herein).

**Geographical range:** Members of this genus are known to occur in southeastern Queensland, eastern New South Wales, southeastern South Australia and the Nullarbor Plain, Western Australia.

#### ***Latagallina naracoortensis*** (van Tets, 1974)

##### (figures 5*c*,*h*,*m*,*r*,*w* and 12–16)

**Synonyms:**

***Progura naracoortensis*** van Tets, 1974: *Transactions of the Royal Society of South Australia* 98: 214, figures 1–4; Henschke's Cave, near Naracoorte, southeastern South Australia.
***Progura gallinacea*** De Vis, 1888; Boles, 2008: *Oryctos* 7: 199, 205 figures 4 and 5, in part; not *P. gallinacea* De Vis, 1888.
***Leipoa gallinacea*** (De Vis, 1888); Boles, 2008: *Oryctos* 7: 204: in part, figure 6; not *P. gallinacea* De Vis, 1888.
***L.*** [***eipoa***] ***(Progura) gallinacea*** (De Vis, 1888); Boles, 2008: 204; not *P. gallinacea* De Vis, 1888.

**Figure 12.**
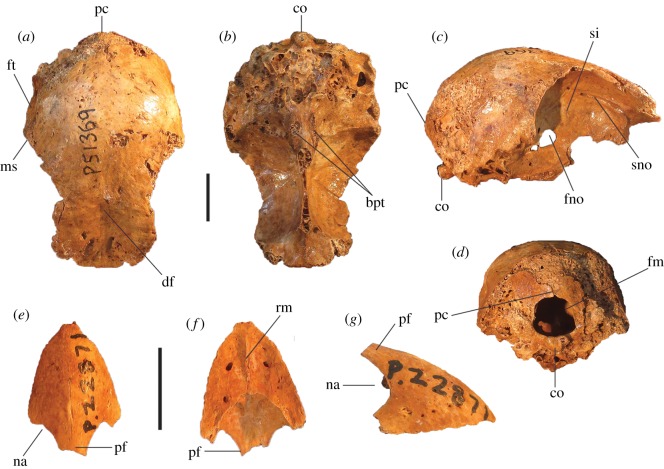
Cranial remains of *La. naracoortensis*; Cranium, SAM P.51369, in dorsal (*a*), ventral (*b*), lateral (*c*) and caudal (*d*) aspects; premaxilla, SAM P.22871, in dorsal (*e*), ventral (*f*) and lateral (*g*) aspects. bpt, processus basipterigoideus; co, condylus occipitalis; df, depressio frontalis; fm, foramen magnum; fno, foramen nervi optici; ft, fossa temporalis; ms, margo supraorbitalis; na, naris; pc, processus costalis; pf, processus frontalis; rm, rostrum maxillare; si, septum interorbitale; sno, sulcus nervi olfactorii. Scale bars, 10 mm.

**Figure 13.**
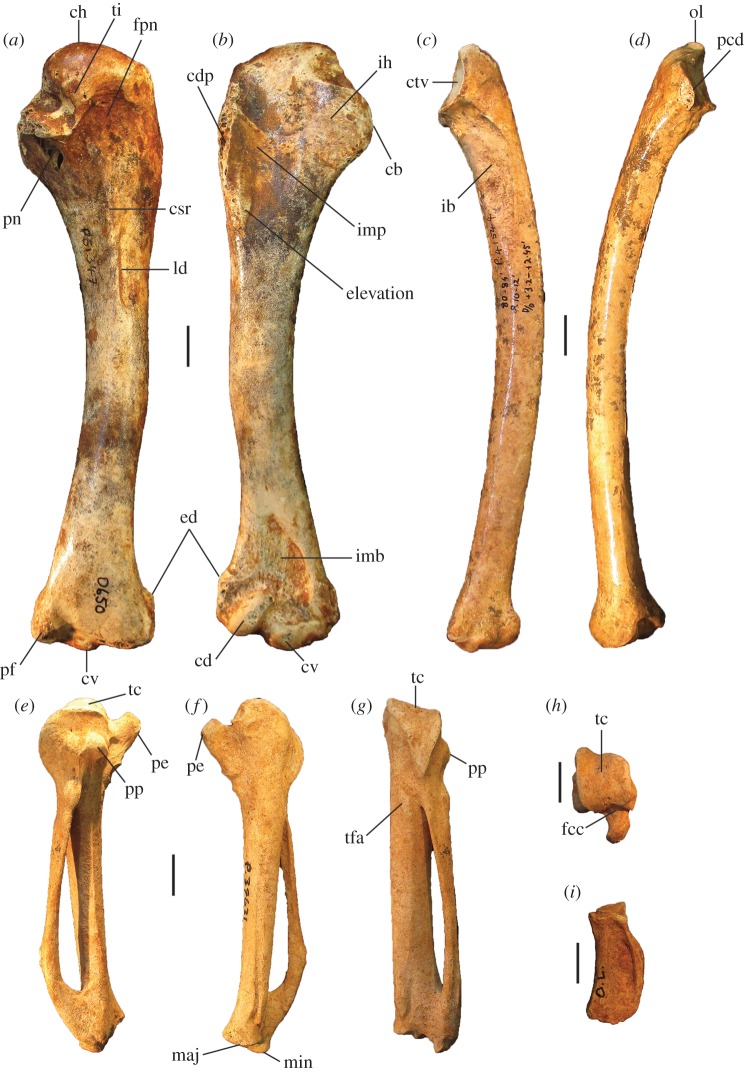
Wing elements of *La. naracoortensis*; Humerus, SAM P.51347, in caudal (*a*) and cranial (*b*) aspects; ulna, SAM P.41544, in ventral (*c*) and dorsal (*d*) aspects; carpometacarpus, SAM P.39631, in ventral (*e*), dorsal (*f*), caudal (*g*) and proximal (*h*) aspects; phalanx dig. major, SAM P.41844 (*i*). cb, crista bicipitalis; cd, condylus dorsalis; cdp, crista deltopectoralis; ch, caput humeri; csr, capital shaft ridge; ctv, cotyla ventralis; cv, condylus ventralis; ed, epicondylus dorsalis; fcc, fovea carpalis cranialis; fpn, fossa pneumotricipitalis dorsalis; ib, impressio brachialis; ih, intumescentia humeri; imp, impressio m. pectoralis; ld, attachment for m. latissimus dorsalis; maj, facies articularis digitalis major; min, facies articularis digitalis minor; ol, olecranon; pcd, processus cotylaris dorsalis; pe, processus extensorius; pf, processus flexorius; pn, foramen pneumaticum in the fossa pneumotricipitalis ventralis; pp, processus pisiformis; tc, trochlea carpalis; tfa, tuberosity for flexor attachment; ti, tuberculum intermedium. Scale bars, 10 mm.

**Figure 14.**
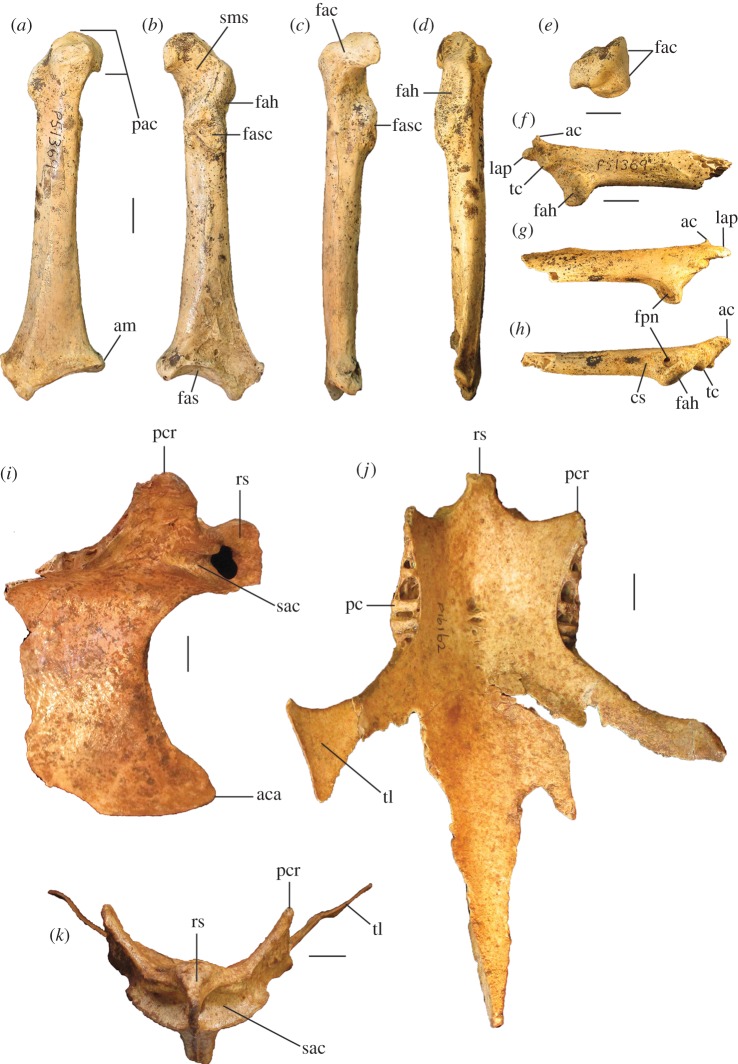
Pectoral and axial skeletal elements of *La. naracoortensis* Coracoid, SAM P.51369, in ventral (*a*), dorsal (*b*), medial (*c*) lateral (*d*) and omal (*e*) aspects; scapula, SAM P.51369, in lateral (*f*), medial (*g*) and ventral (*h*) aspects; sternum, SAM P.23125, in lateral (*i*) and cranial (*k*) aspects and SAM P.16162, in dorsal (*j*) aspect. ac, acromion; aca, apex carinae; am, angulus medialis; cs, collum scapulae; fac, facies articularis clavicularis; fah, facies articularis humeralis; fasc, facies articularis scapularis; fas, facies articularis sternalis; fpn, foramen pneumaticum; lap, crista lig. acrocoraco-procoracoideum; pac, processus acrocoracoideus; pc, processus costalis; pcr, processus craniolateralis; rs, rostrum sterni; sac, sulcus articularis coracoideus; sms, sulcus m. supracoracoidei; tc, tuberculum coracoideum; tl, trabecula lateralis. Scale bars, 10 mm.

**Figure 15.**
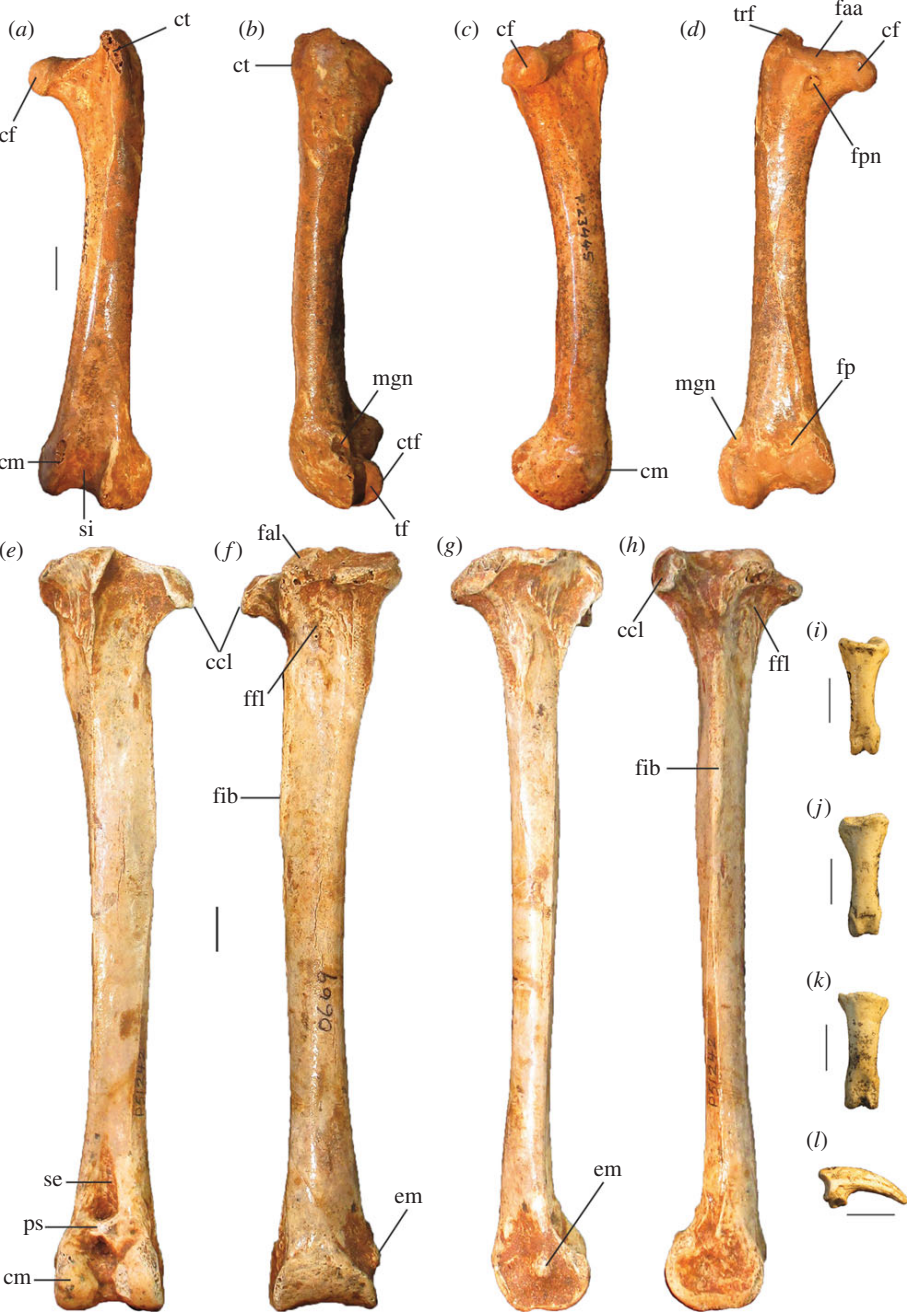
Leg and foot elements of *La. naracoortensis*; femur, SAM P23445, in cranial (*a*), lateral (*b*), medial (*c*) and caudal (*d*) aspects; tibiotarsus, SAM P51242, in cranial (*e*), caudal (*f*), medial (*g*) and lateral (*h*) aspects; phalanges, SAM P51369, dig. II.1 (*i*), dig. I.1 (*j*), dig. II.1 (*k*) and ungual (digit unknown) (*l*). ccl, crista cnemialis lateralis; cf, caput femoris; cm, condylus medialis; ct, crista trochanteris; ctf, crista tibiofibularis; em, epicondylus medialis; faa, facies articularis antitrochanterica; fal, facies articularis lateralis; ffl, fossa flexoria; fib, crista fibularis; fp, fossa poplitea; fpn, foramen pneumaticum; mgn, impression for m. gastrocnemialis lateralis; ps, pons supratendineus; se, sulcus extensorius; si, sulcus intercondylaris; tf, trochlea fibularis; trf, trochanter femoris. Scale bars, 10 mm.

**Figure 16.**
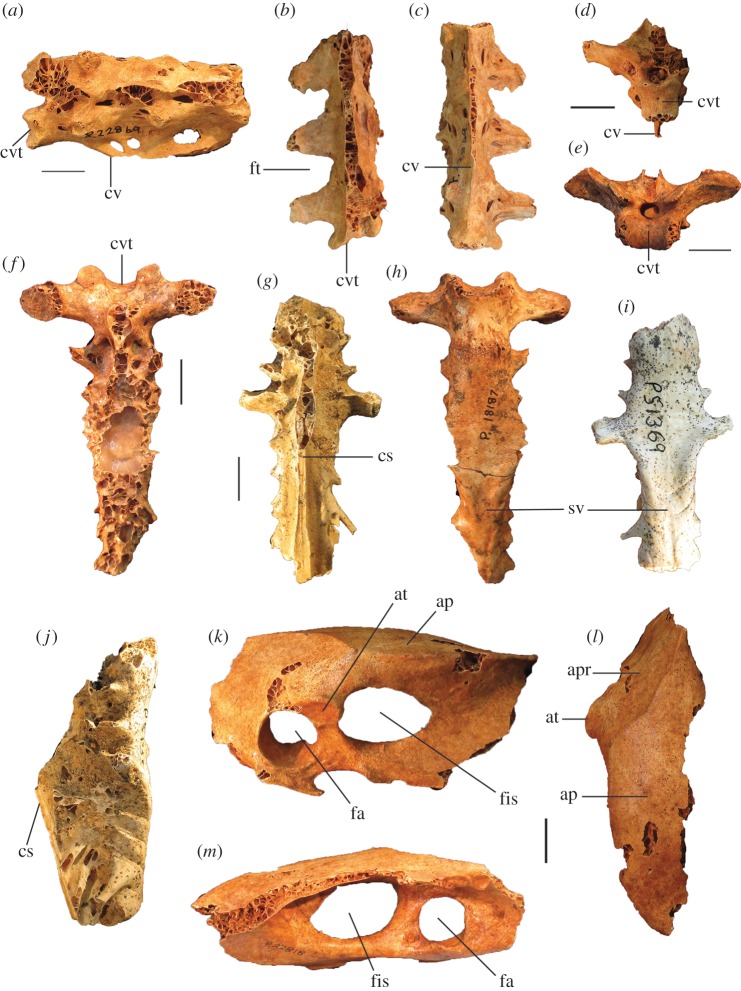
Pelvic and axial elements of *La. naracoortensis*; notarium, SAM P22869, in lateral (*a*), dorsal (*b*), ventral (*c*) and cranial (*d*) aspects; synsacrum, SAM P18187, in cranial aspect (*e*), SAM P18187 (*f*) and SAM P51369 (*g*) in dorsal aspect, SAM P18187 (*h*) and SAM P51369 (*i*) in ventral aspect and SAM P51369 in lateral (*j*) aspect; ilium, SAM P22818, in lateral (*k*), dorsal (*l*) and medial (*m*) aspects. ap, ala postacetabularis ilii; apr, ala preacetabularis ilii; at, antitrochanter; cs, crista spinosa synsacri; cv, crista ventralis; cvt, corpus vertebrae; fa, foramen acetabuli; fis, foramen ilioischiadicum; ft, fenestra transversaria; sv, sulcus ventralis synsacri. Scale bars, 10 mm.

**Holotype:** SAM P17856, nearly complete, slightly immature, R tarsometatarsus, missing trochlea metatarsi IV.

Material originally referred to *P. naracoortensis*: ***Gore Limestone Quarry, Darling Downs, southeast Queensland***—QM F2769, pL tarsometatarsus; ***Naracoorte Caves, southeastern South Australia: Main Fossil Chamber, Victoria Fossil Cave***—SAM P16700, R coracoid; ***Henschke's Fossil Cave***—SAM P17152, R tibiotarsus; SAM P17153, L humerus; SAM P17154, dL humerus; SAM P17857, pR femur; SAM P17876, dR tibiotarsus; SAM P17877, juvenile R ulna; SAM P17878, juvenile L humerus; SAM P17879, dL ulna; SAM P18181, cervical vertebra; SAM P18182, dL ulna; SAM P18183—pR and dR humerus; SAM P18184, L radius; SAM P18185, pR tarsometatarsus; SAM P18186, dR femur; SAM P18187, cranial fragment of a synsacrum. We now explicitly refer all these specimens to *Latagallina naracoortensis*.

**Newly referred material:** The following specimens were previously referred to *P. gallinacea* by van Tets [23]: ***Wombeyan Quarry, near Wombeyan Caves Reserve, New South Wales—***AM F54720, R coracoid; AM F54721, pR ulna; AM F54722, dR ulna; AM F54724, pL tarsometatarsus; AM F54725, dL tarsometatarsus; AM F54726, dR tarsometatarsus.

**Newly identified material:** ***Kilsby's Hole, Mt Gambier, South Australia***—SAM P42079, pR ulna; SAM P42733, four pedal phalanges; ***Big Sink, Wellington Caves, New South Wales***—NMNZ S46393, dL tarsometatarsus; ***Naracoorte Caves, South Australia***—further to the holotype and referred specimens from Naracoorte listed above, we refer nearly 500 additional whole or partial specimens in the collection of the South Australian Museum (includes six crania, three rostra, seven free vertebrae, three notaria, 16 synsacra, three ilia, 62 humeri, 46 ulnae, 14 radii, 33 carpometacarpi, 25 scapulae, 56 coracoids, three furculae, 15 sterna, 46 femora, 57 tibiotarsi, 67 tarsometatarsi; see appendix B) from various caves in the Naracoorte region, which were excavated subsequent to van Tets' description of *P. naracoortensis* [23]; and we also refer previously unregistered fossils from Buckridge Cave that were used in Boles' analysis and were included in his synonymy of *P. gallinacea* and ‘*Progura’ naracoortensis* [24]. There is evidence for only one large megapode in the Naracoorte region. All major skeletal elements are represented among the Naracoorte material, with most fossils being isolated elements, although three partial associated skeletons are known (***Big Bird Cave***—SAM P51368; ***Komatsu Cave***—SAM P51369 and ***Henschke's Fossil Cave***—SAM P51370).

**Type locality:** Henschke's Fossil Cave, near Naracoorte, South Australia [23] (figure 2).

**Stratigraphy and age:** The holotype is of Pleistocene age (probably Middle or Late Pleistocene) [37]. Most referred material comes from Pleistocene-aged cave deposits in the Naracoorte area (Henschke's Fossil Cave, Victoria Fossil Cave, Buckridge Cave, Big Bird Cave and Komatsu Cave) and no caves here are known to have pre-Middle Pleistocene faunas [29]. A small number of specimens come from deposits in other caves of uncertain age but which are presumed also to have a similar age range (Wombat Cave and Comaum Forest Cave near Naracoorte, and Kilsby's Hole, near Mt Gambier, approx. 100 km south of Naracoorte). Referred material from other locations (a proximal tarsometatarsus from Gore Limestone Quarry, southeast Queensland and all bones from Wombeyan Quarry and Wellington Caves, New South Wales) are also presumed to be of Pleistocene age.

**Revised diagnosis:** A large species of megapode with diagnostic features of the tarsometatarsus and femur as noted for the genus, and further distinguished from other members of the genus by the following features: **Tarsometatarsi:** (i) These are large, ranging in length from 89.0 to 105.6 mm. (ii) The fossa infracotylaris is moderately deep. (iii) The medial hypotarsal ridge is dorsoplantarly very deep in proximal aspect. (iv) The tuberositas m. tibialis cranialis is long and very protuberant, and the sulcus extensorius is shallow distally, such that the tuberositas is not recessed within the sulcus and is visible above the shaft in lateral and medial aspects. (v) In distal aspect, trochlea metatarsi IV is more plantarly depressed than trochlea metatarsi III. **Femora:** (i) The proximocranial surface adjacent to the crista trochanteris is relatively flat, lacking a fossa or with a fossa only very weakly developed.

**Differential diagnosis:** As well as differing from other taxa as per the generic diagnosis for *Latagallina*, tarsometatarsi of *La. naracoortensis* differ in size and proportion from extant and extinct species. Tarsometatarsi of *L. ocellata* are approximately 25% shorter than those of *La. naracoortensis*. Shorter tarsometatarsi of *La. naracoortensis* are within the length range of some extant species of megapode (*Al. lathami*, mean length 92.0 mm; *T. jobiensis*, 90.1 mm; *Ae. arfakianus*, 96.8 mm), but these species are differentiated from *La. naracoortensis* by being much smaller in all width values and therefore being proportionally much more gracile (appendix A). Tarsometatarsi of *La. naracoortensis* are only approximately two-thirds of the estimated length of tarsometatarsi of *P. gallinacea*, yet the proximal and distal widths of both species are similar, revealing the more robust nature of *La. naracoortensis*. Tarsometatarsi of *La. naracoortensis* are within the length range of *P. campestris*, but are more robust, with the longest tarsometatarsus of *La. naracoortensis* (SAM P51231) being 2 mm shorter than the holotype tarsometatarsus of *P. campestris*, yet having a proximal width 2 mm (9%) wider, and a distal width 2.4 mm (13%) wider. The greater distal width seen in tarsometatarsi of *La. naracoortensis* is related to this species having wider individual trochlea, and also to having a wider incisura between trochleae III and IV than in *P. campestris*, which is evident in distal aspect (cf. figure 5*v*,*w*). The holotypic femur of *P. campestris* falls within the length range of femora of *La. naracoortensis*, but they differ by anatomical features as noted in the generic diagnoses, and are further distinguished by proportional differences: the holotypic femur of *P. campestris* is 96.2% the mean length, 92.9% of the mean proximal width, 96.7% the mean distal width, yet 107.7% of the mean shaft width, of *La. naracoortensis*, thus *P. campestris* is characterized by having a proportionally more robust femur.

**Description and comparisons:** **Cranial bones:** Several crania are preserved but all are in poor condition. No mandibles or quadrates are preserved. The tips of three premaxillae are preserved, showing that the ventral symphyseal zone is 7.8–8.2 mm long, thus shorter than in *P. campestris* sp. nov. (greater than 9 mm), and that the tip of the bill in *La. naracoortensis* was relatively short and wide (figures 7*j*–*l* and 12*e*–*g*). The cranium (figure 12*a*–*d*) is of similar width and depth to that of extant *Al. lathami* and extinct *P. campestris*, and is therefore considerably larger than those of *L. ocellata* and *M. reinwardt*, but it has a relatively short, domed profile similar to that of *L. ocellata.* Minimum interorbital width of the frontals (18.7 mm) is similar to *P. campestris*, but neither the lacrimals nor the margo supraorbitalis in the posterior part of the orbits appear to flare laterally to the same extent as they do in *P. campestris*, and so *La. naracoortensis* apparently lacked the same degree of bony protection of the eyes. Unlike *L. ocellata*, *Al. lathami*, *M. reinwardt* and *Ma. maleo*, but similar to *P. campestris*, the septum interorbitale has a small foramen nervi olfactorii posterior to the sulcus nervi olfactorii (figure 12*c*). Measurements (mm): (SAM P41535), maximum depth, 26.6; length from the posterior of the cranium to the anterior of frontals, 41.9.

**Humerus:** Humeri of *La. naracoortensis* (figure 13*a*,*b*) are very large and robust, with deep muscle attachment scars, are well pneumatized and are distinguished from those of other species of megapode as follows. They are much larger in all dimensions than the humeri of any extant megapode, being around 50% longer than in *L. ocellata*, which has the longest humerus among extant species. It is further distinguished from extant megapodes by having: a tuberculum dorsale that is more swollen than in other species; a tuberculum intermedium that is not well pronounced, as in *T. fuscirostris* (prominent in *L. ocellata*, *Al. lathami* and less so in species of *Megapodius*); a tuberculum ventrale that projects caudally beyond the caput in proximal aspect (does not protrude like this in *L. ocellata*, *Al. lathami*, *T. fuscirostris* or *M. reinwardt*); a crista bicipitalis that is long as in species of *Leipoa*, *Alectura* and *Talegalla*, rather than short as in those of *Megapodius* and *Macrocephalon*; a crista deltopectoralis with strong cranial projection as in species of *Leipoa*, *Talegalla* and *Megapodius* (shallow projection in *Al. lathami*), with the adjacent scar for m. pectoralis on the cranial surface being strongly excavated (an elevated crescent of bone is seen here in species of *Leipoa*, *Alectura* and *Talegalla*); a processus flexorius that is blunter in shape in caudal aspect, as in *Ma. maleo* (more acute in species of *Leipoa*, *Alectura*, *Talegalla* and *Megapodius*) and a wider, deeper, more sharply demarcated impression for the m. brachialis.

The humerus of *La. naracoortensis* is only a little larger than that of *P. campestris*, and is distinguished from it by the following features: the tuberculum ventrale projects further caudally than the caput humeri in proximal aspect; the shaft is wider in caudal aspect; the capital shaft ridge is less elevated approaching the proximal end; the crista deltopectoralis is thicker ventrodorsally at its base, and the adjacent impression for m. pectoralis on the cranial surface is elevated adjacent to the crista, whereas this area is deeply excavated in *P. campestris*; the distal end is wider; the condylus dorsalis is longer and orientated more towards the long axis of the shaft; the condylus ventralis is oblong rather than spherical in cranial aspect, and does not protrude as far distally beyond the condylus dorsalis; the epicondylaris ventralis is proportionally larger; and the epicondylus dorsalis does not protrude as far dorsally. Measurements: for TL, PW, SW and DW, see table 4.

**Table 4.** Summary statistics for humeri of *La. naracoortensis* specimens from the Naracoorte Caves; TL, total length; PW, proximal width; SW, midshaft width; DW, distal width; measurements in mm. Measured specimens and source data in appendix B.

	TL	PW	SW	DW
mean	150.6	38.1	15.5	30.7
s.d.	0.92	1.70	0.56	0.45
CV (%)	0.61	4.45	3.59	1.47
minimum	135.5	33.6	13.8	27.2
maximum	151.2	40.3	16.1	32.7
*n*	10	20	20	27

**Ulna:** The ulna is larger and more robust than in any other species of megapode, extinct or extant (although it is of similar size to a distal ulna tentatively referred to *P. gallinacea*, see description for that species). In addition to its somewhat larger size, it is distinguished from the ulna of *P. campestris* by having a shaft that is straighter in dorsal aspect, a shallower impression for the m. brachialis, a less dorsoventrally compressed shaft and an olecranon that is more aligned with the long axis of the shaft in caudal aspect. Measurements: for TL, PW, SW and DW, see table 5.

**Table 5.** Measurements (mm) of ulnae of *La. naracoortensis*; summary statistics are given for specimens from Naracoorte (measured specimens and source data in appendix B); actual measurements are given for specimens from Wombeyan Quarry; TL, total length; PW, proximal width; SW, midshaft width; DW, distal width.

	TL	PW	SW	DW
Naracoorte Caves				
mean	154.2	18.7	10.9	19.5
s.d.	8.68	1.33	0.79	0.06
CV (%)	5.63	7.13	7.21	5.46
minimum	144.8	16.2	9.4	17.7
maximum	173.9	22.6	12.6	21.1
*n*	11	23	22	22
Wombeyan Quarry				
AM F54722	—	—	—	22.1
AM F54721	—	20.7	—	—

**Carpometacarpus:** Carpometacarpi of *La. naracoortensis* are much larger than those of any extant megapode (mean length 79.3 mm, versus 52.8 mm in *L. ocellata*). Carpometacarpi of *La. naracoortensis* are on average slightly longer than specimens of *P. campestris*, but their length ranges overlap (tables 2 and 6). In specimens of *La. naracoortensis* (SAM P39628; SAM P39631) that are of similar length to the holotype carpometacarpus of *P. campestris*, however, the maximum width of the carpometacarpus, measured at the widest arch of the os metacarpale minus, is approximately 2.5–3.5 mm wider, thus the spatium intermetacarpale is relatively wider. In addition, carpometacarpi of *La. naracoortensis* are distinguished from those of *P. campestris* by having a single fused tuberosity for the flexor attachment that is entirely proximal to the spatium intermetacarpale (two scars in *P. campestris*, the proximal one within the spatium intermetacarpale), and a deeper fovea carpalis cranialis. Carpometacarpi of *La. naracoortensis* are smaller than those of *P. gallinacea*, and are further distinguished by having: a dorsal rim of the trochlea carpalis that is rounded proximally rather than angular; a processus extensorius that is proportionally longer but less thick dorsoventrally (less than half the thickness of the adjacent carpal trochlea in *La. naracoortensis*, whereas in *P. gallinacea*, it is more than half the thickness of the trochlea); a single tuberosity for the flexor attachment that is entirely proximal to the spatium intermetacarpale (two distinct scars in *P. gallinacea*, the distal one mostly within the spatium intermetacarpale) and a less distally extended facies articularis digiti minor.

**Table 6.** Summary statistics for carpometacarpi of *La. naracoortensis* from the Naracoorte Caves; TL, total length; PW, proximal width; MW, metacarpal width, measured at widest arc of os metacarpale minus; DW, distal width; measurements in mm; measured specimens and source data in appendix B.

	TL	PW	MW	DW
mean	79.3	22.5	18.9	15.2
s.d.	4.86	1.76	1.45	1.08
CV (%)	6.13	7.81	7.67	7.13
minimum	73.4	19.8	16.6	13.6
maximum	90.8	25.8	20.5	17.0
*n*	25	27	5	26

**Coracoid:** Coracoids of this species (figure 13*a*–*e*) are very large and are further distinguished from extant species by the following features: the shaft is straight and robust and does not taper much towards the omal end (narrower and more tapered in *Al. lathami*, *T. fuscirostris* and *M. reinwardt*, and arched slightly laterally in *T. fuscirostris*); the facies articularis scapularis is broad, flat to convex and well defined, with a long axis at approximately 45° to the long axis of the shaft (the facies is convex with less distinct edges in *Al. lathami*, *T. fuscirostris* and *M. reinwardt*); the sulcus m. supracoracoidei is deep and is enclosed by the overhanging facies articularis clavicularis ventrally (in *Al. lathami* and *T. fuscirostris*, the sulcus is shallow due to minimal overhang of the facies artic. clavicularis); the processus acrocoracoideus is proportionally long, and its apex is more or less in line with the medial facies of the shaft in ventral aspect as in *T. fuscirostris* and *M. reinwardt* (proportionally shorter in all other taxa, and the apex placed further medially in *Al. lathami* and *L. ocellata*); the facies articularis clavicularis is medially flattened with flat sternal margin in medial aspect, as in *Al. lathami*, *T. fuscirostris* and *L. ocellata*, rather than notched as in *M. reinwardt*; the dorsal part of the facies articularis clavicularis projects a little beyond the medial margin of the shaft in dorsal view, as in *T. fuscirostris* and *Al. lathami* and *M. reinwardt*, unlike the far greater projection in *L. ocellata*; the ventral side of the facies articularis clavicularis does not protrude medially of the shaft; the sterno-ventral facies is flat in sternal aspect (convex in *Al. lathami*, *L. ocellata* and *T. fuscirostris*) and the distal margin of the processus lateralis is short relative to the length of the facies articularis sternalis (of about equal length in *M. reinwardt*).

Mature specimens are intermediate in size between the coracoids of the two extinct species of *Progura* described above. They are distinguished from *P. gallinacea* by their somewhat smaller size, and the following features of the omal end (the sternal coracoid of *P. gallinacea* is unknown): the ventrolateral margin of the facies articularis humeralis does not project as strongly laterally, and is more square (not as rounded) in ventral aspect; there is a shallower sulcus on the ventral surface between the ventrolateral margin of the facies articularis humeralis and the processus acrocoracoideus, resulting in a greater thickness of bone separating the ventral and dorsal surfaces here in omal aspect; and the facies articularis humeralis to the facies articularis scapularis is flatter (less concave). Coracoids of *La. naracoortensis* are differentiated from *P. campestris* by their larger size (mean length, 92.5 mm; 79.0 mm in the referred specimen of *P. campestris*), and the following morphological features: the facies articularis clavicularis has a long, medially flattened surface whose profile is triangular in medial aspect, and forms a distal hook that overhangs the sulcus dorsally (facies is short with a linear profile in *P. campestris*, and is less hooked); the ventromedial portion of the facies articularis clavicularis is not prominent medially in ventral aspect, whereas in *P. campestris*, it projects strongly; the dorsal portion of the facies articularis clavicularis does not project much further medially than the facies articularis scapularis in dorsal aspect (projects strongly in *P. campestris*); at the sternal end, the angulus medialis projects medially, whereas in *P. campestris*, it projects less and is smoothly contiguous with the medial shaft; the facies articularis sternalis has a large surface area dorsally and ventrally, and its sternal margin is deeply arched, providing a long, deep articulation with the sulcus artic. coracoideus of the sternum (shorter, shallower and straighter in *P. campestris*. Measurements: see table 7.

**Table 7.** Measurements (mm) of coracoids of *La. naracoortensis*; summary statistics are given for specimens from Naracoorte (individual measurements are in appendix B); actual measurement given for the specimen from Wombeyan Quarry; TL, medial length; OW, omal width; SW, midshaft width; StW, sternal width.

	TL	OW	SW	StW
Naracoorte Caves				
mean	92.5	21.8	11.0	28.7
s.d.	1.06	1.19	0.28	1.63
CV (%)	1.15	5.46	2.57	5.68
minimum	81.9	15.5	9.6	27.5
maximum	93.2	22.8	12.4	32.0
*n*	16	20	19	7
Wombeyan Quarry				
AM F54720	88.9	—	—	—

**Scapula:** The scapulae (figure 14*f*,*h*) are somewhat smaller and less robust than that of *P. gallinacea*, and are further distinguished from that species by having a convex dorso-lateral surface immediately distal of the acromion and dorsal to the facies articularis humeralis (*P. gallinacea* is grooved here). They are a little larger than scapulae of *P. campestris* and are further distinguished by having the following features: the pneumatic fossa on the ventral surface immediately distal of the facies articularis humeralis is larger; the acromion is more cranially extended; there is a large, costally directed process for attachment of the ligamentum acrocoraco-procoracoideum, which with the dorsal prominence on the acromion gives the proximal end a forked appearance (the dorsal process is much reduced in *P. campestris* and lacks this forked profile) and the dorso-lateral surface immediately distal of the acromion, and dorsal to the facies articularis humeralis, is convex rather than grooved, with the dorsal margin broadly rounded, not compressed into a narrow margin as in *P. campestris*. Measurements: see table 8.

**Table 8.** Summary statistics for scapulae of *La. naracoortensis* specimens from Naracoorte Caves (individual measurements are in appendix 2); TL, total length; WA, width of facies artic. humeralis; WC, width of collum scapulae, measured immediately distal of the facies artic. humeralis; DC, depth of collum scapulae, measured immediately distal of the facies artic. humeralis; LAA, length from the distal rim of facies artic. humeralis to the tip of acromion; measurements in mm.

	TL	WA	WC	DC	LAA
mean	—	12.3	10.8	7.6	24.1
s.d.	—	0.85	0.63	0.53	0.90
CV (%)	—	6.92	5.86	6.93	3.74
minimum	—	10.3	9.7	6.7	22.6
maximum	>100	13.7	12.0	8.5	25.8
*n*	1	22	23	20	18

**Sternum:** The best-preserved specimen (SAM P16162) preserves almost the entire length of the sternum (figure 14*j*,*k*). It is 138.7 mm long, and has a cranial width of 44.5 mm, measured across the proc. craniolaterales. The depth of the carina is variable between individuals, with the largest sternum measuring 96.3 mm in carina depth, and the smallest 66.2 mm. Despite differences in carina depth, the sulci articularis coracoidei are consistently wide and deep in all individuals. The tip of the keel is ventrocranially recurved (figure 14*i*) as in extant megapodes and the extinct *P. campestris*. The deep carina of *La. naracoortensis*, along with other pectoral elements that show no signs of reduction, are consistent with adults of this species retaining the ability to fly, despite their large size.

**Pelvis:** Only fragmentary remains of the pelvis are preserved. The best-preserved specimen (SAM P22818; figure 16*k,l,m*) is an approximately 80 mm long fragment of a left ala postacetabularis ilii and ala ischia, preserving the foramen acetabuli, antitrochanter and foramen ilioischiadicum. The specimen does not preserve enough of the anatomy to allow a meaningful comparison with other species, but indicates that this species had a very large pelvis as would be expected.

**Femur:** Femora of *La. naracoortensis* (figure 15*a*–*d*) have features as follows. The mean femur length in this species is greater than the mean tarsometatarsus length, unlike *P. campestris* in which femur and tarsometatarsus are of equal length. The proximocranial surface adjacent to the crista trochanteris is relatively flat, and it lacks a deep pneumatized fossa (e.g. SAM P51368) as seen in some other taxa. Though a fossa is lacking, pneumatism of this area is seen in some individuals (e.g. SAM P51262, SAM P51263). At the junction between the crista trochanteris, the cranial surface and the facies articularis antitrochanterica, the bone is compressed into a thin crest, as in *L. ocellata* and *M. reinwardt*. In proximal aspect, the cranial edge of the facies artic. antitrochanterica forms a straight line between the crista trochanteris and the caput femoris, as in all taxa examined except for *Al. lathami* and *P. campestris*, in which it is concave. On the proximocaudal surface, there is a large, round pneumatic foramen adjacent to the caput femoris (see generic diagnosis). This feature can be seen in fig. 4*f* of Van Tets [23] and figs 2(9) and 5 of Boles [24], but has not previously been noted or identified as a distinguishing character. However, we identify it as an autapomorphy that defines the genus *Latagallina* (see generic diagnosis above, and phylogenetics results below). Distally, the rims of the cristae patellaris medialis and lateralis are broad, deep and long, and enclose a narrower, more v-shaped sulcus patellaris than in all extant species examined and *P. campestris*. The epicondylus lateralis flares more widely laterally than in all other species examined, and in caudal aspect, this forms a buttress of bone laterad of the impression of the m. gastrocnemialis lateralis, which is absent in all other species. The crista tibiofibularis is proximodistally shorter but more caudally projecting than in similarly sized *P. campestris*. Measurements (mm): for TL, PW, SW and DW, see table 9; mean minimum shaft circumference, 41.2.

**Table 9.** Summary statistics for femora of *La. naracoortensis* specimens from the Naracoorte Caves (individual measurements are in appendix 2); TL, total length; PW, proximal width; SW, midshaft width; DW, distal width; measurements in mm.

	TL	PW	SW	DW
mean	110.8	29.6	13.0	27.1
s.d.	5.63	1.39	1.63	1.48
CV (%)	5.08	4.69	6.10	5.47
minimum	102.4	27.8	11.8	25.2
maximum	119.4	31.7	14.1	29.6
*n*	6	10	12	12

**Tibiotarsus:** Tibiotarsi of this species (figure 15*e*–*h*) are very large and robust. They are of similar length to the tibiotarsus in the holotype of *P. campestris*, but have more robust proximal and distal ends. Proximally, the crista cnemialis lateralis is as proportionally wide as in *P. campestris*, *M. reinwardt* and *L. ocellata*, unlike *T. fuscirostris*, *Al. lathami* and *Ma. maleo*, in which this crista has less lateral extent. The proximal articular surface has a large surface area, and the incisura tibialis is wide, creating a greater separation between the facies articularis lateralis and the crista cnemialis lateralis, unlike *P. campestris* in which these are closer together. Distally, the epicondylus medialis is protuberant beyond the rim of the condylus medialis in cranial aspect, distinguishing it from *P. campestris*, *Ma. maleo* and *Ae. arfakianus*, in which the condylar rim occludes it. The proximodistal length of the pons supratendineus is much greater laterally than medially, and its distal margin is angled obliquely to the axis (rather more horizontal in *P. campestris*). The condylar width is relatively broad compared with the depth of the condylus medialis (subequal in *P. campestris*). Measurements (mm): for TL, PW, SW and DW, see table 10; mean width of the proximal end without the crista cnemialis lateralis, i.e. the articular surface only, 23.8; mean depth of the cotyla lateralis, 19.0; mean depth of the cotyla medialis, 19.7; mean minimum shaft circumference, 30.7.

**Table 10.** Summary statistics for tibiotarsi of *La. naracoortensis* specimens from the Naracoorte Caves; TL, total length; PW, proximal width; SW, midshaft width; DW, distal width; measurements in mm; measured specimens and source data in appendix 2.

	TL	PW	SW	DW
mean	157.5	23.8	10.9	21.0
s.d.	9.24	1.49	0.99	1.28
CV (%)	5.87	6.24	9.11	6.12
minimum	144.2	21.6	9.1	19.1
maximum	170.3	26.3	13.1	23.8
*n*	5	11	21	35

**Tarsometatarsus:** Diagnostic features are described above in the generic and species diagnoses and depicted in figure 5*c*,*h*,*m*,*r*,*w*. Measurements (mm): for TL, PW, SW and DW, see table 11; prox. depth including the hypotarsus, mean 22.2; depth of trochlea metatarsi II, mean 9.5; depth trochlea metatarsi III, mean 11.3; depth of trochlea metatarsi IV, mean 8.4.

**Table 11.** Summary statistics for tarsometatarsi of *La. naracoortensis* specimens from the Naracoorte Caves; TL, total length; PW, proximal width; SW, midshaft width; DW, distal width; measurements in mm; measured specimens and source data in appendix 2.

	TL	PW	SW	DW
mean	97.3	23.1	10.1	24.8
s.d.	4.53	1.44	0.65	1.37
CV (%)	4.66	6.23	6.44	5.53
minimum	89.0	19.7	9.2	22.7
maximum	105.6	25.9	11.3	28.0
*n*	19	25	22	22

**Phalanges:** Relatively few pedal phalanges of this species are preserved, and given the abundance of other skeletal material from the Naracoorte Caves, it seems likely that they have generally been overlooked in the collection or processing of fossils. Six phalanges (L and R I.2; L III.1; R IV.1; and two unguals from unknown digits) belonging to an individual (SAM P51368) are preserved from Big Bird Cave, Naracoorte. All are of similar size to the equivalent bones in *P. campestris* but differ slightly in proportion from that species as follows: phalanx I.2 is slightly flatter dorsoventrally; phalanx II.1 is a little wider proximally; and phalanx III.1 has a slightly wider shaft, a broader, more symmetrical proximal articular facet (lateral half of the facet is larger in *P. campestris*), and proximoplantar surface is much more deeply excavated. The unguals are short, lateromedially compressed and very deeply curved, and are not elongate or dorsoventrally flattened, unlike in specialized mound-building species such as *Al. lathami* and *L. ocellata*.

**Remarks on specimens previously referred to this species:** Two megapode fossils (QM F23258, a carpometacarpus; and QM F23259, a pL tarsometatarsus) from the early Pliocene Bluff Downs Local Fauna of northeastern Queensland were tentatively referred by Boles & Mackness [11] to *Latagallina* (as *Progura*) *naracoortensis* rather than to *P. gallinacea* because of their small size, but these authors commented that they could belong to a new species. Published measurements and photographs of these bones indicate that they are unlikely to belong to *La. naracoortensis*, although the proximal width of the tarsometatarsus (19.5 mm) is closest in size to that species (table 11). A photograph [11] of QM F23259 reveals the shaft of the tarsometatarsus to be very tapered towards the distal end. This is dissimilar to the stout shaft characteristic of species of *Latagallina*. The shaft is also more tapered than in *P. campestris*, but has a very similar shape to *Al. lathami* albeit a bit larger, or *P. gallinacea* albeit very much shorter*.* However, estimated total length of the bone if complete (approx. 90 mm) is only 60% of the length of the tarsometatarsus of *P. gallinacea* (approx. 147.5 mm), and is somewhat shorter than that of *P. campestris* (107 mm) and *La. naracoortensis* (mean 97.3 mm). This bone could be a large specimen of *Al. lathami*, the tarsometatarsus of which can reach at least 101.9 mm in length (MV R4288; PW = 17.1 mm), albeit one that is slightly more robust than modern specimens. The Bluff Downs fossils require further examination, and comparison with extant taxa and the extinct Plio-Pleistocene taxa that are known as a result of this study.

**Remarks:** *Latagallina naracoortensis* was a stout, robustly built Pleistocene megapode larger and heavier than any extant member of the family, but was somewhat smaller than *P. gallinacea.* Although it was of similar size and mass to *P. campestris* (see Body mass estimates), it had different body proportions (see species descriptions and Simpson log-ratio diagram), and fossils of these taxa are readily distinguishable based on morphology.

#### ***Latagallina olsoni*** Shute, Prideaux & Worthy, sp. nov.

##### (figures 5*d*,*i*,*n*,*s*,*x*; and 17–19)

**Zoobank ID:** urn:lsid:zoobank.org:act:961E913A-9DF1-4FD0-B757-10CD65809220

**Figure 17.**
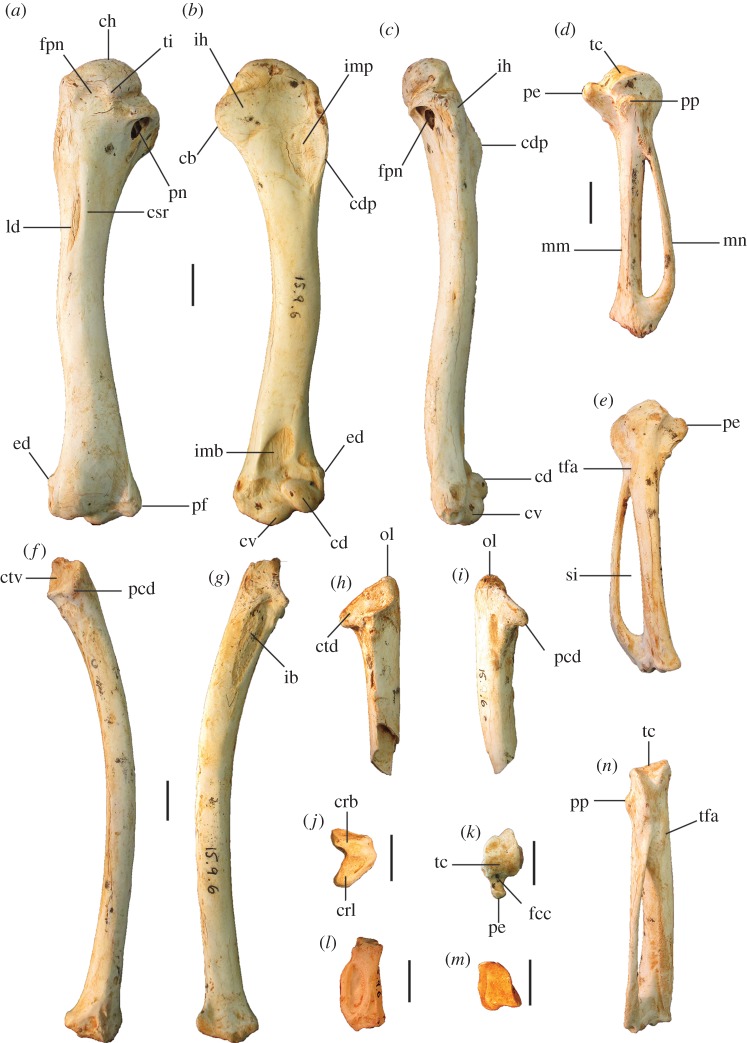
Wing bones of *La. olsoni* sp. nov., WAM 15.9.6, holotype. Humerus in caudal (*a*), cranial (*b*) and ventral (*c*) aspects; carpometacarpus in ventral (*d*), dorsal (*e*) caudal (*n*) and proximal (*k*) aspects; ulna (left) in dorsal (*f*) and ventral (*g*) aspects and proximal ulna (right) in caudal (*h*) and dorsal (*i*) aspects; os carpi ulnare (*j*); phalanx dig. major (*l*); os carpi radialis (*m*). cb, crista bicipitalis; cd, condylus dorsalis; cdp, crista deltopectoralis; ch, caput humeri; crb, crus breve; crl, crus longum; csr, capital shaft ridge; ctd, cotyla dorsalis; ctv, cotyla ventralis; cv, condylus ventralis; ed, epicondylus dorsalis; fcc, fovea carpalis cranialis; fpn, fossa pneumotricipitalis dorsalis; ib, impressio brachialis; ih, intumescentia humeri; imb, impressio musculo brachialis; imp, impressio musuclo pectoralis; it, incisura tendineus; ld, attachment for m. latissimus dorsalis; mm, os metacarpale majus; mn, os metacarpale minus; ol, olecranon; pcd, processus cotylaris dorsalis; fpn, foramen pneumaticum in fossa pneumotricipitalis ventralis; pe, processus extensorius; pf, processus flexorius; pn, foramen pneumaticum; pp, processus pisiformis; si, spatium intermetacarpale; tc, trochlea carpalis; tfa, tuberosity for flexor attachment; ti, tuberculum intermedium. Scale bars, 10 mm.

**Figure 18.**
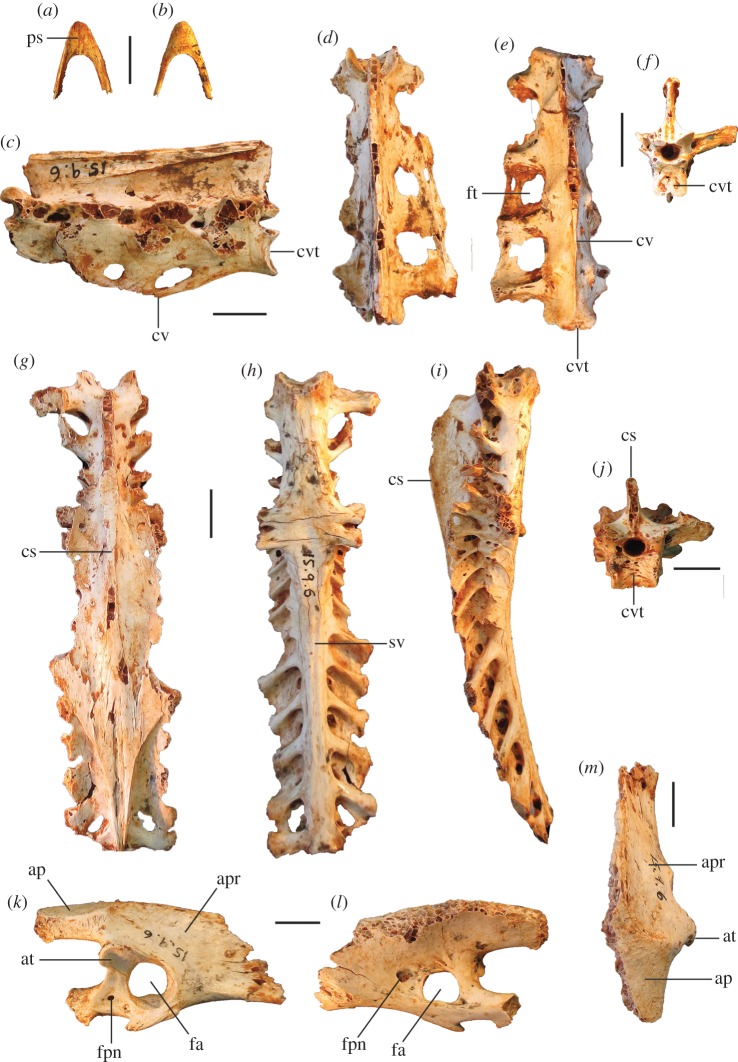
Cranial, axial and pelvic elements of *La. olsoni* sp. nov., WAM 15.9.6, holotype. Mandible tip in dorsal (*a*) and ventral (*b*) aspects; notarium in right lateral (*c*), dorsal (*d*), ventral (*e*) and cranial (*f*) aspects; synsacrum in dorsal (*g*), ventral (*h*), lateral (*i*) and cranial (*j*) aspects; ilium in right lateral (*k*), medial (*l*) and dorsal (*m*) aspects. ap, ala postacetabularis ilii; apr, ala preacetabularis ilii; at, antitrochanter; cs, crista spinosa synsacri; cv, crista ventralis; cvt, corpus vertebrae; fa, foramen acetabuli; fpn, foramen pneumaticum; ft, fenestra transversaria; ps, pars symphysialis; sv, sulcus ventralis synsacri. Scale bar, 10 mm.

**Figure 19.**
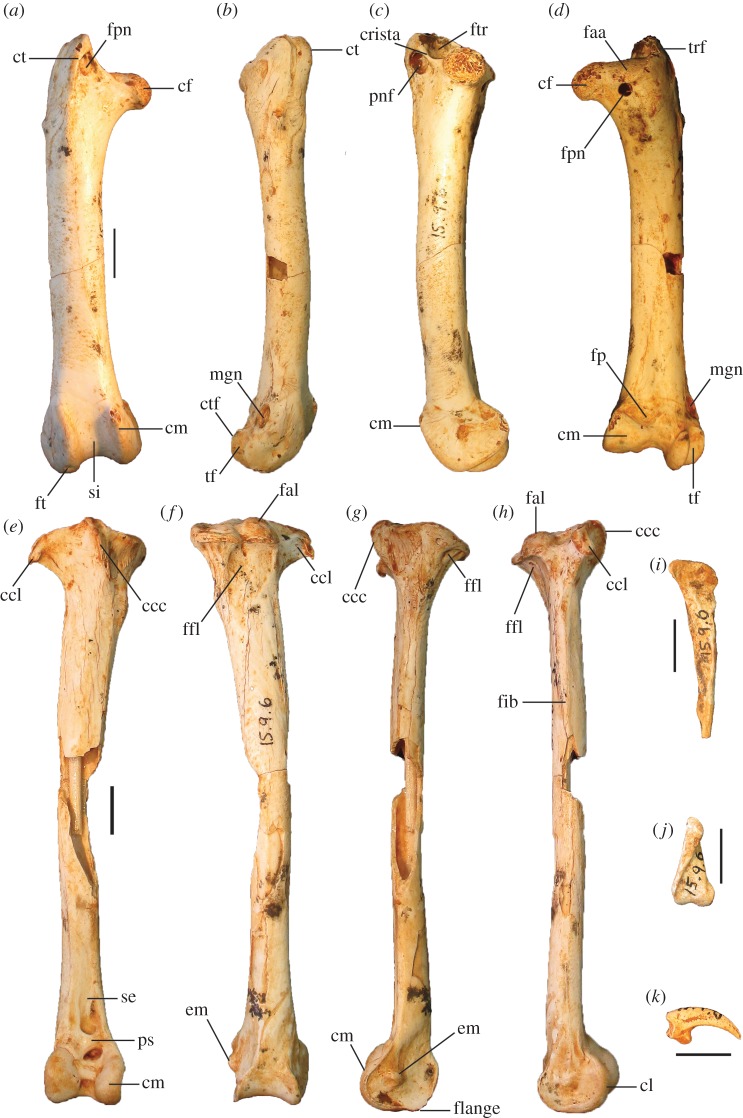
Leg and foot elements of *La. olsoni* sp. nov., WAM 15.9.6, holotype. Femur in cranial (*a*), lateral (*b*), medial (*c*) and caudal (*d*) aspects; tibiotarsus in cranial (*e*), caudal (*f*), lateral (*g*) and medial (*h*) aspects; fibula (*i*); phalanx dig. I.1 (*j*); ungual (digit unknown) (*k*). ccc, crista cnemialis cranialis; ccl, crista cnemialis lateralis; cf, caput femoris; cl, condylus lateralis; cm, condylus medialis; ct, crista trochanteris; ctf, crista tibiofibularis; em, epicondylus medialis; faa, facies articularis antitrochanterica; fal, facies articularis lateralis; ffl, fossa flexoria; fib, crista fibularis; fp, fossa poplitea; fpn, foramen pneumaticum; ft, fovea tendineus m. tibialis cranialis; ftr, fossa trochanteris; mgn, impression for m. gastrocnemialis lateralis; pnf, pneumatic fossa; ps, pons supratendineus; se, sulcus extensorius; si, sulcus intercondylaris; trf, trochanter femoris; tf, trochlea fibularis. Scale bar, 10 mm.

**Holotype** (figures 5*d*,*i*,*n*,*s* and 17–19): WAM 15.9.6, associated remains of one adult individual, comprising the following elements: mandible, approximately 1.5 cm of tip; two vertebrae, one cervical, one caudal; 11 ribs and rib fragments; pelvis, disarticulated—complete synsacrum, portions of L and R ilia; notarium; L, a fragment of pR humerus; pR, L ulna; L radius, missing proximal end; R carpometacarpus; L os carpi ulnare; R os carpi radiales; L phalanx II digiti majoris II.1; L and R phalanx digiti majoris II.2; R femur, L femur missing lateral condyle; tibiotarsi, R with damaged midshaft, shaft and distal fragment L; L fibula; R and L (minus trochlea metatarsi IV) tarsometatarsus; os metatarsale I; 1 ungual digit unknown. Number of individual bones = 40.

**Referred material:** WAM 15.9.3, complete L carpometacarpus; WAM 15.9.4, fragment of a pR femur. Including the holotype skeleton, number of individual bones = 42, minimum number of individuals = 3.

**Type locality:** Leaena's Breath Cave, Thylacoleo Caves, Nullarbor Plain, Western Australia (figure 2; see Key locations).

**Stratigraphy, age and fauna:** All material comes from Leaena's Breath Cave. The associated holotype skeleton was found lying on the surface of the sediment floor. Surface sediments from elsewhere in the cave have been dated to the Middle Pleistocene, and so the holotype may also be of this age. The referred femoral fragment (WAM 15.9.4) was excavated from a depth of 5–10 cm below the sediment surface, in stratigraphic Unit 1, Quadrat 1, Pit B (Middle Pleistocene [28]). The referred carpometacarpus (WAM 15.9.3) was excavated from 95 to 100 cm below the sediment floor, in stratigraphic Unit 3, Quadrat 2, Pit B (2.58–0.78 Ma, Early Pleistocene [28]).

**Diagnosis:** A species of *Latagallina* as defined by generic features of the tarsometatarsus and femur, and distinguished from other members of the genus by its small size and by the following unique combination of features. **Tarsometatarsus:** (i) The fossa infracotylaris and sulcus extensorius are very deep. (ii) The medial hypotarsal ridge is moderately deep in proximal aspect. (iii) The tuberositas m. tibialis cranialis is relatively short and not protuberant, and completely recessed within the sulcus extensorius. (iv) Trochlea metatarsi IV is only very slightly plantarly depressed relative to trochlea metatarsi III, as seen in distal aspect. **Femur:** (i) The proximocranial surface adjacent to the crista trochanteris has a deep pneumatic fossa.

**Differential diagnosis:** *Latagallina naracoortensis* is distinguished from the new species by its much larger size (tarsometatarsus approx. 38% longer on average), and by features as follows: tarsometatarsi of *La. naracoortensis* have: a shallower fossa infracotylaris and sulcus extensorius; a longer, much more protuberant tuberositas m. tibialis cranialis that is elevated above the shaft rather than recessed in the sulcus extensorius; a dorsoplantarly deeper medial hypotarsal ridge in proximal aspect; and with greater plantar depression of trochlea metatarsi IV relative to trochlea metatarsi III. Femora of *La. naracoortensis* are differentiated by their much larger size, and by lacking a fossa on the proximocranial surface adjacent to the crista trochanteris, the bone here being rather flat and pneumatization slight to absent, rather than excavated and well pneumatized as in *La. olsoni*.

**Measurements of holotype and referred material**: See table 12.

**Table 12.** Long-bone measurements (mm) of *La. olsoni* holotype and referred material; TL, total length; PW, proximal width; SW, midshaft width; DW, distal width.

element/side	catalogue no.	TL	PW	SW	DW
holotype					
humerus, L	WAM 15.9.6	113.7	26.6	11.5	22.4
ulna, L	WAM 15.9.6	∼116	13.7	8.7	14.9
carpometacarpus, R	WAM 15.9.6	61.5	17.7	—	12.8
phalanx dig. majoris I	WAM 15.9.6	22.7			
phalanx dig. majoris II	WAM 15.9.6	21.9			
femur, R	WAM 15.9.6	87.1^a^	21.4	10.8	20.0
tibiotarsus, R	WAM 15.9.6	117.1	16.8	—	15.0
tibiotarsus, L	WAM 15.9.6	—	—	8.2	—
tarsometatarsus, R	WAM 15.9.6	70.3	18.3	8.3	18.4
referred material					
carpometacarpus, L	WAM 15.9.3	63.0	19.7	14.1	11.2

^a^Total length of femur would probably be 1 or 2 mm longer if the crista trochanteris was not eroded.

**Etymology:** The species name honours Storrs Olson, of the Smithsonian National Museum of Natural History, Washington, DC, who has worked extensively on fossil avifaunas in various parts of the world, including Australia, and who was the first author to note that *P. gallinacea* and ‘*Progura’ naracoortensis* could represent species in different genera [26]. We honour him here with a new species in the same genus as the species from Naracoorte.

**Description and comparisons:** The holotype skeleton preserves most post-cranial elements, but lacks a skull. The bones of the tarsometatarsus are completely fused, and the surface of all bones lacks a porous texture, thus the holotype individual is an adult. Anatomical detail has excellent preservation. This species is considerably smaller than the other extinct Plio-Pleistocene species described herein. Its wing bones are longer and more robust than those of extant species, but its leg bones, while also robust, are of comparable size to those of extant species of megapode.

**Cranial material:** Only the tip of the mandible survives (figure 18*a*,*b*), with around 15 mm of the left and right rami preserved. Although most of the mandible is missing, the wide angle (approx. 30°) between the rami shows that the tip of the bill in this species was wider than in extant *L. ocellata* or *Al. lathami* (approx. 20° in both) and had a shorter symphyseal zone.

**Humerus:** The left humerus of the holotype (figure 17*a*–*c*) preserves the complete length of the bone, with the only damage being slight erosion of the crista deltopectoralis. It is somewhat longer than humeri of *L. ocellata*, which has the longest humerus among extant megapodes, but is also considerably more robust overall, having proportionally wider proximal and distal ends and a wider shaft. It is further distinguished from humeri of *L. ocellata* by having a shallower, more enclosed fossa pneumotricipitalis ventralis, a fossa pneumotricipitalis dorsalis that is barely marked (broad, shallow fossa present in *L. ocellata*), a more dorsally prominent tuberculum dorsale, a capital ridge that is less compressed, an impressio m. brachialis in the fossa brachialis that is wider for its length, a condylus dorsalis that is broader proximally (dorsal and ventral edges converge proximally in *L. ocellata*) and a straighter (i.e. less sigmoid) shaft in dorsal aspect. The humerus of the holotype skeleton is longer compared with the length of associated leg elements than in extant Australian megapodes (see Simpson log-ratio diagram). It is approximately 25% shorter than humeri of *La. naracoortensis*, and is further distinguished from its larger congener by having: a tuberculum ventrale that does not project caudally beyond the caput in proximal aspect; an intumescentia humeri that is less inflated; a more pronounced tuberculum intermedium; a proportionally shorter crista bicipitalis; no protuberance on the cranial surface where the crista deltopectoralis merges with the shaft distally (*La. naracoortensis* has a low protuberance here) and a more acute processus flexorius in caudal aspect (blunter in *La. naracoortensis*). Measurements: for TL, PW, SW and DW, see table 12.

**Ulna:** The left ulna in the holotype (figure 17*f*,*g*) has an eroded olecranon, but this is preserved on the contralateral side (figure 17*h*,*i*). A total length of approximately 116 mm is therefore estimated from the two bones of this individual. It is much smaller in all dimensions than the ulnae of *P. campestris* and *La. naracoortensis*. It is further distinguished from *P. campestris* by having a shallower impression for the m. brachialis, a shaft that is proportionally a little wider cranio-caudally, a condylus dorsalis whose caudal margin protrudes ventrally much more sharply from the shaft (merges more smoothly with the margo caudalis of the shaft in *P. campestris*). Though only around 75% of the length of the ulnae of *La. naracoortensis* and considerably more slender, *La. olsoni* shares with its larger relative a shallow fossa brachialis, a proximodistally short tuberculum carpale whose profile is concave in ventral aspect (more elongate proximodistally and convex in *L. ocellata*), and a ventrally projecting condylus dorsalis. Measurements: for TL, PW, SW and DW, see table 12.

**Radius:** The radius is missing the proximal third of its length, but the preserved portion shows that the facies articularis radiocarpalis slopes proximally towards the tip of the tuberculum aponeurosis ventralis rather than being at right angles to the axis as in *L. ocellata*.

**Carpometacarpus:** The holotype skeleton preserves a near complete right carpometacarpus, with slight erosion of the processus pisiformis and of the distal end (figure 17*d*,*e*,*n*). It is very similar to carpometacarpi of *L. ocellata*, but differs by its larger size (holotype carpometacarpus approx. 16.5% longer than those of *L. ocellata*) and by the tuberosity for the flexor attachment lying proximal to the spatium intermetacarpale (as in *La. naracoortensis*), not within it. The os metacarpale minus is broad and flattened proximally at the synostosis, and joins ventral to the ventral rim of the trochlea carpalis, which is aligned transversely to the shaft axis. The ventral rim of the trochlea carpalis is angled transversely at about a 30° angle to the long axis of the shaft in caudal aspect. Measurements: see table 12.

**Pelvis:** The entire length of the synsacrum is preserved (figure 18*g*–*j*), and is disarticulated from other portions of the pelvis. It comprises 15 synostosed vertebrae, as in *L. ocellata*. Portions of the left and right ilia are preserved (right ilium pictured in figure 18*k*–*m*), but both lack most of the postacetabular section, the ischia and the pubes. The preserved portions reveal the pelvis to be much smaller than those of *La. naracoortensis.* The pelvis is pneumatic with foramina entering the corpus ischii both internally and externally (as in *Leipoa*), but there is also a pneumatic opening on the ala preacetabularis ilii anterior to the acetabulum on the internal surface (figure 18*l*), which is absent in *L. ocellata* and *M. reinwardt*, and is present but smaller and less distinct in *Al. lathami* and *T. fuscirostris*. The synsacrum is deflected slightly ventrally in its caudal half (figure 18*i*), but less so than in *L. ocellata*, thus the ventral profile is flatter in lateral aspect. It is also longer and broader than in *L. ocellata*, despite the two species having a similar leg length (sum of length of femur, tibiotarsus and tarsometatarsus in holotype of *La. olsoni* approx. 274 mm, versus approx. 277 mm in *L. ocellata*, e.g. SAM B11483), thus *La. olsoni* was a larger-bodied and overall more robust species. The length of the synsacrum is almost identical to that of *T. fuscirostris*, but the synsacrum is deeper in *La. olsoni*, again suggesting larger overall body size. Measurements (mm): length of synsacrum, 98.5; depth measured at the level of the foramen acetabuli, 26.9.

**Vertebrae:** Most of the notarium is preserved in the holotype (figure 18*c*–*f*), with loss of the processus transversi on the left side. It includes four vertebrae, as in most megapodes [21], although the centrum of the first is incompletely ankylosed. It is larger than the notarium of *L. ocellata*, as expected from its larger synsacrum and more robust leg bones*.* Measurements (mm): length, 49.2.

**Femur:** The holotypic femora (figure 19*a*–*d*) are of similar length to those of extant *T. fuscirostris*, *L. ocellata* and *Ma. maleo* and are slightly shorter than in *Al. lathami*, but are considerably more robust than in any extant species. This is consistent with *La. olsoni* being more massive than extant megapodes (see Body mass estimates). However, the femora are much smaller than those of extinct *P. campestris* and *La. naracoortensis.* As per the generic diagnosis, femora of *La. olsoni* have a large, round pneumatic foramen on the caudal surface adjacent to the facies articularis antitrochanterica (figure 19*d*), which further distinguishes it from all extant Australian megapodes, and from *P. campestris*. The crista trochanteris is proportionally longer than in all species apart from *Ma. maleo* and *L. ocellata*, but in cranial aspect, its medial edge is angled less medially, and so more proximally, than in those species. There is a well-marked fossa trochanteris. The proximocranial surface adjacent to the crista trochanteris has a deep, pneumatic fossa as in most other taxa (figure 19*a*,*c*), but distinct from its congener *La. naracoortensis* in which this fossa is absent or very weakly developed. The cranial margin of the fossa trochanteris comprises a thin crest (figure 19*c*), as in *L. ocellata*, *M. reinwardt* and *La. naracoortensis*, and distinct from *T. fuscirostris*, *Al. lathami* and *P. campestris*, where it is thicker. Distally, the fossa poplitea is enlarged proximomedially adjacent to the crista supracondylaris medialis, and is deeper than in all other species examined. The crista tibiofibularis projects proportionally further caudally relative to the trochlea fibularis than in all species examined apart from *La. naracoortensis*. Measurements (mm): for TL, PW, SW and DW, see table 12; prox. depth, 17.9; minimum shaft circumference, 32.4.

**Tibiotarsus:** The right tibiotarsus of the holotype (figure 19*e*–*h*) preserves the complete length and both proximal and distal ends, with some damage to the shaft. It approaches the length of tibiotarsi of *L. ocellata*, *M. reinwardt* and *T. fuscirostris*, but it is much more robust, consistent with *La. olsoni* being a stouter and more heavily built species overall (see Body mass estimates). At the proximal end, the crista cnemialis lateralis projects proportionally far laterally (figure 19*e*,*f*), as in *P. campestris*, *M. reinwardt*, *L. ocellata* and *La. naracoortensis*, and unlike *T. fuscirostris*, *Al. lathami* and *Ma. maleo*, in which there is less lateral extent of the crista. The facies articularis medialis is of similar depth to that of *L. ocellata*, but the whole facies articularis is wider, giving it a larger proximal articular surface overall. The crista cnemialis cranialis does not protrude far cranially, is proximodistally short, and is not deflected far laterally, as in *La. naracoortensis*, and unlike *M. reinwardt*, *L. ocellata*, *Al. lathami* and *T. fuscirostris*, in which the crista is longer, projects further cranially, and is orientated more laterally. At the distal end, the epicondylus medialis is protuberant beyond the medial rim of the condylus medialis in cranial aspect, distinguishing it from *P. campestris*, *Ma. maleo* and *Ae. arfakianus*, in which the epicondylus is less protuberant. The proximodistal length of the pons supratendineus is much greater laterally than it is medially (figure 19*e*), distinguishing it from *L. ocellata*, *Al. lathami* and *P. campestris*. The crista fibularis is shorter than in *L. ocellata* and relatively shorter than in *La. naracoortensis*, and is more distinct than in *P. campestris*, in which the crista is very weakly expressed. The impressio lig. collateralis medialis is relatively more prominent than in *L. ocellata* (as in *La. naracoortensis* and *P. campestris*), and the distal width is wider for the depth of the condylus medialis (as in *La. naracoortensis*, unlike *P. campestris*). Measurements (mm): for TL, PW, SW and DW, see table 12; width of proximal articular surface measured without crista patellaris, 16.8; proximal depth including crista cnemialis cranialis, 19.7; depth of lateral trochlea, 14.0; depth of medial trochlea, 15.3; min. shaft circumference, 15.3.

**Tarsometatarsus:** As well as those mentioned in the diagnosis, the tarsometatarsus of this species (figure 5*d*,*i*,*n*,*s*,*x*; appendix A) has the following features. It is superficially similar to tarsometatarsi of *L. ocellata*, being only slightly shorter, but it is considerably more robust, being broader overall and having a deeper shaft. The fossa infracotylaris dorsalis is much deeper and is surrounded laterally and proximally by thick ridges of bone (figure 5*d*). On the plantar surface, the fossa parahypotarsalis medialis (figure 5*s*) is proportionally wider than in *Leipoa*, as in the larger congener *La. naracoortensis*.

**Pedal phalanges:** Two phalanges are preserved (L I.1, figure 19*j*; and an ungual from an unknown digit, figure 19*k*). The I.1 is of similar size to that of *L. ocellata*. The ungual is shorter and more lateromedially compressed and deeply curved than those of any digit of *L. ocellata*, and the tuberculum extensorium is more prominent.

**Remarks:** This is one of two smaller species of megapode now known from southern Australia, the other being extant *L. ocellata*. So far, *La. olsoni* is known only from the Thylacoleo Caves, Nullarbor Plain. Its remains are rare within the Leaena's Breath Cave fossil assemblage, but bones of this species have been found in strata of both Early and Middle Pleistocene age. Remains of *L. ocellata* from the same cave deposit (see below) show that these smaller species overlapped geographically and temporally, which perhaps indicates niche partitioning. Although *La. olsoni* shares many morphological similarities with its larger congener *La. naracoortensis*, morphological differences are noted throughout the skeleton (see diagnosis and description), and these species also appear to be allopatric. Thus, there is no evidence that these taxa represent a single dimorphic species. That these species overlap temporally precludes the possibility that they are different-sized chronospecies.

#### ***Garrdimalga*** Shute, Prideaux & Worthy, gen. nov.

**Zoobank ID:** urn:lsid:zoobank.org:act:EF3C0068-4168-4A26-9504-99292BB2202E

**Type species:** *Garrdimalga mcnamarai* Shute, Prideaux & Worthy sp. nov. by monotypy.

**Etymology:** Garrdimalga, from which the name of the type locality derives, means ‘emu waterhole’ (garrdi, emu; malga, limestone/white waterhole) in the local Narungga (Nharangga) Aboriginal language of the Yorke Peninsula, South Australia [51]. Originally referring to a limestone depression where emus came to drink, the name, written as Curramulka, was later extended to the whole of the nearby township. Pronounced ‘GA-ree-mal-ga’, the ‘-rrd’ of the first syllable is a soft ‘r’, made by the tongue slightly touching the roof of the mouth and forming a slight ‘d’ sound.

**Figure 20.**
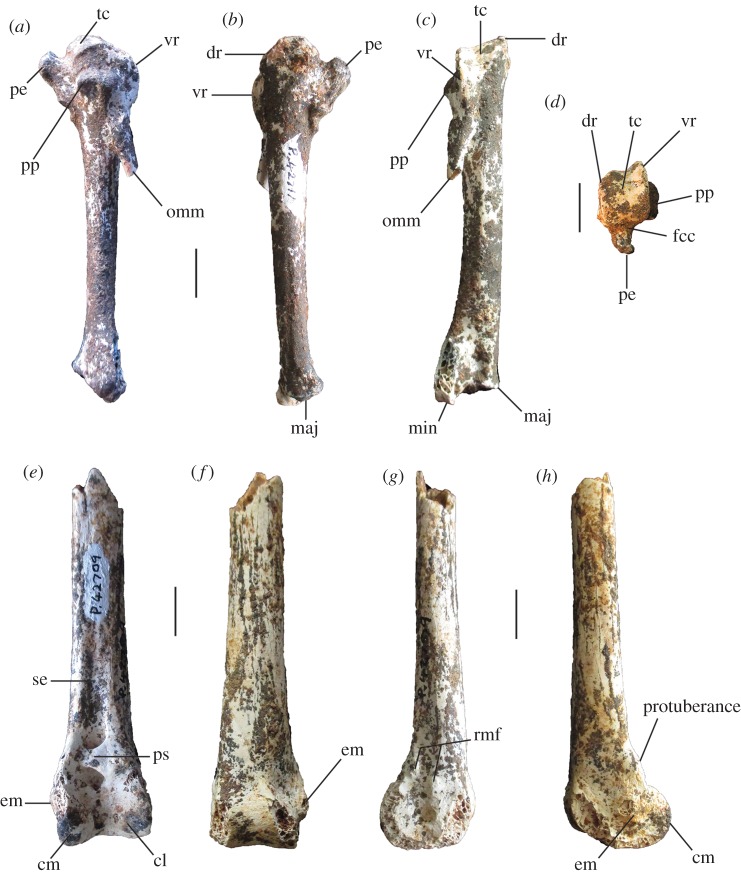
Type specimens of *Garrdimalga mcnamarai* gen. et sp. nov. Carpometacarpus, SAM P42711, holotype, in ventral (*a*), dorsal (*b*), caudal (*c*) and proximal (*d*) aspects; tibiotarsus, SAM P42709, paratype, in cranial (*e*), caudal (*f*), lateral (*g*) and medial (*h*) aspects. Scale bars, 10 mm. cl, condylus lateralis; cm, condylus medialis; dr, dorsal rim of trochlea carpalis; em, epicondylus medialis; fcc, fovea carpalis cranialis; maj, os metacarpale majus; min, facies articularis digitalis minor; omm, os metacarpale minus; pe, processus extensorius; pp, processus pisiformis; ps, pons supratendineus; rmf, retinaculi m. fibularis; tc, trochlea carpalis; se, sulcus extensorius; vr, ventral rim of trochlea carpalis.

**Diagnosis:** A genus of megapode distinguished from all extant and extinct genera by unique morphology of the carpometacarpus and tibiotarsus as follows. **Carpometacarpus** (i) The processus extensorius (figure 20*a*,*b*,*d*) is short, dorsoventrally narrow (less than half the width of the adjacent trochlea carpalis), and proximally directed. (ii) The processus pisiformis is short, blunt, does not project over the extensor process, is placed relatively close to the proximal rim of the trochlea carpalis, and its caudal portion is highly protuberant (figure 20*d*). (iii) The ventral rim of the trochlea carpalis has little caudal prominence relative to the os metacarpale minus in dorsal view (figure 20*b*), and in caudal aspect is more or less aligned with the long axis of the os metacarpale majus (figure 20*c*). (iv) The distal margin of the articular facet of the trochlea carpalis is defined by the link between the caudal end of the dorsal rim and the distal end of the ventral rim, which traverses the caudal facies at a wide angle relative to the axis. **Tibiotarsus** (i) Just proximal of the epicondylus medialis and the condylus medialis, there is a cranial protuberance of the shaft (figure 20*h*). (ii) The pons supratendineus is placed proportionally further proximally than in all other taxa examined (i.e. there is no overlap of the pons and the condylus lateralis, and the pons is very constricted at its medial side, resulting in a very large distal opening of the canalis extensorius (figure 20*e*).

**Differential diagnosis:** Species in other genera differ from *Garrdimalga* as follows. **Carpometacarpus:** (i) In all other genera apart from *Progura*, the processus extensorius is orientated more cranially. *Progura* is distinguished by having a processus that is relatively wider dorsoventrally (more than half the width of the adjacent trochlea carpalis). (ii) The processus pisiformis is relatively longer and more cranially protuberant in *Leipoa*, *Alectura* and *Megapodius*. In all extant and extinct genera examined, the caudal portion of the processus pisiformis is less protuberant relative to the cranial portion than in *Garrdimalga*, as seen in proximal aspect. (iii) In all genera examined besides *Alectura*, the ventral rim of the trochlea carpalis projects further caudally relative to the os metacarpale minus, and in all genera, the ventral rim of the trochlea is orientated about 30° to the long axis of the os metacarpale majus in caudal aspect, rather than aligned with the long axis as in *Garrdimalga*. (iv) In all other genera examined, the distal margin of the articular facet of the trochlea carpalis traverses the caudal facies at a shallower angle. **Tibiotarsus:** In all other genera, (i) the shaft proximal of the epicondylus medialis and the condylus medialis is concave, and (ii) the pons supratendineus overlaps proximodistally with the condylus lateralis. In all genera apart from *Progura,* the pons is placed relatively further distally on the shaft (proximal margin of the pons is a little further proximal in *Progura*). The only other genus in which the pons is constricted medially is *Latagallina*, but in this genus, it is placed slightly further distally on the shaft, thus the distal opening of the canalis extensorius is relatively smaller. For visual comparison, the diagnostic material is depicted alongside the bones of other extinct species in the electronic supplementary material, figure S1.

#### ***Garrdimalga mcnamarai*** Shute, Prideaux and Worthy, sp. nov.

##### (figures 20 and 21)

**Zoobank ID:** urn:lsid:zoobank.org:act:336EEFFA-C49F-4186-9C8A-BF97A02CE94B

**Figure 21.**
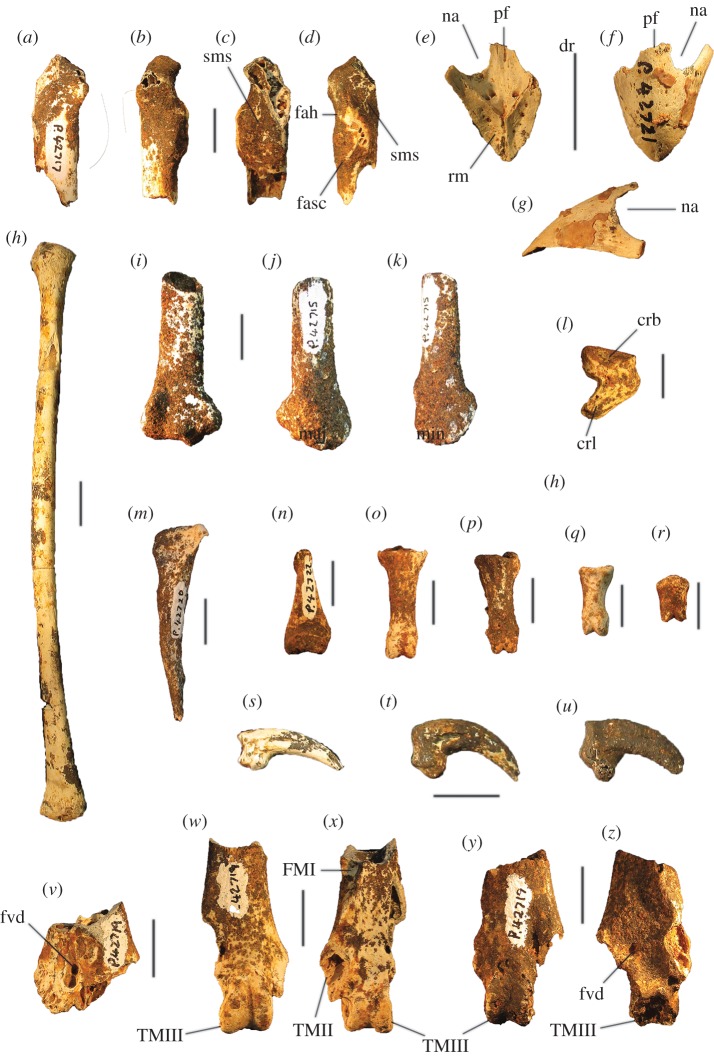
Referred material of *G. mcnamarai* sp. nov. Coracoid, SAM P42717, left omal fragment, in ventral (*a*), dorsal (*b*), medial (*c*) and lateral (*d*) aspects; premaxilla, SAM P42721, in ventral (*e*), dorsal (*f*) and lateral (*g*) aspects; radius, SAM P42712, (*h*); ulna, SAM P42715, in dorsal (*i*), caudal (*j*) and ventral (*k*) aspects; os carpi ulnaris, SAM P42723 (*l*); fibula, SAM P42720 (*m*); metatarsal, SAM P42722 (*n*); pedal phalanges (*o*–*r*) and unguals (*s*–*u*) (SAM P42724); tarsometatarsi, SAM P42719, right distal fragments (*v*–*x*), and left distal fragment (*y*,*z*). Scale bars, 10 mm. crb, crus breve; crl, crus longum; fah, facies articularis humeralis; fasc, facies articularis scapularis; FMI, fossa metatarsi I; fvd, foramen vascularis distalis; maj, os metacarpale majus; min, facies articularis digitalis minor; na, naris; pf, processus frontalis; rm, rostrum maxillare; sms, sulcus m. supracoracoidei; TMII, trochlea metatarsi II; TMIII, trochlea metatarsi III.

**Holotype:** SAM P42711 (R carpometacarpus, missing os metacarpale minus).

**Paratypes:** SAM P42709 (dL tibiotarsus); SAM P42710 (dR tibiotarsus).

**Referred material:** SAM P42712 (R radius); SAM P42713 (dR radius); SAM P42714 (pR radius); SAM P42715 (2 dL ulna); SAM P42716 (3 dR ulna); SAM P42717 (2 L, 1R coracoid, omal fragments); SAM P42718 (R MII.1); SAM P42719 (1dL, 4dR tarsometatarsi); SAM P42720 (R, L fibulae); SAM P42721 (premaxilla); SAM P42722 (3 metatarsi); SAM P42723 (2R os carpi ulnare); SAM P42724 (pedal phalanges, 2 L, 2R IV.1; 3 L, 4R II.1; 1 I.1; 1R III.1; 4 other phalanges, 17 unguals undetermined to digit).

**Type locality:** Curramulka Quarry (site RF 95), Curramulka (34°42'11.8^″^ S, 137°42'14.3^″^ E), Yorke Peninsula, South Australia (figure 2).

**Stratigraphy, age and fauna:** All materials for this taxon was excavated from Curramulka Quarry (site RF 95) by J. A. McNamara between 1997 and 1999, and are considered Pleistocene in age (see Key locations).

**Diagnosis:** As for the genus.

**Etymology:** The species name, *mcnamarai*, is in honour of Jim McNamara, formerly of the South Australian Museum, who collected the holotype and referred material of this species from Curramulka Quarry between 1997 and 1999.

**Description and comparisons:** The surface of all bones of this species is mottled with a fine dark grey mineral coating (figures 20 and 21). With the exception of the holotype carpometacarpus and the pedal phalanges, most remains are fragmentary, with some apparently having been broken during collection. Key measurements are given in table 13, with additional measurements given in the text where necessary. This is a species larger than any extant species of megapode and extinct *La. olsoni*, and approaching the size of *P. campestris*. It has a carpometacarpus that is of similar length to that of *P. campestris*, but is proportionally larger compared with the leg elements.

**Table 13.** Long-bone measurements (mm) of *G. mcnamarai* holotype and referred material; TL, total length; PW, proximal width; SW, midshaft width; DW, distal width.

element/side	catalogue no.	TL	PW	SW	DW
holotype					
carpometacarpus, R	SAM P42711	74.7	20.3	—	13.9
referred material					
radius, R	SAM P42712	130.6	9.2	5.3	10.9
ulna, dL	SAM P42715	—	—	—	17.7
ulna, dR	SAM P42716	—	—	—	17.3
tibiotarsus, dL	SAM P42709	—	—	10.0	18.0
tibiotarsus, dR	SAM P42710	—	—	—	17.3

**Premaxilla:** The anterior portion of a premaxilla (figures 7*g*–*i* and 21*e*–*g*) shows that the bill of this species was wider and deeper than in *L. ocellata* or *M. reinwardt*, and had a tip that was proportionally short, as in *La. naracoortensis*, rather than elongate as in *P. campestris*. The symphyseal zone is relatively short compared with *L. ocellata*, *P. campestris* and *La. naracoortensis* (figures 7*h* and 21*e*). For a shape comparison with selected taxa, see figure 7. Measurements (mm): depth, measured at the level of the anterior edge of the nares, 7.6; width at the level of anterior edge of nares, 9.7; length of ventral symphyseal zone, 5.7.

**Carpometacarpus:** In addition to the diagnostic features noted for the genus (see above), the carpometacarpus of this species (figure 20*a*–*d*; electronic supplementary material, figure S1) has the following characteristics. The proximal end is narrower relative to length than in the other large extinct species *P. campestris* and *La. naracoortensis*, and is proportionally more similar to carpometacarpi of *L. ocellata* and *Al. lathami* despite being much larger than both. As little of the os metacarpale minus is preserved, the extent of divergence from the os metacarpale major cannot be assessed relative to other species, but its proximal end differs from species of *Latagallina* and *P. campestris* by being rotated ventrally, such that its broadest surface is orientated caudo-ventrally, rather than caudally. The scar for the flexor attachment is a single tuberculum, as in species of *Latagallina*, *L. ocellata* and *Al. lathami*, and distinct from both species of *Progura* where the tuberculum has two distinct parts with the distal one placed partially or entirely within the spatium intermetacarpale. In *G. mcnamarai*, this tuberculum is located immediately proximal to the spatium intermetacarpale, as in species of *Latagallina*, rather than overlapping the spatium intermetacarpale and the synostosis of the metacarpals as in *L. ocellata* and *Al. lathami*. There is a discrete fovea carpalis caudalis as in *L. ocellata* and species of *Progura* (absent in species of *Latagallina*).

**Os carpi ulnare:** These are of similar size to those of *P. campestris*, but in the mature specimen of *G. mcnamarai* (figure 21*l*), the crus breve and crus longum are a little broader than in that species.

**Radius:** The complete specimen (SAM P42712; figure 21*h*), which is from a slightly immature individual, is somewhat shorter and narrower than radii of *P. campestris* and *La. naracoortensis*, but is wider and presumably longer than that of *La. olsoni*. Measurements: for TL, PW, SW and DW, see table 13.

**Ulna:** Five fragments of distal ulnae are preserved, registered under two catalogue numbers. Total length is unknown, but based on length of a complete radius (above), a complete ulna would be approximately 140 mm, thus longer than in any extant species of megapode and the extinct *La. olsoni*, but slightly shorter than that of *P. campestris* (148.2 mm), and below the size range of *La. naracoortensis* (144.8–173.9 mm). The best-preserved distal fragment (SAM P42715; figure 21*i*–*k*) is unusual in having a robust ridge extending dorsally from the tuberculum carpale, which defines a marked groove immediately proximal to it. This feature has not been seen in other megapodes. The distal fragments are a little wider than the distal ulna of *La. olsoni*, approach the size of *P. campestris*, and are much smaller than those of *La. naracoortensis*.

**Coracoid:** The omal fragments of the coracoid (e.g. figure 21*a*–*d*) are clearly larger than those of extant megapodes, but are smaller than those of *P. campestris* and *La. naracoortensis*, and seem small relative to other skeletal elements of this species. Given their fragmentary nature and state of preservation, it is not possible to tell whether the coracoid fragments belong to adults or juveniles, as observations of nearly 60 fossil coracoid specimens belonging to *La. naracoortensis* show that the omal end of the coracoid reaches osteological maturity (i.e. distinct morphology, absence of surface porosity) more quickly than does the sternal end. The facies articularis humeralis is slightly concave (figure 21*d*), a little less so than in *P. campestris* but more so than in *La. naracoortensis.* Maximum dorsoventral depth measured at the facies articularis scapularis is 10.5 mm (cf. 12.2 mm in *P. campestris* holotype, and 13.1 mm in a specimen of *La. naracoortensis* (SAM P51369).

**Tibiotarsus:** The two distal tibiotarsi (left pictured in figure 20*e*–*h*; electronic supplementary material, figure S1), left and right but apparently not a pair, are somewhat eroded but preserve the distal anatomy quite well. They are much larger than the tibiotarsi of extant megapodes and of *La. olsoni*, but smaller than those of *La. naracoortensis*. In addition to the diagnostic features noted for the genus (see above), the tibiotarsus has features as follows. The distal shaft has similar dimensions to that of *P. campestris*, but terminates in smaller condyles, which indicates that the corresponding proximal articular surface of the tarsometatarsus of this species would also be narrower and shallower than in *P. campestris*. Lateral condylar depth (16.3 mm) approaches parity with distal width (17.5 mm). The epicondylus medialis is exceptionally large and protuberant (figure 20*e*,*f*,*h*), and is visible beyond the rim of the medial rim of the condylus medialis in cranial aspect, distinguishing it from *P. campestris*, *Ma. maleo* and *Ae. arfakianus*. The retinaculi m. fibularis form two parallel, longitudinal ridges that are strongly elevated on the lateral shaft facies immediately caudal to the condylus lateralis, and enclose a very deep sulcus, which opens proximally into a concave shaft surface. The retinaculi are less prominent in *L. ocellata*, *T. fuscirostris* and *P. campestris*, and while they are prominent in *La. naracoortensis*, *La. olsoni*, *Al. lathami* and *M. reinwardt*, in those species the sulcus is somewhat shallower, and the shaft proximal of the sulcus is flat to convex. The lateral facies proximal to the retinaculi forms a plane that is angled more mediocranially/caudolaterally than in other taxa, exposing more of this facies in cranial aspect (figure 20*e*). The sulcus extensorius is proportionally longer and deeper than in all species examined apart from *Al. lathami*. Measurements (mm): for SW and DW, see table 13; depth of lateral condyle, (SAM P42709) 16.1, (SAM P42710) 17.1; depth of medial condyle (SAM P42709) 18.4.

**Tarsometatarsus:** The available tarsometatarsi of this species are poorly preserved (figure 21*v*–*z*). One distal fragment (not pictured) is apparently a juvenile, and possibly also shows pathological modification to the shaft. The other bones are sufficiently well preserved to show that the plantar opening of the foramen vasculare distale (figure 21*z*) is small, and on the dorsal surface, the proximal end of the canalis interosseus distalis is visible in the same fossa as the foramen vasculare distale (figure 21*v*). Trochlea metatarsi III is of similar width and depth to those of *P. campestris*, but the distal shaft is more slender in *G. mcnamarai*, with trochleae II and IV not flaring so widely, thus the distal width of the tarsometatarsus would probably have been narrower if complete. This, combined with the smaller inferred size of the proximal end of the tarsometatarsus of *G. mcnamarai* compared with *P. campestris* (see description of tibiotarsus for *G. mcnamarai*), implies that *G. mcnamarai* had overall a smaller tarsometatarsus than *P. campestris.* However, shaft length is unknown, and so it is impossible to know whether the tarsometatarsus was proportionally robust or gracile compared with other taxa.

**Pedal phalanges:** Phalanges of this species are robust and are of similar size to those of *P. campestris* and *La. naracoortensis*, but because *G. mcnamarai* has somewhat smaller tarsometatarsi and tibiotarsi than those species, its feet were presumably proportionally larger. Phalanx III.1 is of similar length but a little narrower than that of *La. naracoortensis*. Like that species, it has a nearly symmetrical proximal articular facet (asymmetrical in *P. campestris*), and while the proximoplantar surface is not as deeply excavated as in *La. naracoortensis*, it is more excavated than in *P. campestris*. Phalanx II.1 is slightly shorter than in *P. campestris*, with a proximal articular facet of similar overall size, although it is deeper than wide, not wider than deep as in *P. campestris*. Phalanx IV.1 is a little shorter than that of *P. campestris*. The unguals are short, deep, lateromedially compressed, deeply curved and have very large extensor tubercles (figure 21*s*–*u*).

**Remarks:** So far, the remains of this species are insufficient to include it in a meaningful phylogenetic analysis, and its relationship to other genera of megapodes is unknown. Several features, including the single scar for the flexor attachment entirely proximal to the spatium intermetacarpale on the carpometacarpus, the broad trochlea carpalis and the short, wide mandible, may suggest an affinity with species of *Latagallina*. However, the carpometacarpus shares other features in common with other taxa, including minimal caudal projection of the ventral rim of the trochlea carpalis as in *Al. lathami*, and a distinct fovea carpalis caudalis as in species of *Progura*.

#### ***Leipoa ocellata*** Gould, 1840

**Referred material: *Leaena's Breath Cave, Nullarbor Plain***—WAM 15.9.2, R humerus; ***Main Fossil Chamber,*** ***Victoria Fossil Cave, Naracoorte***—SAM P25852, R tarsometatarsus, missing most of the hypotarsus and cotylae; SAM P41531, L humerus; SAM P41532, R tibiotarsus; SAM P42077, dR femur; SAM P42708, dL femur. ***Henschke's Fossil Cave, Naracoorte***—SAM P41704, pL femur; SAM P41705, pL tarsometatarsus; SAM P41706, pL/dR tibiotarsus; SAM P417047, R tibiotarsus.

The above specimens, all of which belong to mature individuals, are referred to *L. ocellata* because they do not differ appreciably in size or morphology from modern specimens of that species.

**Stratigraphy, age and fauna:** The humerus (WAM 15.9.2) was excavated from Leaena's Breath Cave, Nullarbor Plain, at a depth of 105–110 cm beneath the sediment floor, in stratigraphic Unit 3, Quadrat 2, Pit B (2.58–0.78 Ma [28]; Early Pleistocene). The specimens from Victoria Fossil Cave, Naracoorte, all derive from stratigraphic layers that are of Middle Pleistocene age (greater than 212 ka [52]). Specimens from Henschke's Fossil Cave are of Pleistocene age, probably Middle or Late Pleistocene [37] (table 14). The specimens from the Naracoorte Caves occur within the same deposit as fossils of *La. naracoortensis*. Measurements: for TL, PW, SW and DW, see table 14. Measurements of modern specimens of *L. ocellata* are given in table 15 for comparison.

**Table 14.** Measurements (mm) of Pleistocene *L. ocellata* fossils from Leaena's Breath Cave, Nullarbor Plain (LBC) and Victoria Fossil Cave (VFC) and Henschke's Fossil Cave (HFC), Naracoorte. TL, total length; PW, proximal width; SW, shaft width; DW, distal width.

element/side	catalogue no.	provenance	age (years)	TL	PW	SW	DW
humerus, R	WAM 15.9.2	LBC	>780 000	97.9	22.1	9.1	17.9
humerus, L	SAM P41531	VFC	>212 000	106.1	24.8	9.4	21.1
femur, pL	SAM P41704	HFC	(?)<126 000	—	—	—	—
femur, dR	SAM P42077	VFC	>212 000	—	—	—	19.4
femur, dL	SAM P42708	Fossil Chamber, VFC	>212 000	—	—	—	20.6
tibiotarsus, R	SAM P41532	VFC	>212 000	129.6	17.5	8.7	14.3
tibiotarsus, pL/dR	SAM P41706	HFC	(?)<126 000	—	—	8.2	14.7
tibiotarsus, R	SAM P41707	HFC	(?)<126 000	120.2	16.2	8.2	14.7
tarsometatarsus, R	SAM P25852	VFC	>212 000	75.8	—	8.1	17.4
tarsometatarsus, pL	SAM P41705	HFC	(?)<126 000	16.7	—	—	—

**Table 15.** Summary data (mm) for modern skeletons of *L. ocellata*; TL, total length; PW, proximal width; SW, shaft width; DW, distal width; data from Worthy *et al*. ([21]; electronic supplementary material).

element		TL	PW	SW	DW
humerus (*n* = 2)	range	100.2–100.5	22.3–23.1	8.5–8.8	19.2–19.5
femur (*n* = 12)	mean (s.d.)	85.2 (2.8)	20.2 (0.9)	8.6 (0.6)	18.2 (0.3)
	range	81.3–90.5	18.9–21.5	7.9–10.0	18.2–19.3
tibiotarsus (*n* = 13)	mean (s.d.)	122.0 (5.9)	16.4 (0.3)	7.7 (0.5)	13.8 (0.4)
	range	112.8–134.1	15.6–16.8	6.5–8.6	13.1–14.6
tarsometatarsus (*n* = 13)	mean (s.d.)	73.5 (3.1)	15.3 (0.5)	8.9 (0.4)	16.7 (0.3)
	range	67.8–79.2	14.3–15.9	8.1–9.8	15.9–17.1

**Remarks:** It has previously been stated that modern-sized *L. ocellata* fossils do not occur in the same deposits as large extinct megapodes [1], and it has been proposed that the modern malleefowl is a phyletic dwarf that evolved from the much larger *P. gallinacea* during the Late Pleistocene [24]. However, WAM 15.9.2, the humerus of *L. ocellata* from the Nullarbor Plain, demonstrates that modern-sized malleefowl were already present in Australia during the Early Pleistocene, in sediments that pre-date the Late Pleistocene by at least half a million years. Furthermore, the humerus comes from the same stratigraphic unit in Leaena's Breath Cave as the holotype of *P. campestris* and a referred carpometacarpus of *La. olsoni* (see species accounts), thus the species was roughly coeval with two other taxa, one somewhat larger, and one very much so. At Naracoorte, modern-sized *L. ocellata* fossils (table 14) occur in Middle and probable Late Pleistocene sediments, along with remains of the very much larger extinct species *Latagallina* (formerly *Progura*) *naracoortensis.* The phylogenetic relationship between *L. ocellata* and the extinct Plio-Pleistocene taxa is uncertain (see Phylogenetic analysis), but there is no evidence to support the proposal that *L. ocellata* is a dwarf of any of the extinct taxa we have described herein.

### 3.3. Body mass estimates

In all species where both the femoral and tibiotarsal equations [42] could be used, except for *M. reinwardt*, estimates derived from the femur were greater than those from the tibiotarsus (table 16). The magnitude of difference between femoral and tibiotarsal estimates varied greatly among species, being equal in *M. reinwardt* but differing by nearly one-third in *La. olsoni*, for example. Femoral equations also produced high estimates for three extant species (2.3 kg for *T. fuscirostris* versus 1.4–1.6 kg recorded for modern specimens; mean 2.9 kg for *Al. lathami* versus 2.2–2.5 kg for modern; and 1 kg for *M. eremita* versus 0.64–0.71 kg for modern [53]). This is consistent with our findings in other taxa [52,54], where we identified an apparent tendency for femoral equations to overestimate body mass.

**Table 16.** Body mass calculations for extinct and extant megapodes. † = extinct species; estimates based on femur circumference use the ‘heavy-bodied birds’ equation of Campbell & Marcus [42] as follows: log_10_*M* = 2.293 × log_10_LCf + 0.110, where *M* is the mass (g), and LCf the least shaft circumference of the femur (mm); estimates based on minimum circumference of the tibiotarsus use the ‘heavy-bodied birds’ equation of Campbell & Marcus [42] as follows: log_10_*M* = 2.416 × log_10_LCt + 0.140, where *M* is the mass (g), and LCt the least shaft circumference of the tibiotarsus (mm); estimates based on the minimum width of the tarsometatarsus use an equation for the Galliformes derived from figure 2; electronic supplementary material, tables S2 and S4 of Field *et al*. [43] as follows: log_10_(BM) = 2.069 (log_10_TW) + 3.709, where BM is the body mass (g), and TW the minimum shaft width of the tarsometatarsus (mm); all calculations expressed in kg.

species (no. of specimens)		estimate from femur (kg)	estimate from tibiotarsus (kg)	estimate from tarsometatarsus (kg)
*Progura gallinacea*† (*n* = 1)		—	—	7.7
*Progura campestris*†		7.5	6.2	4.8–6.0
(femur and tibiotarsus, *n* = 1; tarsometatarsi, *n* = 2)				
*Latagallina naracoortensis*†	mean (s.d.)	5.8 (0.91)	5.2 (0.91)	6.1 (0.8)
(femora, *n* = 7; tibiotarsi, *n* = 13; tarsometatarsi, *n* = 23)	minimum	5.1	3.7	5.0
	maximum	7.4	6.5	7.7
*Latagallina olsoni*† (*n* = 1)		3.8	2.9	3.6
*Garrdimalga mcnamarai*† (*n* = 1)		—	5.2	—
*Talegalla fuscirostris* (*n* = 1)		2.3	1.9	1.9
*Leipoa ocellata*	mean (s.d.)	—	—	2.9 (0.3)
(femora and tibiotarsi, *n* = 2; tarsometatarsi, *n* = 13)	minimum	2.0	1.8	2.2
	maximum	2.3	2.2	3.5
*Alectura lathami*	mean (s.d.)	2.9 (0.33)	2.4 (0.18)	3.1 (0.7)
(femora and tibiotarsi, *n* = 5; tarsometatarsi, *n* = 12)	minimum	2.4	2.1	2.2
	maximum	3.3	2.7	4.6
*Aepypodius arfakianus* (*n* = 1)		—	2.3	1.8
*Megapodius eremita* (*n* = 1)		1.0	0.9	1.5
*Megapodius reinwardt* (*n* = 1)		1.9	1.9	2.1

Estimates from the tibiotarsus for extinct species are probably more realistic because for most extant species, they more closely approximate known body masses recorded in Dunning [53]. Thus, the holotype of *P. campestris* is estimated to have weighed 6.2 kg, mature individuals of *La. naracoortensis* an average of 5.2 kg, *G. mcnamarai* 5.2 kg (based on paratype tibiotarsus) and the holotype of *La. olsoni* 2.9 kg (table 16). Estimated mass for *G. mcnamarai* is approximate, because the smallest measurable circumference was immediately distal of the broken midshaft, and it is not known if the shaft tapered proximal of this point. Given that neither a tibiotarsus nor a femur of *P. gallinacea* is known, the only estimate for this species was based on the minimum shaft width of the most complete tarsometatarsus [43] (QM F1143), which produced an estimated mass of 7.7 kg for this species. These calculations show that all four extinct species for which we could estimate mass were heavier than extant megapodes, but even the largest species *P. gallinacea* was approximately 3.5–4.5 times lighter than the extinct flightless stem-galliform *S. neocaledoniae* [21].

### 3.4. Simpson log-ratio diagram

**Figure 22.**
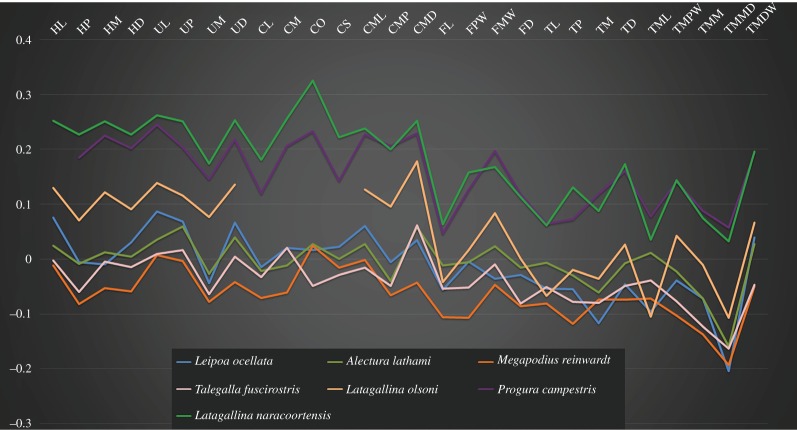
Log-ratio diagram showing proportions of the post-cranial skeleton in extinct and extant megapodes; all measurements in millimetres, log-transformed and expressed as a proportion of comparator galliform species, *Ga. gallus* (domestic chicken). HL, humerus length; HP, humerus proximal width; HM, humerus midshaft width; HD, humerus distal width; UL, ulna length; UP, ulna proximal width; UM, ulna midshaft width; UD, ulna distal width; CL, coracoid length; CM, coracoid midshaft width; CO, coracoid omal width; CS, coracoid sternal width; CML, carpometacarpus length; CMP, carpometacarpus proximal width; CMD, carpometacarpus distal width; FL, femur length; FPW, femur proximal width; FMW, femur midshaft width; FD, femur distal width; TL, tibiotarsus length; TP, tibiotarsus proximal width; TM, tibiotarsus midshaft width; TD, tibiotarsus distal width; TML, tarsometatarsus length; TMPW, tarsometatarsus proximal width; TMM, tarsometatarsus midshaft width; TMMD, tarsometatarsus midshaft depth; TMDW, tarsometatarsus distal width.

The Simpson log-ratio diagram (figure 22) compares the proportions of the post-cranial skeleton for the three most complete extinct species (*La. naracoortensis*, *La. olsoni* and *P. campestris*) and the four extant species of megapode for which we had access to complete reference skeletons (*L. ocellata*, *M. reinwardt*, *Al. lathami* and *T. fuscirostris*). Measurements of *P. campestris* and *La. olsoni* are from the associated holotype skeletons (tables 2 and 12), whereas measurements for *La. naracoortensis* are based on mean measurements of skeletal remains from the Naracoorte Caves (tables 4–11). All measurements are standardized as a proportion of the reference species, the domestic chicken *Ga. gallus*, which is represented by a straight zero line on the horizontal axis.

Compared with *Ga. gallus*, the megapodes have: ulnae that are longer relative to their width; coracoids that have stouter shafts relative to length, and with the exceptions of *L. ocellata* and *T. fuscirostris*, a proportionally much wider omal end; carpometacarpi that have narrow proximal ends compared with distal ends; femora that are notably short relative to the size of the pectoral elements, and with a stout shaft in all species except *L. ocellata*; and tarsometatarsi that have exceptionally wide distal ends.

The three included extinct species (*La. naracoortensis, La. olsoni* and *P. campestris*) share body proportions that are more similar to one another than to extant species, with similarities between the two species of *Latagallina* (indicated by strong parallelism of their lines) being particularly striking. *Leipoa ocellata* has proportions seemingly dissimilar to all comparator species (e.g. long but gracile humerus, narrow femoral shaft, relatively robust coracoid). The diagram reveals proportional differences within the hindlimb between the two largest included species, *La. naracoortensis* and *P. campestris*, with *P. campestris* having a femur with a wider midshaft relative to its other dimensions, a narrower proximal tibiotarsus and a longer tarsometatarsus relative to width. A further insight is the difference in relative proportions of the pectoral girdle versus the hindlimb in these two large species. The size of their leg elements overlaps (figure 22), but the pectoral girdle of *P. campestris* is somewhat smaller throughout, with the humerus, ulna and coracoid being shorter and narrower than in *La. naracoortensis*.

### 3.5. Phylogenetic analysis

**Figure 23.**
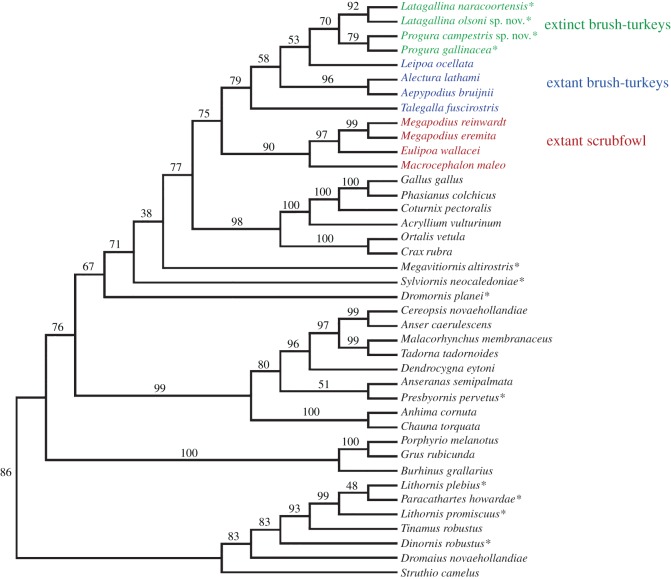
Bootstrap consensus tree with molecular backbone constraint for all extant taxa. Relationships between extant species of Megapodiidae were constrained based on a recent molecular phylogeny of that family [4], and all other extant non-megapode taxa as described by Worthy *et al*. [21]. Relationships of the fossil taxa were unconstrained, and they were therefore free to move to their optimal positions around this framework. *Extinct taxon. Tree statistics: length, 1453; consistency index, 0.27; homoplasy index, 0.73; retention index, 0.63). Numbers are bootstrap probability values expressed as a percentage.

With the four most skeletally complete fossil megapode taxa added to the matrix (*P. gallinacea*, *P. campestris*, *La. naracoortensis* and *La. olsoni*), a parsimony analysis produced a consensus of one tree (tree length, 1453; consistency index, 0.27; retention index, 0.63; figure 23). As expected, the extinct taxa were all found to be included in the clade comprising the Megapodiidae.

With the inclusion of extinct taxa, the clade Megapodiidae and the constituent ‘scrubfowl’ and brush-turkey clades [4] retained high bootstrap support (75%, 90% and 75%, respectively; figure 23). All four of the extinct species were in a derived position within the brush-turkey clade, and strongly excluded from the scrubfowl clade. The two species of *Progura* and the two species of *Latagallina* formed their own clade with 70% bootstrap support, with a weakly supported sister relationship between this clade and *L. ocellata* (53%). Furthermore, there was high support for the fossil megapode genera that we identified in our morphological examinations (see Systematic palaeontology): *P. gallinacea* and *P. campestris* formed a clade with 79% bootstrap support and *La. naracoortensis* and *La. olsoni* formed a clade with 92% support. Notably, neither species of the long-legged genus *Progura* was attracted to extant species with similarly elongate tarsometatarsi (*Macrocephalon*, *Aepypodius*, *Talegalla*, *Alectura*).

Our analysis found the following character state transformations for key clades and species as follows. The *Progura*/*Latagallina* clade is united by 14 synapomorphies, of which the following four character states of the post-cranial skeleton are unambiguous (synapomorphic state in parentheses): Character 138, CI = 0.25, 0 ⇒ 1 (distal humerus, tuber. supracondylare dorsale, in cranial view, not prominent from epicondylus dorsalis); Character 197, CI = 0.111, 1 ⇒ 0 (proximal femur, cranial facies, trochanter, elongate, extends distally past the level of the caput femoralis a distance exceeding the proximodistal width of the caput femoralis); Character 250, CI = 0.25, 0 ⇒ 1 (tarsometatarsus, cotyla medialis dorsoplantarly elongate, protruding dorsal to cotyla lateralis); Character 263, CI = 0.25, 0 ⇒ 1 (tarsometatarsus, tuberositas m. tibialis cranialis has two distinct tuberosities).

The extinct genus *Progura* is defined by seven synapomorphies of the post-cranial skeleton, of which the following three are unambiguous: Character 97, CI = 0.167, 0 ⇒ 1 (coracoid, omal end, ventral facies, processus acrocoracoideus, depth of sulcus medial to facies artic. humeralis and sternal to impressio ligamentum acrocoracohumeralis is deep); Character 265, CI = 0.222, 1 ⇒ 0 (tarsometatarsus, foramina vascularis proximalis, of roughly equal size); Character 275, CI = 0.333, 0 ⇒ 1 (tarsometatarsus, anterior end of canalis interosseus distalis largely or completely exposed dorsally by reduction in bony covering). The larger species, *P. gallinacea*, is defined by three autapomorphies, two of which are unambiguous: Character 171, CI = 0.143, 0 ⇒ 1 (distal carpometacarpus, length synostosis metacarpals II and III, from distal end of spatium intermetacarpale to facies articularis digitalis minoris/facet for digit III, is greater than or equal to the width of synostosis); Character 257, CI = 0.133, 1 ⇒ 0 (proximal tarsometatarsus, width hypotarsus adjacent to cotylae is distinctly less than half proximal width). The smaller species in this genus, *P. campestris*, is defined by two autapomorphies, one of which is unambiguous: Character 257, CI = 0.133, 1 ⇒ 2 (proximal tarsometatarsus, width hypotarsus adjacent to cotylae, distinctly more than half proximal width).

The extinct genus *Latagallina* is defined by six unambiguous synapomorphies of the post-cranial skeleton: Character 193, CI = 0.25, 0 ⇒ 1 (femur, caudal facies, pneumatic openings adjacent to facies articularis antitrochanterica, present and large); Character 205, CI = 0.143, 0 ⇒ 1 (distal femur, cranial aspect, orientation of condylus lateralis markedly divergent); Character 212, CI = 0.286, 0 ⇒ 1 (femur, condylus medialis, profile in medial aspect is subangular between articular surface of condyle and its cranial surface); Character 213, CI = 0.091, 0 ⇒ 1 (distal femur, width of sulcus patellaris in cranial view, taken at half the depth of the bounding condyles, narrow and deep, less than width of condylus lateralis plus trochlea fibularis); Character 258, CI = 0.333, 0 ⇒ 1 (tarsometatarsus, hypotarsus, major hypotarsal ridge, distal end, ridge terminates abruptly, drops steeply to shaft); Character 262, CI = 0.286, 0 ⇒ 1 (tarsometatarsus, corpus tarsometatarsi, sulcus extensorius, shallow and broad proximally, flattens out distally). The larger species, *La. naracoortensis*, is distinguished by five autapomorphies, of which the following four transformations are unambiguous: Character 118, CI = 0.333, 1 ⇒ 2 (proximal humerus, fossa pneumotricipitalis dorsalis, between incisura capitis and tuberculum dorsale, wide, shallow fossa ≥ width ventral pneumotricipital fossa); Character 144, CI = 144, 0 ⇒ 1 (humerus, width of space between the facet on the tuber. supracondylare ventrale and the proximoventral apex of the dorsal condyle is wide, gap wider than facet); Character 257, CI = 0.133, 1 ⇒ 2 (proximal tarsometatarsus, hypotarsus, width adjacent to cotylae, distinctly more than half proximal width); Character 264, CI = 0.20, 0 ⇒ 1 (tarsometatarsus, tuberositas m. tibialis cranialis dorsally prominent). Three unambiguous autapomorphies define the smaller species in this genus, *La. olsoni*: Character 231, CI = 0.25, 1 ⇒ 0 (tibiotarsus, distal end, epicondylus medialis with internal ligamental prominence pronounced, visible in anterior view); Character 237, CI = 0.125, 1 ⇒ 0 (tibiotarsus, distal end, junction of crista trochlea cartilaginis tibialis and rim of condylus medialis, not marked by distinct shallow notch at mid-depth); Character 279, CI = 0.214, 1 ⇒ 0 (tarsometatarsus, foramen vasculare distale large).

The relationships between the six brush-turkey genera (*Talegalla*, *Leipoa*, *Alectura*, *Aepypodius*, *Progura* and *Latagallina*) show some conflict, with *Leipoa* being weakly supported (53%) as the sister taxon to the fossil genera. The more inclusive clade of (*Leipoa* + *Progura* + *Latagallina*) and the well-supported (96%) pairing of *Aepypodius*/*Alectura* also had weak support (58%). This suggests that there is conflict in the data about whether *Leipoa* or the *Alectura*/*Aepypodius* clade is the sister group of the fossil clade. Discovery of more complete fossils of the new megapodes would probably resolve some of the ambiguity currently created by missing data. Nevertheless, the analysis supports the generic distinction of *Progura* and *Latagallina* and a sister group relationship between these genera. The relationships between the *Progura*/*Latagallina* clade and *Leipoa*, and between *Progura*/*Latagallina* and the *Alectura*/*Aepypodius* clade, should be considered unresolved on present evidence.

## 4. Discussion

### 4.1. Late Cenozoic diversity

The last review of fossil megapodes concluded that there was evidence for just one Late Cenozoic species in Australia, *Progura* (or *Leipoa*) *gallinacea* [24]. We confirm that *P. gallinacea* is the only representative of the family recognized to date from Pliocene assemblages, but from Pleistocene deposits, we record fossils of three extinct genera, comprising five species, plus extant *L. ocellata*. The subspecific diversity of *Al. lathami* and *M. reinwardt* [5] also implies their presence in the Pleistocene. Thus, as a minimum, Australia was probably home to six genera and eight species of megapode during the Late Cenozoic. This fundamentally alters our understanding of the recent history of the Megapodiidae, and shows that the estimated loss of half of the megapode species, mainly species of *Megapodius*, from Pacific islands during the Quaternary [6] can also be extended to Australia.

### 4.2. Evolutionary relationships of extinct and extant taxa

While the phylogenetic character set that we used [21] was designed to determine the relationships of galloanserine taxa in general and did not have a focus on crown group megapodes, our analysis nonetheless identified close relationships among the extinct species, and clarified evolutionary relationships between them and extant megapodes. The analysis identified two strongly supported species pairs among the extinct taxa—one of *Progura* and one of *Latagallina—*which accords with our generic distinctions (see Systematic palaeontology). The analysis also placed the extinct *Progura +Latagallina* clade in a highly derived position within the ‘brush-turkeys’ [4], revealing previously obscure generic and species diversity within this endemic Australo-Papuan branch of the Megapodiidae. So far, the affinities of *G. mcnamarai* remain mysterious because of its fragmentary remains.

Relationships among extant taxa are well established based on molecular analyses, and were used as backbone constraints for the phylogenetic analysis. Thus, low support for some of the clades comprising modern taxa indicates conflict in the signal from the morphological data for those clades, and there may be scope to optimize the character set for the Megapodiidae. Nevertheless, the analysis is informative about the hypothesis that modern malleefowl *L. ocellata* is a phyletic dwarf of *P. gallinacea* (including *La. naracoortensis* within the synonymy of *P. gallinacea*), with small-bodied *Leipoa* having evolved as a product of ‘Late Pleistocene dwarfing’ [24]. Monophyly of the *Progura + Latagallina* clade is well supported, and there is only very weak support (53%) for a sister taxon relationship between the *Progura* + *Latagallina* clade and *Leipoa*, so this relationship should be considered unresolved. These results provide neither support for a close relationship of *L. ocellata* to the fossil taxa, nor for an ancestral relationship between any of them and *L. ocellata* as the dwarfing idea implies. In fact, numerous morphological differences throughout the skeleton differentiate the extinct taxa from *Leipoa*. Furthermore, we have provided fossil evidence that small-bodied *Leipoa* had evolved by the Early Pleistocene at the latest, thus dwarfing in the Late Pleistocene is highly improbable. There is currently no fossil evidence to contradict an estimated divergence date of the *Leipoa* lineage from other members of the brush-turkey clade in the Miocene [4].

Despite the apparent absence of dwarfing in the *Leipoa* lineage, we see evidence for body-size flexibility in other megapode lineages. The extinct Australian genera *Progura* and *Latagallina* each contained one ‘giant’ and one somewhat smaller, though still larger than modern, species. Marked size variation has been identified in the scrubfowl clade as well: large body size evolved within the genus *Megapodius* on islands [6,14,15]. However, the smallest extant species of *Megapodius*, *M. pritchardii*, is also an island species, so this genus offers few clues as to the likely direction of body size trends in the extinct Australian lineages, particularly in a continental context.

### 4.3. Body mass

Previous estimates of body mass for *P. gallinacea*, the largest known species of megapode, are invalid because they were based on skeletal material that does not belong to this species, or were based on possibly erroneous measurements. The original estimated mass for this species was 5–7 kg, based on the cube of the length of the coracoid AM F54720 from Wombeyan Caves, New South Wales [23], but we have now referred this specimen to *La. naracoortensis*. Later estimates for *P. gallinacea* [24] of 7.8–10.1 kg were also based on material from Naracoorte that we have now referred to *La. naracoortensis.* Furthermore, we could not replicate the measurements on which those estimates were based, regardless of taxon, and have no data to indicate that any of the extinct Australian megapodes weighed as much as 10 kg.

Our estimate for *P. gallinacea* of 7.7 kg (table 15), based on minimum shaft width of the lectotype tarsometatarsus from the Darling Downs (QM F1143), is therefore the first published estimate based on material referrable to this species. A mass of nearly 8 kg for *P. gallinacea* is perhaps smaller than expected, given that bones of this species are in general considerably larger than those of *P. campestris*, *La. naracoortensis* and *G. mcnamarai*, which all weighed in the region of 5 or 6 kg using our preferred equation for the tibiotarsus (table 15). Minimum tarsometatarsus width correlates less well with body mass than some other skeletal measurements, though [43], and the estimate for *P. gallinacea* should therefore be regarded as indicative only. Nevertheless, it is possible that this species was truly not much heavier than *P. campestris* or *La. naracoortensis*, with its long, distally tapered tarsometatarsus meaning that *P. gallinacea* stood exceptionally tall but was not a disproportionately bulky animal. Despite being the largest known species of megapode, *P. gallinacea* does not show signs of a reduced pectoral girdle indicative of flightlessness*.* It may have flown as well as the heaviest extant galliform, wild turkey *Meleagris gallopavo* of North America*,* males of which have an almost identical mean body mass of 7.8 kg [53]. Similarities in their body masses may indicate that *M. gallopavo* and *P. gallinacea* approached the mass threshold for the retention of flight in galliforms.

Our preferred body mass estimates for *P. campestris*, *La. naracoortensis* and *G. mcnamarai*, based on minimum circumference of the tibiotarsus, indicate that these taxa had overlapping body masses (*P. campestris*, 6.2 kg estimated from holotype; *La. naracoortensis*, mean 5.2 kg, range 3.7–6.5 kg, calculated from 13 specimens; *G. mcnamarai*, calculated from paratype tibiotarsus). The heaviest individual of *La. naracoortensis* had nearly twice the estimated mass of the smallest individual. All measurements were on skeletally mature individuals, so such a difference might indicate sexual dimorphism in this species. Assessing this was beyond the scope of this taxonomic study, but could be investigated in future research, as the large number of skeletal elements preserved for this species would allow statistical interrogation.

At 2.9 kg, our preferred estimate for *La. olsoni*, based on circumference of the tibiotarsus, equalled that of the heaviest recorded extant megapode, a male individual of *Al. lathami* [53], but is above the mean for that species (*Alectura* females 2.2 kg, males 2.5 kg [53]). *Latagallina olsoni* was also heavier than Australia's other extant megapodes, being around two-and-a-half times heavier than *M. reinwardt* (females 888 g, males 1.1 kg) [53] and around one-and-a-half times heavier than *L. ocellata* (females 1.8 kg, males 2.0 kg) [53]. Although *La. olsoni* had legs of similar length to *L. ocellata*, considerably greater mass of the extinct species implies that it had a much stouter body, and this is borne out by the larger pelvis and notarium of the extinct species.

In summary, the extinct Plio-Pleistocene megapodes spanned an estimated mass range from 2.9 to 7.7 kg. This is on a par with the heaviest extant galliforms on other continents, including the curassows of the tropical Americas, turkeys of North America and the largest grouse and pheasants of Eurasia [53]. Differences in body size may imply niche partitioning between the extinct species, which could help to explain the geographical/temporal overlap of three megapode species on the Nullarbor Plain during the Early Pleistocene (*P. campestris*, *L. ocellata* and *La. olsoni*) and two at Naracoorte during the Middle to Late Pleistocene (*La. naracoortensis* and *L. ocellata*). However, exact contemporaneity of species is difficult to establish, as the fossil deposits accumulated over periods of thousands of years.

### 4.4. Body proportions and flight ability

We note strong similarity between the skeletal proportions of congeners *La. naracoortensis* and *La. olsoni* (figure 22). This is despite their very different body sizes, and reinforces the conclusions of our anatomical observations and phylogenetic analysis that they are closely related. There is little allometric scaling despite *La. naracoortensis* approaching double the mass of *La. olsoni* (table 16), demonstrating that body proportions within a megapode lineage can be independent of overall size.

Such similarities may be useful for testing any further apparent examples of gigantism/dwarfing within the megapodes. Pertinent to the question of whether *L. ocellata* is a phyletic dwarf of one of the extinct taxa, the proportions of that species are rather dissimilar to those of *La. naracoortensis*, *La. olsoni* and *P. campestris* are (figure 22). *Progura gallinacea* was not included in the Simpson log-ratio diagram, but dissimilarity in proportions of the tarsometatarsus of *L. ocellata* and *P. gallinacea* are evident (appendix A). The balance of evidence, including the anatomical differences that we have documented, the results of our phylogenetic analysis, occurrence of *Leipoa* fossils in the same deposits as extinct taxa, and proportional differences revealed by the Simpson diagram, is that *L. ocellata* does not belong to the same genus as any of the extinct taxa, and nor can it be descended from any of them.

The Simpson diagram (figure 22) and our morphological observations indicate that the included fossil species were volant, with strong pectoral girdles. The pectoral elements are not atrophied as they are in the extinct flightless stem-galliform *Sylviornis* [21]. *Garrdimalga mcnamarai* was not included in the Simpson diagram because its remains are so fragmentary, but it is likely that it, too, was volant given its proportionally long carpometacarpus relative to hind limb bone size (see Diagnosis).

### 4.5. Anatomy and ecology

The extinct taxa share some anatomical traits that may be associated with similarities in ecology and life history. Most notably, none of the four extinct Plio-Pleistocene taxa for which phalanges/unguals are preserved appears to have been specialized for mound-building: *P. campestris*, both species of *Latagallina*, and *G. mcnamarai* all have comparatively short, stout phalanges and claws that are lateromedially compressed and deeply curved rather than dorsoventrally flattened as in extant mound-building species (e.g. *Leipoa*). They are similar to the proportionally short and laterally compressed unguals seen in burrow-nesting megapodes such as *Ma. maleo*, which digs in sand, and the stem-galliform *S. neocaledoniae*, for which there is no evidence of egg-burying behaviour [21].

In addition, some of the extinct taxa show further signs of adaptation away from mound-building, notably a short, shallow fossa parahypotarsalis medialis on the tarsometatarsus in both species of *Progura*, and proportionally shorter toes than those of specialist mound-builders (figure 11). If our interpretation is correct and these taxa were all burrow-nesters, this would mean that burrow-nesting, which is a state currently only present in extant members of the ‘scrubfowl’ clade [4], evolved or re-evolved independently within the Australian ‘brush-turkeys’.

An ancestral state reconstruction using Bayesian analysis [4] determined that mound-building was the most probable ancestral nesting strategy in the megapodes, with burrow-nesting having evolved three times within the scrubfowl clade. That analysis was based on the prior that all members of the brush-turkey clade are mound-builders. However, if, as our study suggests, some brush-turkeys were burrow-nesters, repeating the ancestral state reconstruction with burrow-nesting taxa within the brush-turkey clade could produce more equivocal results. Note also that the sister taxon of all galliforms, *S. neocaledoniae*, was determined to be a non-mound-builder and almost certainly an endothermic incubator [21], and therefore, ectothermic incubation is a synapomorphy of megapodes.

#### 4.5.1. Progura gallinacea

Relatively few elements of *P. gallinacea* are known (see species description), limiting interpretation of its functional anatomy. No cranial remains of this species are known and so nothing can be deduced about its feeding habits or its sensory abilities. Despite its limited remains, *P. gallinacea* is clearly the largest known species of megapode, extant or extinct. The tarsometatarsus, reconstructed to have been nearly 150 mm long, is of similar length to that of the giant flightless stem-galliform *S. neocaledoniae* from the Pacific [21], but unlike *Sylviornis*, the bones of the pectoral girdle of *P. gallinacea*, insofar as they are preserved, show no signs of reduction: the carpometacarpus, proximal scapula and coracoid are not gracile and have strong muscle attachments. We therefore presume that adults of this species could fly, even if only for short distances, and could have flown up into trees to roost as do extant megapodes [3].

No phalanges or unguals of *P. gallinacea* are known, so it is more difficult to deduce the digging/nesting behaviour of this species compared with the other extinct species. However, the relatively small fossa parahypotarsalis medialis on the tarsometatarsus suggests that the musculature for the toes was reduced, which we consider to be a sign that this species was not particularly well adapted for using its feet to build mounds. It is possible that it buried its eggs in river sands or gravels, given that its remains in southeast Queensland and in northern South Australia, its bones have been found in riverine depositional environments, but it is possibly more likely that it buried its eggs in adjacent sands or soils [12,36].

#### 4.5.2. Progura campestris

Almost the entire skeleton of *P. campestris* is known, allowing a better appraisal of its functional anatomy than for *P. gallinacea*. This species has a pectoral girdle slightly reduced relative to its hindlimb compared with similarly sized *La. naracoortensis* (figure 22). Both species weighed in the region of 5–6 kg. Therefore, slightly reduced pectoral elements suggest that *P. campestris* was a somewhat weaker flyer than *La. naracoortensis*. Typically, there is a trade-off between the size/muscle mass of the hindlimb and forelimb in birds, with longer legs thought to compensate for weaker pectoral muscles by generating increasing forward force during take-off [55]. Thus, *P*. *campestris* may have relied on its relatively more elongate tarsometatarsus to launch from the ground. Conversely, short distal legs are paired with longer wings in *La. olsoni* and *La. naracoortensis*, which may mean that these species generated greater forces with their wings/pectoral girdle to become airborne.

In *P. campestris*, the length of the femur is about equal to that of the tarsometatarsus, while in *La. naracoortensis*, the tarsometatarsi are only about 88% of femoral length. This indicates that the two species, which had similar body masses, had different biomechanical properties of the hindlimb and may have differed in locomotory style and/or digging behaviour. This could be further investigated.

Cranial anatomy of *P. campestris* differs notably from that of other megapodes, in that the lacrimals and the margo supraorbitalis flare laterally (figure 6*a*), which would have provided bony protection to the eyes. This could signify that the species foraged in tough or spiny vegetation. Also of note is the very small foramen nervi olfactorii (figure 6*c*), similar to that of *La. naracoortensis* (figure 12*c*), but dissimilar to the large and obvious foramina seen here in *L. ocellata*, *Al. lathami*, *M. reinwardt* and *Ma. maleo*. This may mean that olfaction was of decreased importance in *P. campestris*. Given that the paratype cranium is well preserved (figure 6), it should be possible to test this hypothesis by using computed tomography to reconstruct a virtual brain cast of this species and assess the size of the brain region corresponding to the processing of olfactory stimuli [56].

The tarsometatarsi of *P. campestris* and *La. naracoortensis* are of roughly similar length, but the distal end in *P. campestris* is relatively narrow, indicating it probably had a smaller foot span (figure 5). As the unguals of both species are apparently adapted away from mound-building, differences in foot size may relate to differences in digging/nesting behaviour between these species, such as different preferred substrates for egg burial.

The shape of the pygostyle in *P. campestris* suggests that this species may have had elaborate tail feathers. The bone is flexed dorsally at an approximately 45° angle (figure 9*j*) and the lamina have a large surface area for muscular attachment, rather like the pygostyle of extant silver pheasant *Lophura nycthemera* [57], which has a long trailing tail. By contrast, the pygostyle of *Al. lathami*, which is noted for its short, vertically fanned tail (the name *Alectura* = cock-tail [58]), is rather straight and lacks deep sulci on the laminae, and *L. ocellata*, which has a short tail with a downward posture, has a pygostyle that is laterally compressed and points slightly downwards.

#### 4.5.3. Latagallina naracoortensis

This was a stout, heavy, short-legged megapode, but despite this shows no signs of reduced volant ability (see above). It had long, strong wings and large, robust coracoids, larger than those of *P. campestris*, and so it may have flown more strongly.

The cranium of *La. naracoortensis* (figure 12) lacks bony protection of the eyes as is seen in *P. campestris* and it may therefore have fed in a different manner. This is further suggested by the different shapes of the beak in these species, (narrow, elongate tip in *P. campestris—*figures 6*f*–*h* and 7*d*–*f*; shorter and broad in *La. naracoortensis—*figures 7*j*–*l* and 12*e*–*g*). However, the diet of neither species is known. Despite these differences, an important similarity between *La. naracoortensis* and *P. campestris* is the small foramen for the olfactory nerve (figure 12*c*), posterior to the sulcus nervi olfactorii, meaning this species may also have had reduced olfactory capacity. This character is not captured in the character set that we used for our phylogenetic analysis, but seems to further confirm that *Progura* and *Latagallina* belong to the same clade.

The comparatively larger fossa parahypotarsalis medialis on the tarsometatarsus of *La. naracoortensis* (figure 5) could mean that this species was a more powerful digger than *P. campestris*, but its short, laterally compressed, curved unguals (figure 15*l*) would have made it poorly adapted to mound-building compared with extant taxa like *L. ocellata*. If we have correctly interpreted this species to have been a burrow-nester, it may have buried its eggs in a more resistant substrate than did *P. campestris*.

#### 4.5.4. Latagallina olsoni

As with its larger congener, *La. olsoni* had comparatively long wings and short legs (figure 22) and was probably a strong flyer. The tibiotarsus and tarsometatarsus of *La. olsoni* were shorter than in extant *L. ocellata*, but the femur was longer. This may mean that these similarly sized species differed in locomotory style and may also have differed in how they used their legs and feet for digging. Articulation of the femur with the acetabulum of the pelvis shows that the leg was able to be splayed more widely than in *L. ocellata*, due to its more proximally orientated crista trochanteris, perhaps indicating a generally wider stance in *La. olsoni* because of its greater body mass. A broad, deep pelvis could have allowed *La. olsoni* to lay larger eggs than *L. ocellata*. The only cranial remains of *La. olsoni*, the anterior portion of the mandible, shows that the angle between the left and right rami was comparatively wide (figure 18*a*,*b*), and the tip of the bill may therefore have had a similar shape to that of congener *La. naracoortensis* (figures 7*j*–*l* and 12*e*–*g*).

#### 4.5.5. Garrdimalga mcnamarai

Little can be deduced about the habits of this species due to its limited remains. However, compared with the similarly sized species *P. campestris* and *La. naracoortensis*, *G. mcnamarai* seems to have had large phalanges and unguals relative to the dimensions of its tarsometatarsi and tibiotarsi. This species may therefore have been a more powerful digger than those taxa. The humerus and most of the ulna are unknown, but the radius and carpometacarpus show that the distal wing was relatively long, and therefore, this species may have been a strong flyer. Leg length, however, is not known and so relative length of the wing and leg cannot be determined. As in species of *Latagallina*, the tip of the bill in *G. mcnamarai* was short and wide (figures 7*g*–*i* and 21*e*–*g*), rather than elongate as in *P. campestris*.

### 4.6. Extinction and the possible role of humans

Here, we have shown that the generic diversity of megapodes in Australia halved—and species diversity reduced by more than 60%—within the last few hundred-thousand years. Timing and reasons for extinction among the fossil taxa are not known, but based on current fossil evidence, no small-bodied Australian megapode species went extinct during the Pleistocene, and there appears instead to have been a preferential loss of larger, heavier species. Large body size would have been a risk factor if it was associated with slow reproductive rates or susceptibility to hunting, as proposed for Australia's Pleistocene mammalian megafaunal extinctions [59]. Nevertheless, there is currently no direct evidence that humans encountered or hunted any of the species in question. Pertinent to the issue of hunting, all of the extinct megapodes appear to have been able to fly (see above), and thus, they may have been less vulnerable to hunting than might be supposed based on size alone. Given that at least four of the five species of megapode belonged to a single clade (see Phylogenetic analysis), it is also possible that other shared derived life-history or behavioural traits, such as diet or nesting behaviour, may have rendered them vulnerable to extinction.

Although evidence for hunting of adult birds is absent, fossil eggshell from various parts of Australia does indicate that humans consumed the eggs of at least one large extinct species of megapode during the Late Pleistocene. Eggshell fragments with characteristic burn patterns indicate that these eggs were cooked and consumed by people in various parts of Australia [60,61]. So far, these eggs have not been attributed to any species in particular. Fossil eggshell of this type, formerly attributed to the giant extinct galloanseriform *Genyornis newtoni* [61,62], but since identified as belonging to one or more species of megapode [63], disappeared from the fossil record around 47 ka, within a few thousand years of first human arrival in Australia [64,65]. All of the fossil bones that we have described are considerably older than 47 kyr, so it is unknown if one or more of these taxa survived late enough into the Pleistocene to have been encountered by people. However, all the burnt eggshell material has been recovered from sandy environments, so given that we believe that all the large extinct taxa were burrow-nesters rather than mound-builders, the eggs could plausibly belong to one or more species of *Latagallina*, *Progura*, *Garrdimalga* or perhaps other extinct taxa as yet unknown.

Egg size narrows down the possible candidate species for having laid these eggs. The best-preserved specimen, the ‘Spooner Egg’ (SAM P42421), was collected from dunes at the head of the Spencer Gulf in South Australia and was dated by optically stimulated luminescence to be 54.7 ± 3.1 kyr old [63]. The volume of the Spooner Egg is roughly equivalent to that of the extant emu *Dromaius novaehollandiae*, but is shorter and broader [63]. Although some still dispute its identity as a megapode egg [66], the Spooner Egg, and other similar eggshell material from multiple localities, originally referred to *Ge. newtoni* [62], is almost certainly too small and too thin to have belonged to the giant stem-galliform *Genyornis* [67]. Extant female megapodes lay exceptionally large eggs relative to body size [68], and although the Spooner Egg is very large compared with eggs of extant megapodes, it was calculated that it would not be unusually long for a female megapode of approximately 5 kg in mass [63,67]. Our body mass estimates for extinct megapodes (table 16) place two candidate species in this range (*La. naracoortensis*, mean 5.2 kg; *G. mcnamarai*, 5.2 kg), and two somewhat heavier (*P. campestris,* 6.2 kg; *P. gallinacea*, 7.7 kg). Thus, there is circumstantial evidence that at least one of these very large species could have survived until human arrival in the Late Pleistocene, only to then become extinct.

The Spooner Egg is notably broad for its length [63], which may indicate it was laid by a megapode with an especially large pelvis [66], given that egg size in birds is constrained by pelvis size [69]. A proportionally long and broad pelvis was noted in *La. olsoni* compared with *L. ocellata*, despite these species having similar leg length (see species description), and thus *La. olsoni* probably laid larger eggs than a malleefowl. With an estimated body mass of 2.9 kg (table 16), *La. olsoni* is too small to have laid the Spooner Egg based on a 5 kg estimate for its parent bird [63]. However, *La. olsoni*'s much larger congener *La. naracoortensis* probably also had a proportionally large pelvis, based on very similar body proportions of these two species throughout the rest of the post-cranial skeleton (figure 21), and thus *La. naracoortensis* would be a plausible parent bird for the fossil eggshell. Based on fossils examined in this study, some skeletal remains of *La. naracoortensis* from Naracoorte are from deposits of likely Late Pleistocene age, thus it is probably the latest-known surviving species among our five extinct taxa. Future studies may elucidate whether the geographically widespread Pleistocene megapode eggshells derive from one or multiple species, and help clarify the geographical distribution and extinction history of large megapodes even in locations where fossil bones are absent.

### 4.7. Geographical and temporal distribution

Two of the species we have described, *P. campestris* and *La. olsoni*, are both known only from the Nullarbor Plain, south-central Australia, while *G. mcnamarai* is only known from Yorke Peninsula in southern South Australia. These species may have had very limited geographical distributions, but it seems more likely that their remains are yet to be located further afield.

The known range of *P. gallinacea* has altered as a result of this study. We have confirmed its presence in riparian habitats both on the Darling Downs in southeastern Queensland and on the Warburton River in the Lake Eyre Basin, northern South Australia. Similarities have previously been noted between the Pliocene marsupial faunas of the Tirari Formation in the Lake Eyre Basin, and the Chinchilla Sand on the Darling Downs [12,36], so the presence of *P. gallinacea* at both localities is further evidence of their faunal similarity. ‘*Progura’ naracoortensis* (within the synonymy of *P. gallinacea*) was previously thought to also be present at Bluff Downs [11], and at several sites in eastern New South Wales [23,24]. However, we consider the Bluff Downs megapode fossils to be far too small to be *P. gallinacea*, and having examined the fossils from New South Wales, we have referred these to *La. naracoortensis*. At present, then, *P. gallinacea* is known only from two localities in Australia's mid-latitudes, one relatively close to the east coast and the other deep in the interior.

On current evidence, *La. naracoortensis* had the widest geographical distribution of the extinct species. To this taxon, we have referred specimens from Naracoorte and Mt Gambier in southeastern South Australia, as well as three localities in eastern New South Wales and one in southeast Queensland. Thus far, *La. naracoortensis* appears to have been a species of the southeastern quarter of Australia. No fossils of this species are known from Victoria, but it seems likely to have inhabited the region intermediate between eastern New South Wales and southeastern South Australia. In each known locality, it has been recovered from limestone caves or fissure fills in limestone, but this may be the result of preservation bias and may not indicate its true distribution or preferred substrate.

Further to the extinct taxa, we have also documented Pleistocene fossils of extant *L. ocellata* from two southern localities: the Thylacoleo Caves of south-central Australia; and the Naracoorte Caves of southeastern South Australia. These occurrences are in a similar geographical zone to where the species occurs today, perhaps indicating relative stability in the distribution of malleefowl since the Pleistocene. The occurrence of *L. ocellata* in the heart of the Nullarbor Plain during the Early Pleistocene, however, is a modest range extension compared with today. There are no modern records of *L. ocellata* from the Nullarbor Plain, although the species is recorded from woodlands peripheral to the Plain [70].

An implication of our findings for future palaeontological studies is that the identity of Australian fossil megapodes cannot safely be deduced from age, size and geography alone: identification based on morphology is essential. Just as the ranges of *M. reinwardt* and *Al. lathami* overlap in northeastern Australia today, fossil evidence shows that *L. ocellata*, *P. campestris* and *La. olsoni* all inhabited the Nullarbor Plain during the Early Pleistocene. Furthermore, on the Darling Downs in southeast Queensland, *P. gallinacea* and *La. naracoortensis* are present as fossils in the Pleistocene, and at Naracoorte *L. ocellata* and *La. naracoortensis* occur in the same Pleistocene deposits. The degree of temporal overlap between any of these species at a given location is uncertain, but spatial overlap is indisputable.

So far, only one species of megapode—*P. gallinacea*—has been confirmed from sites of Pliocene age. Although additional species from this epoch may yet be discovered, and the Bluff Downs fossils require reappraisal, current evidence indicates that megapode diversity reached its peak in Australia in the Pleistocene. During this epoch, there is direct fossil evidence for four genera (*Leipoa*, *Progura*, *Latagallina* and *Garrdimalga*), and five species (*L. ocellata*, *P. gallinacea*, *P. campestris*, *La. naracoortensis*, *La. olsoni* and *G. mcnamarai*), and *Al. lathami* and *M. reinwardt* were also presumably resident.

### 4.8. Palaeoenvironments

This previously obscure diversity among the megapodes implies that the Pleistocene environment of Australia was particularly favourable for these mainly ground-dwelling birds. Occurrence of two or more species at three localities implies niche partitioning may have been at play, but as yet we know very little about how any of the extinct megapodes used their habitats.

Burrow-nesting is thought to have evolved in some species of *Megapodius* in response to their dispersal by flight into new habitats [1], in their case tropical islands with sandy substrates. In continental Australia, the rise of a burrow-nesting clade of *Progura* and *Latagallina* (and *Garrdimalga* if this genus also belonged to the same clade) could have been an evolutionary response to the spread of sandy habitats due to global trend towards aridification during the Plio-Pleistocene [71,72]. However, at Chinchilla and on the Warburton River *P. gallinacea* inhabited riparian environments, seemingly at odds with aridification. The Pliocene habitat at Chinchilla has been interpreted as a mix of tropical forest, wetlands and grasslands, with estimated annual rainfall of around 1000 mm [12,73]. The Pliocene environment of the Tirari Formation in the Lake Eyre Basin, on the other hand, while also riparian, has been described as highly evaporative [36]. Thus, the habitat and climatic tolerances of *P. gallinacea* are ambiguous.

Megapodes are omnivores, thus diet offers few clues for reconstructing the palaeoenvironments of the extinct taxa. However, it is likely that all the fossil megapodes required trees for roosting and for escaping predators, as with extant species [3]. Given that several of the fossil taxa weighed upwards of 5 kg, large trees were probably necessary to support their weight. Fossils of extant *L. ocellata* are somewhat more informative about local Pleistocene habitats at Naracoorte and on the Nullarbor Plain because this species is a known habitat specialist of low open woodland, comprising in almost all cases an overstorey of multi-stemmed *Eucalyptus* trees, which provide the leaf litter with which it builds its nest mounds [3]. The local Pleistocene environment around the Thylacoleo Caves has previously been interpreted as a mosaic of grassland and woodland, based mainly on composition of the marsupial fossil fauna [28]. The addition of *L. ocellata* to the species list for this locality corroborates the conclusion that the Nullarbor Plain, which today completely lacks a tree canopy, was at least at times an open woodland environment in the first half of the Pleistocene.

## 5. Conclusion

The trend in taxonomy over the last three decades has been towards a reduction in the number of extinct megapode taxa recognized from the Australian Cenozoic. First was the synonymy of *P. gallinacea* and *P. naracoortensis*, then the suggestion that *P. gallinacea* was the megafaunal antecedent of modern *L. ocellata*. The discovery of new fossil material from the Nullarbor Plain, Curramulka Quarry and the Warburton River has provided fresh perspective from which to re-evaluate previously known fossils from Naracoorte, New South Wales and southeast Queensland. As a result, this study has revealed that there was a previously unknown radiation—comprising three extinct genera and five extinct species—within the brush-turkey clade in Plio-Pleistocene Australia. All species appear to have been burrow-nesters rather than mound-builders, a behaviour previously unknown among the brush-turkeys.

Here, we have demonstrated that *P. gallinacea* and ‘*Progura*’ *naracoortensis* are generically distinct from each other, although they do belong in a single clade. Both these taxa differ from *L. ocellata*, which was roughly contemporaneous with several larger taxa. We have found no evidence to support the claim that *L. ocellata* is a phyletic dwarf. Evolutionary relationships of *G. mcnamarai* remain to be determined. As a result of a clarification of taxonomy, we conclude that generic diversity of megapodes in Australia has halved in the last few hundred-thousand years, and species diversity has reduced by more than 60%, a startling loss. We hope that these discoveries provide perspective on the historical importance of these birds in the evolution and ecology of Australia. We hope also that our study prompts a renewed appreciation for the vulnerability of megapodes to the forces of extinction, and the need to conserve the species that still survive.

## Supplementary Material

Megapode matrix

## Supplementary Material

Phylogenetic characters

## Supplementary Material

Supplementary Figure1: Type material of Garrdimalga mcnamarai sp. nov. compared with other extinct species

## Figures and Tables

**Figure 24.**
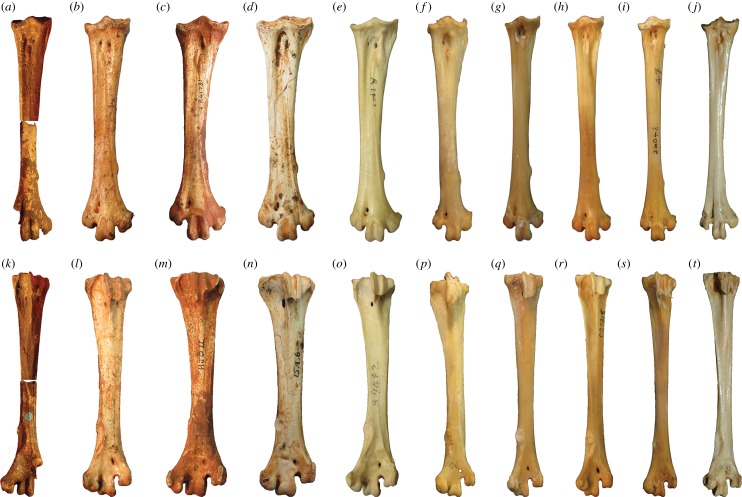
Tarsometatarsi of extinct and extant megapodes in dorsal (top row) and plantar aspects (bottom row), scaled to the same length to reveal divergent proportions. *Progura gallinacea* (*a*,*k*) (reconstructed from two fragments); *P. campestris* sp. nov. (*b*,*l*); *La. naracoortensis* (*c*,*m*); *La. olsoni* sp. nov. (*d*,*n*); *L. ocellata* (*e*,*o*); *M. reinwardt* (*f*,*p*); *T. fuscirostris* (*g*,*q*); *Al. lathami* (*h*,*r*); *Ae. arfakianus* (*i*,*s*); *Ma. maleo* (*j*,*t*).

## Data accessibility

All data are included in the manuscript, appendices or have been uploaded as part of the electronic supplementary material.

## Appendix A

Tarsometatarsi of extinct and extant megapodes (figure 24).

## Appendix B

***Latagallina naracoortensis* specimens from the Naracoorte region examined for this study: specimen numbers and measurements**

Crania

Henschke's Fossil Cave: SAM P22713; SAM P31773; SAM P31774; SAM P41861. Komatsu Cave: SAM P51369. Victoria Fossil Cave: SAM P41535; SAM P41536.

Premaxillae

Henschke's Fossil Cave: SAM P22871; SAM P23128; SAM P41397.

Notaria

Henschke's Fossil Cave: SAM P18186; SAM P22869; SAM P41687.

Pelves and synsacra

Buckridge Cave: SAM P51236; SAM P51237; SAM P51250; SAM P51355; SAM P51257; SAM P51258; SAM P51261. Henschke's Fossil Cave: SAM P18187; SAM P18635; SAM P22818; SAM P22819; SAM P22856; SAM P23117; SAM P23121; SAM P23154; SAM P23352; SAM P23370; SAM P23412; SAM P23413; SAM P23414; SAM P24146; SAM P24191; SAM P41366; SAM P41373; SAM P41688; SAM P41716; SAM P41845. Victoria Fossil Cave: SAM P41563; SAM P41564.

Sterna

Henschke's Fossil Cave: SAM P23125; SAM P23302; SAM P23323; SAM P23337; SAM P24190; SAM P39620; SAM P41699; SAM P41700; SAM P41751; SAM P41828; SAM P41857. Victoria Fossil Cave: SAM P16162; SAM P27528; SAM P28005; SAM P41545.

Furculae

Buckridge Cave: SAM P51259; SAM P51260. Henschke's Fossil Cave: SAM P23131; SAM P23149; SAM P41846.

Coracoids

Buckridge Cave: SAM P51245; SAM P51251; SAM P51352; SAM P51354; SAM P51364. Fox Cave: SAM P19027; SAM P19100. Henschke's Fossil Cave: SAM P18628; SAM P19071; SAM P19073; SAM P19076; SAM P22715; SAM P22817; SAM P22820; SAM P22858; SAM P22868; SAM P22872; SAM P23119; SAM P23120; SAM P23130; SAM P23336; SAM P24144; SAM P24161; SAM P24167; SAM P24172; SAM P24173; SAM P24187; SAM P24188; SAM P24189; SAM P24204; SAM P24212; SAM P39636; SAM P39637; SAM P39638; SAM P39645; SAM P39650; SAM P41486; SAM P41582; SAM P41586; SAM P41587; SAM P41588; SAM P41603; SAM P41609; SAM P41652; SAM P41653; SAM P41654; SAM P41657; SAM P41663; SAM P41664; SAM P41718; SAM P41733; SAM P41734; SAM P41735; SAM P41831; SAM P41840; SAM P41842. Victoria Fossil Cave: SAM P16700; SAM P P41538; SAM P41554.

Scapulae

Fox Cave: SAM P17373. Henschke's Fossil Cave: SAM P18630; SAM P19077; SAM P22863; SAM P23281; SAM P23408; SAM P23409; SAM P39634; SAM P39635; SAM P39648; SAM P39652; SAM P41374; SAM P41392; SAM P41481; SAM P41578; SAM P41591; SAM P41594; SAM P41657; SAM P41660; SAM P41696; SAM P41717; SAM P41730; SAM P41856; SAM P41686. Victoria Fossil Cave: SAM P41543.

Humeri

Buckridge Cave: SAM P51346; SAM P51347; SAM P51348; SAM P51350; SAM P51353; SAM P51363; SAM P51359. Henschke's Fossil Cave: SAM P17153; SAM P17154; SAM P17878; SAM P18183; SAM P18625; SAM P18627; SAM P18638; SAM P22714; SAM P22716; SAM P22719; SAM P22851; SAM P22865; SAM P23118; SAM P23133; SAM P23283; SAM P23293; SAM P23296; SAM P23297; SAM P23327; SAM P23374; SAM P23442; SAM P24143; SAM P24147; SAM P24148; SAM P24153; SAM P24159; SAM P24174; SAM P24175; SAM P24176; SAM P24202; SAM P24209; SAM P24210; SAM P24211; SAM P24219; SAM P39625; SAM P39629; SAM P39621; SAM P39639; SAM P41364; SAM P41367; SAM P41483; SAM P41648; SAM P41649; SAM P41650; SAM P41683; SAM P41684; SAM P41697; SAM P41714; SAM P41727; SAM P41728; SAM P41743; SAM P41750; SAM P41830; SAM P41833. Victoria Fossil Cave: SAM P41540; SAM P41551; SAM P41552; SAM P41553; SAM P42076; SAM P42706. Wombat Cave: SAM P18636.

Ulnae

Buckridge Cave: SAM P51269; SAM P51270; SAM P51271; SAM P51272; SAM P51273; SAM P51343; SAM P51344; SAM P51345. Henschke's Fossil Cave: SAM P17817; SAM P17877; SAM P17879; SAM P18626; SAM P19069; SAM P19075; SAM P19079; SAM P23115; SAM P23126; SAM P23127; SAM P23132; SAM P23282; SAM P23284; SAM P23294; SAM P23375; SAM P24157; SAM P24160; SAM P24164; SAM P24178; SAM P24179; SAM P24180; SAM P24181; SAM P24182; SAM P39626; SAM P39627; SAM P39649; SAM P41363; SAM P41394; SAM P41589; SAM P41611; SAM P41612; SAM P41647; SAM P41658; SAM P41661; SAM P41678; SAM P41679; SAM P41680; SAM P41681; SAM P41711; SAM P41712; SAM P41713; SAM P41736; SAM P41752; SAM P41836. Kilsby's Hole (Mt Gambier): SAM P42079. Victoria Fossil Cave: SAM P41544.

Radii

Henschke's Fossil Cave: SAM P18099; SAM P18184; SAM P18629; SAM P18632; SAM P23376; SAM P23377; SAM P39640; SAM P39642; SAM P41585; SAM P41597; SAM P41599; SAM P41742; SAM P41922; SAM P41929.

Carpometacarpi

Buckridge Cave: SAM P51356; SAM P51357; SAM P51358; SAM P51362; SAM P51365; SAM P51366. Fox Cave: SAM P17364; SAM P17369. Henschke's Fossil Cave: SAM P22862; SAM P22870; SAM P23116; SAM P23110; SAM P23129; SAM P23324; SAM P23410; SAM P23443; SAM P24139; SAM P24140; SAM P24171; SAM P24183; SAM P24184; SAM P24203; SAM P24213; SAM P39628; SAM P39630; SAM P39631; SAM P39632; SAM P39641; SAM P39651; SAM P41583; SAM P41606; SAM P41584; SAM P41726; SAM P41655; SAM P41695. Victoria Fossil Cave: SAM P41537; SAM P28006.

Femora

Buckridge Cave: SAM P51244; SAM P51246; SAM P51247; SAM P51248; SAM P51249; SAM P51253; SAM P51254; SAM P51255; SAM P51262; SAM P51263; SAM P51351. Fox Cave: SAM P17366. Henschke's Fossil Cave: SAM P17857; SAM P18186; SAM P19068; SAM P19070; SAM P19074; SAM P19078; SAM P22712; SAM P22852; SAM P22854; SAM P23111; SAM P23112; SAM P23122; SAM P23134; SAM P23153; SAM P23325; SAM P23340; SAM P23371; SAM P23379; SAM P23445; SAM P24149; SAM P24165; SAM P24192; SAM P24205; SAM P39622; SAM P39633; SAM P41361; SAM P41362; SAM P41393; SAM P41476; SAM P41480; SAM P41482; SAM P41493; SAM P41494; SAM P41592; SAM P41593; SAM P41604; SAM P41682; SAM P41719; SAM P41729; SAM P41741; SAM P41834. Victoria Fossil Cave: SAM P41541; SAM P41549; SAM P41550; SAM P42707.

Tibiotarsi

Buckridge Cave: SAM P51238; SAM P51239; SAM P51240; SAM P51241; SAM P51242; SAM P51243. Fox Cave: SAM P17365; SAM P19042; SAM P19098. Henschke's Fossil Cave: SAM P17152; SAM P17876; SAM P18633; SAM P18792; SAM P23123; SAM P23152; SAM P23285; SAM P23286; SAM P23295; SAM P23298; SAM P23299; SAM P23326; SAM P23381; SAM P24142; SAM P24150; SAM P24151; SAM P24162; SAM P24193; SAM P24194; SAM P24196; SAM P24197; SAM P24198; SAM P24206; SAM P31775; SAM P31776; SAM P39623; SAM P39624; SAM P41368; SAM P41372; SAM P41391; SAM P41396; SAM P41475; SAM P41477; SAM P41659; SAM P41690; SAM P41691; SAM P41692; SAM P41694; SAM P41720; SAM P41721; SAM P41722; SAM P41723; SAM P41724; SAM P41725; SAM P41737; SAM P41749; SAM P41835. Victoria Fossil Cave: SAM P41539; SAM P41547; SAM P41548. No location data: SAM P24195.

Tarsometatarsi

Buckridge Cave: SAM P51231; SAM P51232; SAM P51233; SAM P51234; SAM P51235; SAM P51349. Fox Cave: SAM P17361; SAM P17362; SAM P17363; SAM P17370; SAM P17371; SAM P19044. Henschke's Fossil Cave: SAM P17856; SAM P18185; SAM P19072; SAM P19099; SAM P22853; SAM P22866; SAM P22873; SAM P23109; SAM P23113; SAM P23124; SAM P23135; SAM P23287; SAM P23300; SAM P23301; SAM P23341; SAM P23382; SAM P23383; SAM P23384; SAM P23444; SAM P23446; SAM P23448; SAM P24152; SAM P24158; SAM P24199; SAM P24200; SAM P24201; SAM P24207; SAM P31772; SAM P31777; SAM P31778; SAM P31779; SAM P39643; SAM P39644; SAM P39646; SAM P39647; SAM P41479; SAM P41576; SAM P41577; SAM P41579; SAM P41600; SAM P41605; SAM P41607; SAM P41610; SAM P41685; SAM P41708; SAM P41709; SAM P41710; SAM P41731; SAM P41732; SAM P41740; SAM P41745; SAM P41746; SAM P41747; SAM P41748; SAM P41832; SAM P41837; SAM P41841; SAM P41843. Victoria Fossil Cave: SAM P25633; SAM P28007; SAM P41534.

Pedal phalanges and unguals

Henschke's Fossil Cave: SAM P23436; SAM P24156. Kilsby's Hole (Mt Gambier): SAM P42733.

Vertebrae

Buckridge Cave: SAM P51264; SAM P51265; SAM P51266; SAM P51267; SAM P51268. Henschke's Fossil Cave: SAM P18181; SAM P22821; SAM P22867; SAM P23358; SAM P24166; SAM P24169; SAM P41485.

Associated skeletons

Big Bird Cave: SAM P51368 (R femur, R ulna, R humerus, pR scapula, scapula fragment, pL tibiotarsus, fibula, five vertebrae, phalanx dig. major, phalanx dig. minor, synsacrum fragments, dL carpometacarpus, six pedal phalanges, rib fragment, sternum fragment, L coracoid sternal end).
Komatsu Cave: SAM P51369 (neurocranium, three vertebrae, synsacrum incomplete, pL scapula, L coracoid, R coracoid, dR femur, dL tarsometatarsus, pL humerus, pR humerus).
Henschke's Fossil Cave: SAM P51370 (R ulna, dL ulna, dR tarsometatarsus, dL tarsometatarsus, three coracoid fragments, seven prox. phalanges, three distal phalanges, metatarsal 1, tibiotarsus distal fragment, R humerus incomplete, pL humerus, R femur, L femur, pR carpometacarpus, L carpometacarpus incomplete, R scapula, pL scapula, various fragments).

**Measurements of *Latagallina naracoortensis* specimens (mm) (TL, total length; PW, proximal width; SW, shaft width; DW, distal width)**

*Humeri*

**Table:**

SAM #	TL	PW	SW	DW
SAM P51347	149.9	36.2	15.6	31.1
SAM P51348	—	38.3	14.9	—
SAM P51346	—	—	—	30.2
SAM P51368	151.2	37.7	16.0	30.7
SAM P51369	—	40.3	—	—
SAM P41552	135.5	—	14.4	27.2
SAM P41551	149.4	36.2	15.2	29.2
SAM P39629	—	—	—	31.6
SAM P42706	—	35.9	—	—
SAM P39639	—	—	14.2	29.6
SAM P42076	—	—	13.9	30.1
SAM P39625	142.4	35.7	15.3	28.6
SAM P41727	—	35.6	—	—
SAM P41750	—	38.6	—	28.7
SAM P17154	—	—	—	31.5
SAM P17153	143.4	35.6	14.5	29.6
SAM P24219	—	35.2	—	—
SAM P41714	—	—	14.4	28.6
SAM P23296	—	—	—	29.4
SAM P24147	—	—	—	32.7
SAM P23283	—	—	—	29.5
SAM P18636	—	—	16.1	32.7
SAM P41649	—	—	13.8	—
SAM P41648	139.4	33.6	14.5	30.0
SAM P24143	—	36.9	15.7	—
SAM P24202	—	—	—	29.0
SAM P23133	—	—	—	29.4
SAM P22714	—	—	15.3	32.1
SAM P18625	—	39.0	—	—
SAM P24175	—	—	—	29.2
SAM P23442	—	35.0	—	—
SAM P22716	—	—	14.0	29.8
SAM P24210	—	34.1	—	—
SAM P24153	—	—	—	30.3
SAM P41650	148.9	36.9	15.0	30.6
SAM P23118	—	34.3	14.5	—
SAM P23327	150.4	35.6	15.8	30.2
SAM P22719	138.4	35.5	14.3	28.8

*Ulnae*

**Table:**

SAM #	TL	PW	SW	DW
SAM P51269	165	19.8	11.5	20.6
SAM P51272	—	20.1	11.6	—
SAM P51273	—	19.4	10.6	—
SAM P51345	—	—	11.1	19.9
SAM P51343	—	19.2	11.3	—
SAM P51368	159	20.4	11.8	20.5
SAM P51270	173.9	22.6	12.6	21.5
SAM P41752	—	18.5	11.2	—
SAM P41679	—	—	—	20.6
SAM P41680	152.4	19.1	11.6	19.4
SAM P41712	—	—	—	19.5
SAM P41711	144.8	18.2	10.2	18.3
SAM P41713	—	17.4	—	—
SAM P24179	—	17.9	10	—
SAM P41658	—	18.6	—	—
SAM P41612	—	18	—	—
SAM P19075	—	—	11.4	20
SAM P23294	—	—	9.4	18.1
SAM P41544	152.4	19.2	12	19.7
SAM P39627	149	19.2	11.1	18.3
SAM P39626	—	—	11.1	21.1
SAM P41589	—	17.2	—	—
SAM P41363	—	—	11	—
SAM P42079	—	19.3	—	—
SAM P24178	—	18	—	—
SAM P23115	150.4	—	10	19.4
SAM P23282	—	—	10.4	19.5
SAM P23132	145.6	16.2	10.3	18.2
SAM P41836	—	—	—	20.4
SAM P24181	—	—	—	18.2
SAM P24160	—	—	—	18.6
SAM P24180	—	17.4	—	—
SAM P19079	—	18.9	—	—
SAM P18626	153.2	18.2	10.2	19.1
SAM P19069	150.3	17.3	10.1	17.7
SAM P17879	—	—	—	19.3

*Carpometacarpi* (^a^MW, max. width at the arch of os metacarpale minus)

**Table:**

SAM #	TL	PW	MW^a^	DW
SAM P51368	—	—	16.8	—
SAM P51367	—	25.8	—	—
0680	90.8	25.7	16.5	—
SAM P51358	85.7	25.3	17	—
SAM P51362	86.1	24.5	16.1	20.5
SAM P51357	87.3	25.7	17	—
SAM P23129	82.5	22.8	16.5	—
SAM P41584	—	21.2	—	—
SAM P17369	—	23.4	—	—
SAM P41726	—	—	15.5	—
SAM P23116	78.4	19.8	13.7	—
SAM P41655	77.3	22.7	14.2	—
SAM P41655	79.6	22.5	15.6	16.6
SAM P41695	75.7	22.1	14.4	—
SAM P39628	74.8	20.8	14.9	18.6
SAM P39631	74.8	20.8	14.9	19.5
SAM P39641	78.9	22.6	14.2	—
SAM P39632	80.9	—	14.1	—
SAM P39630	74.6	22.2	13.8	—
SAM P39651	—	22.3	—	—
SAM P41583	78.8	—	15.7	—
SAM P24184	77.1	—	14.8	—
SAM P41537	85.7	24.1	16.3	—
SAM P24213	79	20.6	14.7	19.2
SAM P23443	74.1	22.2	15.7	—
SAM P23110	74.8	21.5	—	—
SAM P17364	73	—	—	—
SAM P24171	80.3	21.4	14.3	—
SAM P24183	—	—	13.6	—
SAM P24139	—	21.4	—	—
SAM P24203	—	20.3	—	—
SAM P24140	—	—	14.9	—
SAM P22862	75.9	21.4	13.9	—
SAM P22870	82.4	23.4	15.8	—
SAM P41606	73.4	20.4	—	—

*Coracoids* (TL, total medial length; OW, omal width; SW, sternal width; MW, midshaft width)

**Table:**

SAM #	TL	OW	SW	MW
SAM P51354	91.7	22.8	27.5	10.8
SAM P51369	93.2	20.6	29.8	11.2
SAM P51352	—	20.9	—	—
SAM P51245	—	22.8	—	—
SAM P24187	81.9	17.8	—	9.6
SAM P41538	88.7	—	—	11.0
SAM P23120	86.3	16.7	—	10.5
SAM P19076	85.9	17.5	—	11.4
SAM P22820	90.6	18.6	27.5	10.9
SAM P23492	—	—	—	10.8
SAM P23336	92.1	17.3	—	12.0
SAM P41663	92.0	17.6	31.4	12.4
SAM P22868	89.6	17.3	—	11.2
SAM P24144	—	—	32.0	—
SAM P41842	—	—	—	10.5
SAM P39650	—	16.7	—	—
SAM P22715	84.2	16.5	—	10.5
SAM P41718	—	17.2	—	—
SAM P41654	—	17.2	—	10.8
SAM P22817	87.2	17.8	—	10.8
SAM P39636	86.9	17.8	—	11.2
SAM P39637	85.2	17.5	27.8	10.4
SAM P39638	84.9	15.5	27.7	10.5
SAM P39645	88.8	16.8	—	10.6

*Scapulae* (WA, width articularis humeralis; WC, width collum scapulae; DC, depth collum scapulae; LAA, length from acromion to distal edge of facies articularis humeralis)

**Table:**

SAM #	WA	WC	DC	LAA
SAM P51368	13.1	10.3	—	24.1
SAM P51369	12.2	11.0	—	25.2
SAM P51366	12.9	11.7	—	—
SAM P41374	12.6	10.3	7.3	—
SAM P41578	13.7	10.8	8.1	23.3
SAM P41696	11.2	10.5	7.6	—
SAM P41591	13.5	11.8	8.5	23.7
SAM P19077	12.2	10.8	8.2	23.7
SAM P39635	12.9	10.8	8.2	25.8
SAM P39652	12.2	12.0	8.1	24.9
SAM P41730	12.2	10.4	7.8	23.4
SAM P23281	12.5	11.0	7.1	24.4
SAM P39648	11.5	10.0	6.9	22.6
SAM P41543	11.0	10.5	7.4	22.7
SAM P41392	10.3	9.7	6.7	—
SAM P41481	12.1	11.8	8.2	24.3
SAM P39634	—	11.3	7.6	25.2
SAM P41717	12.8	10.6	7.2	23.3
SAM P41856	11.7	11.1	6.9	24.4
SAM P41594	12.5	10.0	7.3	23.2
SAM P41686	13.6	11.1	7.7	23.9
SAM P18630	11.7	10.1	7.2	24.8
SAM P22863	12.3	11.3	8.1	—

*Femora* (MC, minimum shaft circumference)

**Table:**

SAM #	TL	PW	SW	DW	MC
SAM P51368	119.4	30.5	14.1	28.2	42.2
SAM P51369	—	—	—	29.6	—
SAM P51263	—	31.7	14	—	44.3
SAM P19070	113.4	30.3	12.3	—	—
SAM P23445	110.7	27.8	12.2	26.5	—
SAM P17857	—	28.4	—	—	—
SAM P51254	—	—	—	—	45
SAM P51262	—	—	—	—	40.7
SAM P22712	108.1	—	12.1	—	37.2
SAM P22852	—	—	12.9	—	—
SAM P23122	102.4	28.6	11.8	25.3	37.2
SAM P23325	—	—	—	25.2	
SAM P23371	—	—	13.2	—	40.7
SAM P24192	—	28.7	—	—	—
SAM P39622	110.8	—	13.2	25.8	41.1
SAM P39633	—	—	—	29.5	—
SAM P41476	—	—	12.9	26.7	—
SAM P41482	—	—	—	27.9	—
SAM P41541	—	—	—	27.2	—
SAM P41592	—	29.8	—	—	—
SAM P41593	—	—	—	26	—
SAM P41729	—	—	14.1	—	43.6
SAM P41741	—	28.4	—	—	—
SAM P41834	—	31.5	13	—	—
SAM P42707	—	—	—	27.3	—

*Tibiotarsi*

**Table:**

SAM #	TL	PW	SW	DW	MC
SAM P51243	170.3	22.9	11.3	22.7	33
SAM P51242	158.3	24	12.4	23.6	34.3
SAM P51239	—	—	—	21.9	—
SAM P51241	—	—	—	22.1	31
SAM P51240	—	—	11.3	21.7	30.9
SAM P17876	—	—	10.7	20.5	—
SAM P17152	157.8	—	11.3	22.5	—
SAM P18633	—	—	—	19.1	—
SAM P19042	—	—	—	19.6	—
SAM P23123	—	—	—	19.8	—
SAM P23152	—	—	—	19.5	—
SAM P23285	—	—	10.5	21.2	28.5
SAM P23286	—	—	11.1	20.7	32.3
SAM P23298	—	21.6	10.5	—	30.7
SAM P23299	—	—	9.9	20.6	29.2
SAM P23326	—	—	—	20.6	—
SAM P24142	—	26.3	13.1	—	—
SAM P24150	—	—	—	23.8	—
SAM P24151	—	—	—	—	—
SAM P24162	—	—	—	21.2	—
SAM P24195	—	—	—	19.9	—
SAM P24197	—	25.2	—	—	—
SAM P24198	—	—	—	19.7	—
SAM P24206	—	—	9.1	19.5	27.4
SAM P31775	—	—	—	22.5	—
SAM P31776	—	—	—	20.7	30.9
SAM P39623	—	22.8	—	—	—
SAM P39624	—	—	—	20.1	—
SAM P41368	—	—	12.6	—	—
SAM P41475	157	25.5	11.4	22.1	31.4
SAM P41477	—	23.3	—	—	—
SAM P41539	—	—	—	19.1	—
SAM P41547	—	—	9.8	20.7	27.8
SAM P41548	—	—	9.4	20.4	26.2
SAM P41659	—	23.7	10.4	21.5	—
SAM P41690	—	—	—	21.8	—
SAM P41691	—	—	11.2	22.4	33.1
SAM P41692	—	—	11.3	—	—
SAM P41720	144.2	22	10.5	19.2	30
SAM P41721	—	—	—	21.1	—
SAM P41722	—	—	—	20	—
SAM P41724	—	24.7	—	—	—
SAM P41737	—	—	10.6	—	—
SAM P41749	—	—	10.5	19.7	31.2
SAM P41835	—	—	—	22.2	33.2

*Tarsometatarsi* (^a^midshaft width, minimum width in this taxon)

**Table:**

SAM #	TL	PW	SW^a^	DW
SAM P51369	—	—	—	25.6
SAM P51235	103.90	—	10.7	—
SAM P51231	105.60	25.00	11.3	27.1
SAM P51233	94.80	24.00	11	—
SAM P51234	97.70	24.30	10.6	26.2
SAM P41579	93.20	21.80	9.3	24.4
SAM P41843	94.60	19.70	9.6	22.8
SAM P41745	90.20	21.90	9.4	24
SAM P41731	104.70	25.30	10.6	28
SAM P41685	102.00	22.70	10	23.7
SAMP41709	96.80	23.00	10.5	26.4
SAM P31779	99.00	—	10.9	24.2
SAM P31778	96.00	21.80	9.8	23.4
SAM P23287	95.60	21.90	9.3	24
SAM P31772	98.90	23.10	—	—
SAM P18185	—	—	10.2	—
SAM P17856	95.30	21.70	9.2	—
SAM P39643	—	25.90	—	—
SAM P39644	—	—	—	24.5
SAM P41740	—	—	—	25.6
SAM P41837	—	—	—	25.4
SAM P41746	89.00	22.50	9.9	—
SAM P41841	—	—	—	24.2
SAM P41748	—	22.10	—	—
SAM P41479	—	25.00	—	—
SAM P24158	—	23.10	—	—
SAM P39646	—	22.50	—	—
SAM P41577	—	—	—	—
SAM P23301	—	—	—	24.1
SAM P22853	96.40	22.50	9.4	24.3
SAM P25633	100.50	24.20	10.4	—
SAM P17361	—	22.70	9.5	—
SAM P23109	—	—	10.2	26.4
SAMP23382	—	—	—	22.7
SAM P24152	—	25.40	10.7	—
SAM P31777	95.00	22.60	9.2	24
SAM P19044	—	22.50	9.6	—
SAM P24207	—	—	—	24.6
SAM P41605	—	22.70	—	—
