# Supplementary material for: Taxonomic review of the late Cenozoic megapodes (Galliformes: Megapodiidae) of Australia
Source: R Soc Open Sci. 2017 Jun 14;4(6):170233. doi: 10.1098/rsos.170233 (PMC5493918; doi:10.1098/rsos.170233)
Supplement: Supplementary Figure1: Type material of Garrdimalga mcnamarai sp. nov. compared with other extinct species [file rsos170233supp3.pdf]

Supplementary Figure 1

Type material of *Garrdimalga mcnamarai* sp. nov. compared with other extinct species

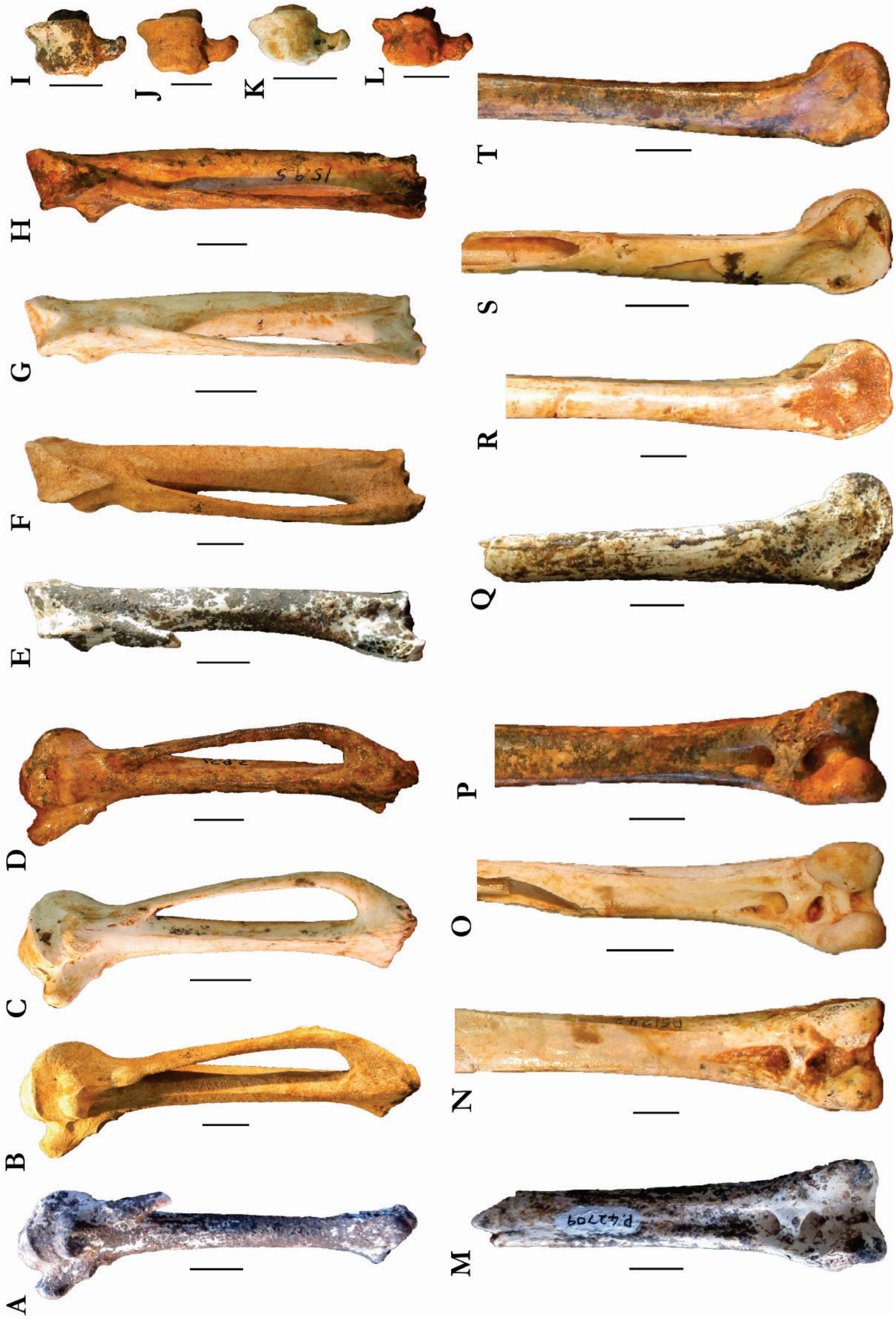

Supp. Figure 1: *Garrdimalga mcnamarai* carpometacarpus, SAM P42711, holotype (A, E, I) and tibiotarsus, SAM P42709, paratype (M, Q). Comparisons with *Latagallina naracoortensis* (B, F, J, N, R), *Latagallina olsoni* (C, G, K, O, S) and *Progura campestris* (D, H, L, P, T).
